# Supplementary material for: Entinostat in combination with nivolumab in metastatic pancreatic ductal adenocarcinoma: a phase 2 clinical trial
Source: Nat Commun. 2024 Nov 12;15:9801. doi: 10.1038/s41467-024-52528-7 (PMC11557583; doi:10.1038/s41467-024-52528-7)
Supplement: Supplementary file 1 — Supplementary Information [file 41467_2024_52528_MOESM1_ESM.pdf]

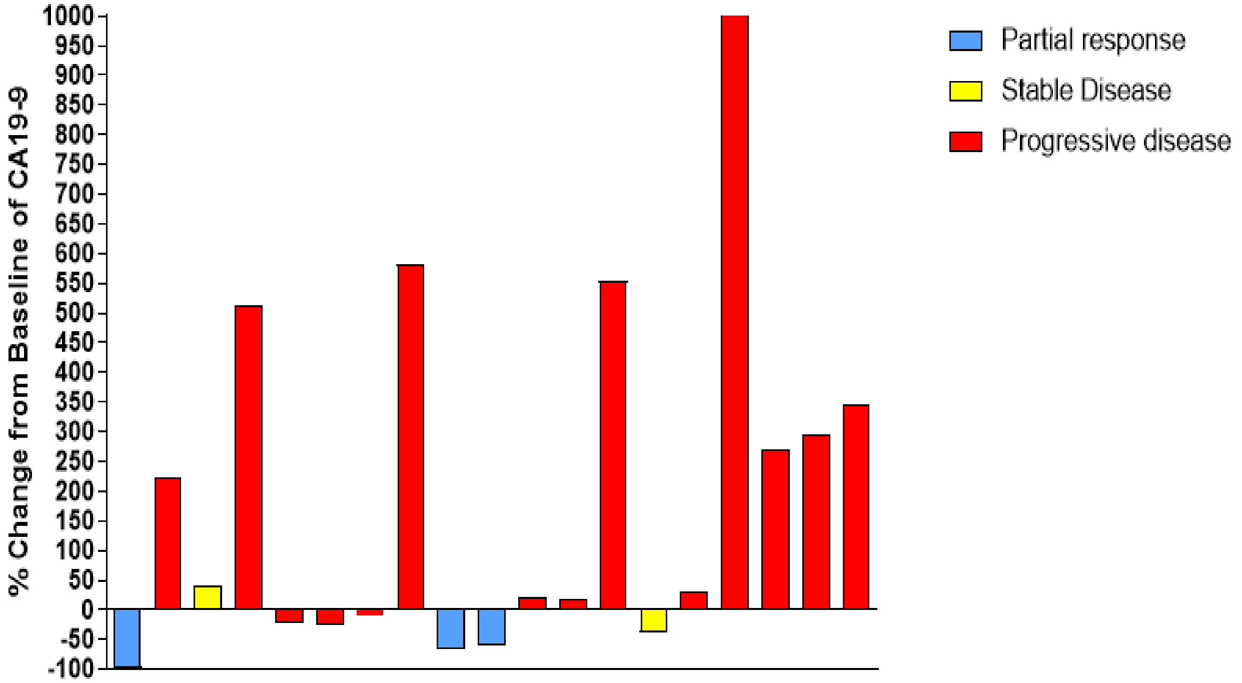

Supplementary Figure. 1: Biochemical response. Change in CA 19.9 tumor marker for all patients with an elevated CA19.9 at baseline (n= 19 patients) and correlation with radiological response.
Source data are provided as a Source Data file.

a

Discovery-23 panel

Lineage	Identification
Neoplastic epithelium	CD45$^-$ PanCK$^+$
CD4$^+$ T lymphocytes	CD45$^+$ CD3$^+$ CD8$^-$ CD4$^+$
Regulatory T cells (Treg)	CD45$^+$ CD3$^+$ CD8$^-$ CD4$^+$ Foxp3$^+$
CD8$^+$ T lymphocytes	CD45$^+$ CD3$^+$ CD8$^+$
CD20$^+$ B cells	CD45$^+$ CD3$^-$ CD20$^+$
Macrophages	CD45$^+$ CD3/CD20$^-$ CD11b$^+$ CD66b$^-$ CD68$^+$ CD169$^+$ CCR2$^-$ CD163$^{+/-}$
Monocytes	CD45$^+$ CD3/CD20$^-$ CD11b$^+$ CD66b$^-$ CD68$^{+/-}$ CD169$^-$ CCR2$^+$ CD163$^{+/-}$
Immature DC	CD45$^+$ CD3/CD20$^-$ CD11b$^{+/-}$ CD66b$^-$ CD68$^-$ CD11c$^+$ HLA class II$^+$ DC-LAMP$^-$
Mature DC	CD45$^+$ CD3/CD20$^-$ CD11b$^{+/-}$ CD66b$^-$ CD68$^-$ CD11c$^+$ HLA class II$^+$ DC-LAMP$^+$
Neutrophils and eosinophils	CD45$^+$ CD3/CD20$^-$ CD11b$^+$ CD66b$^+$

Functional state	Marker
Proliferation	Ki67
Cytotoxicity	Granzyme B$^+$
T cell programming	EOMES, Tbet
Immune regulatory	PD-1, PD-L1, HLA-class I

b

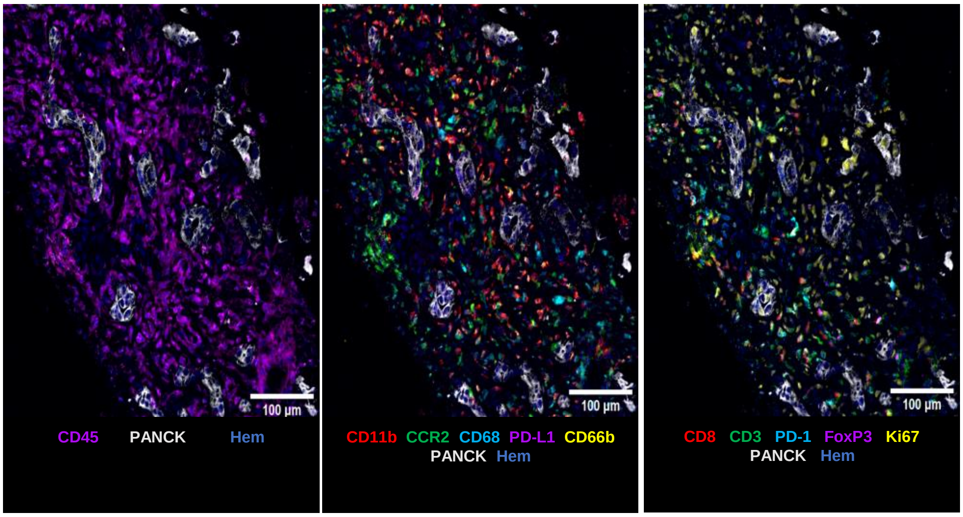

Supplementary Figure 2: : Experimental workflow of 23-biomarker sequential IHC and image visualization. a. Antibody panel and cell identification by hierarchical gating used in mIHC studies. **b.** Example of pseudocolored image of mIHC staining of major immune markers in patient P5 baseline tumor biopsy. All markers shown were stained on the same FFPE tissue, in addition to: HLA-II, DC-LAMP, CD4, Granzyme B, CD20, Tbet, CD11c, CD163, HLA-I, CD169, EOMES. Whole slide images taken at 20x at 2 pixels/um resolution; Pseudocolored images here are 1000 x 1000 pixels.

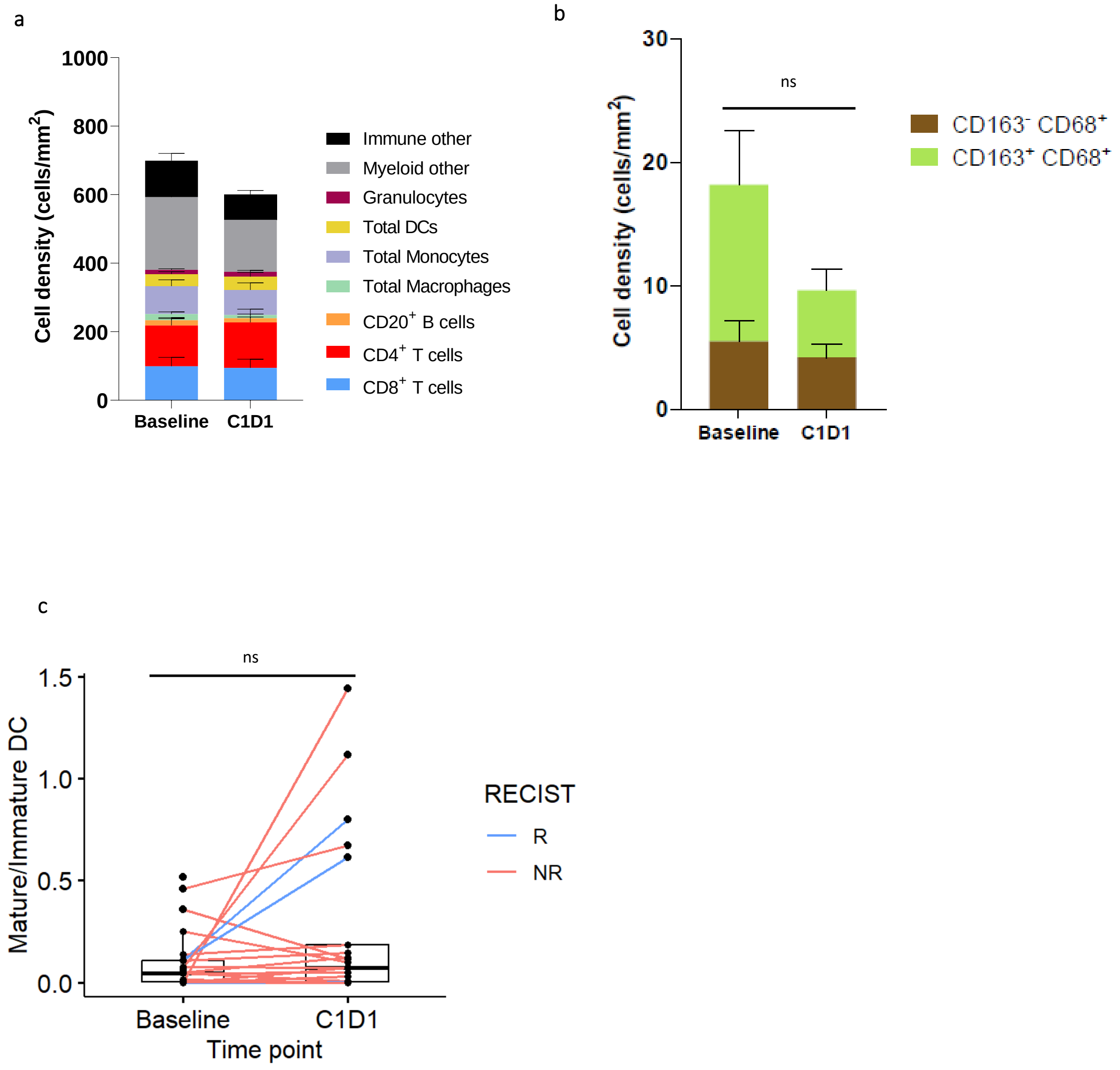

Supplementary Figure 3: Myeloid reprogramming with entinostat therapy. a, Immune composition comparing baseline and C1D1 tumor biopsies. **b,** Trend of decreased CD163$^+$ macrophage density in C1D1 compared to baseline in all samples. Bars represent averages, error bars represent SEM. **c,** Trend of increased ratio of mature (LAMP+):immature(LAMP-) DCs in C1D1 compared to baseline (n = 21 biologically independent pairs of samples, for all panels). Statistics: P-values from paired two-sided Wilcoxon test between time points. Box plots show the median and upper and lower quartiles and whiskers extend to 1.5× the interquartile range. Box plots show the median and upper and lower quartiles and whiskers extend to 1.5× the interquartile range. Source data are provided as a Source Data file.

NR, non-responders; ns, not statistically significant; R, responders

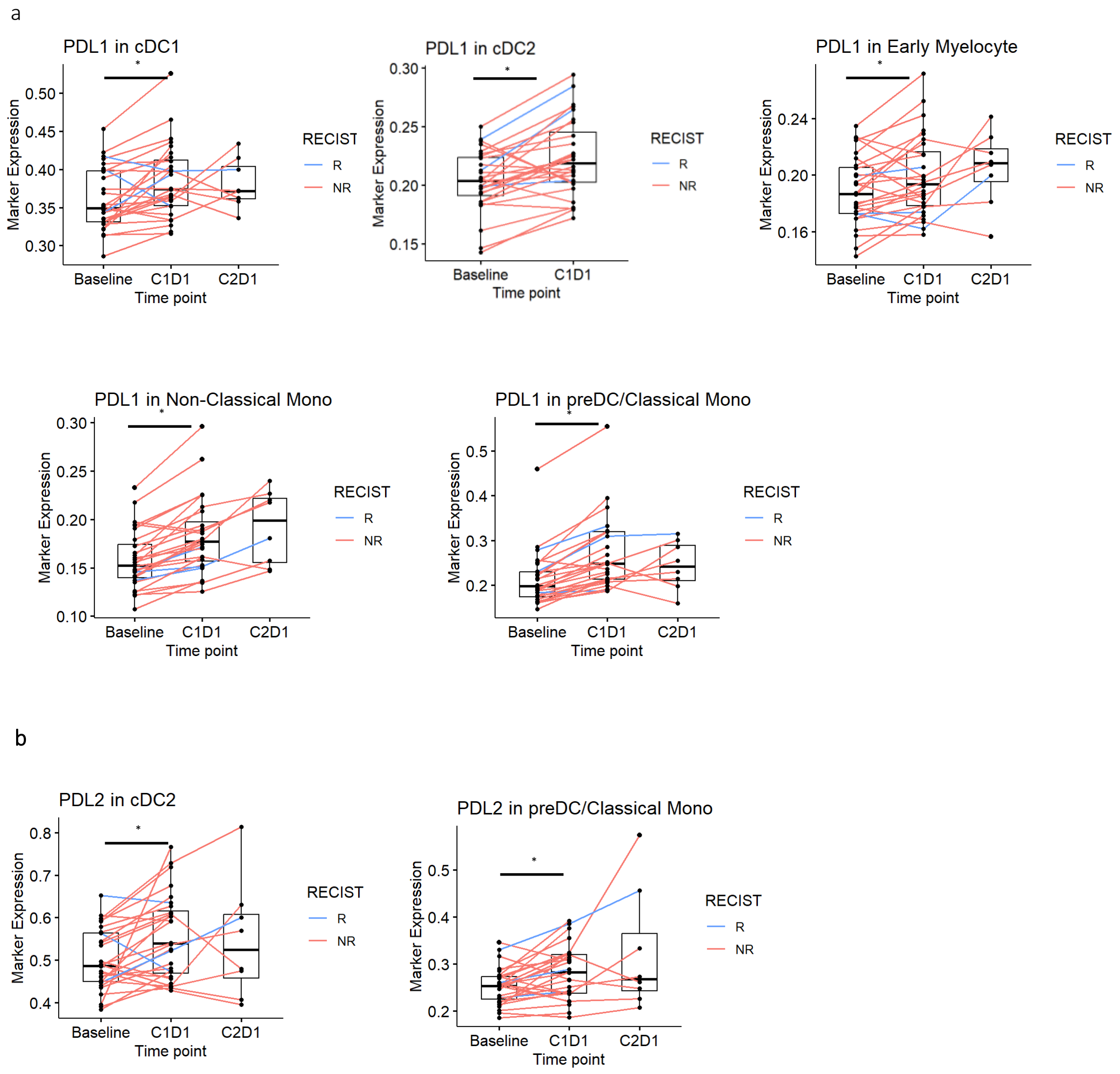

Supplementary Figure 4: PD-L1 and PD-L2 expression across cellular groups in the myeloid partition in the periphery at given timepoints and shown by treatment response subtype. a, PD-L1 expression in cDC1 (p=0.001), cDC2 (p<0.001) , early myelocytes (p=0.012), Non-Classical Mono (p<0.001) and pre-DC-Classical Mono (p<0.001) b, PD-L2 expression in cDC2 (p=0.001), in preDC/Classical Mono (p= 0.005). Significance was determined by paired two-sided Wilcoxon signed-rank test on 28 biologically independent pairs of samples comparing baseline vs post-entinostat run-in (C1D1). Statistically significant P values are shown as follows: *, P < 0.05. Each line corresponds to one patient. Red line corresponds to a non-responder; blue line corresponds to a responder. N = 28 paired patient sample between baseline and C1D1, and n = 8 paired samples between C1D1 and C2D1.
Box plots show the median and upper and lower quartiles and whiskers extend to 1.5× the interquartile range. Box plots show the median and upper and lower quartiles and whiskers extend to 1.5× the interquartile range.
Source data are provided as a Source Data file.
NR, non-responders; R, responders.

a

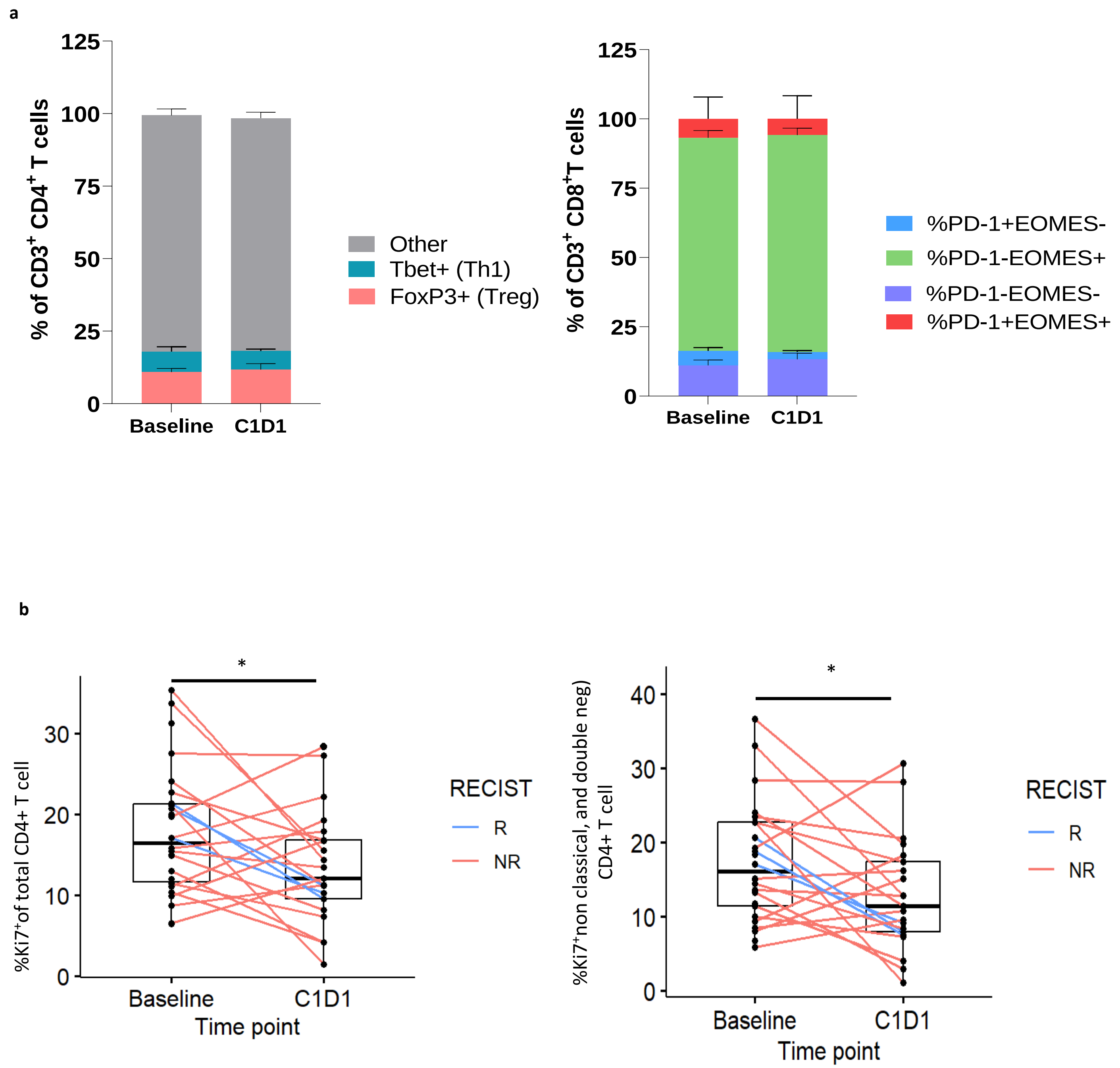

Supplementary Figure. 5: Effect of entinostat on T cells. **a**, relative percentages of major CD4+ (left) and CD8+ (right) T cells subsets at baseline compared with after treatment. **b.** Decreased proliferation (Ki67+) in CD4+T cells (p=0.042) and other (non classical, double negative) T cells (P=0.029 in C1D1 (after entinostat run-in) compared to baseline. Significance was determined by paired two-sided Wilcoxon signed-rank test on 28 biologically independent pairs of samples comparing baseline vs post-entinostat run-in (C1D1). Statistically significant P values are shown as follows: *, P < 0.05. Box plots show the median and upper and lower quartiles and whiskers extend to 1.5× the interquartile range. Box plots show the median and upper and lower quartiles and whiskers extend to 1.5× the interquartile range. Source data are provided as a Source Data file.

Th1, T helper 1; Treg, regulatory T cells

a

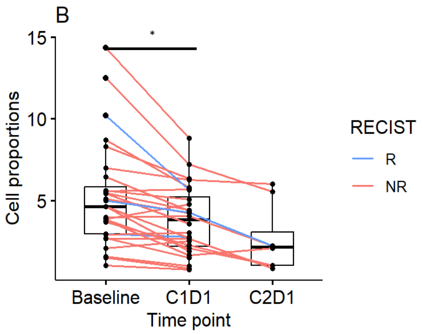

b

Marker	Mean (C1D1- Baseline)	pValue
CD19	0.093	<0.001
CD117	0.009	<0.001
CD11B	0.019	0.002
CD1C	0.12	0.001
CCR2	0.019	0.018
CLEC9A	0.013	0.016
CD11C	0.081	<0.001
CD141	0.004	0.007
CD80	0.008	0.018
TLR4	0.007	0.011
CD172AB	0.014	0.019
CD40	-0.263	<0.001
CD206	-0.027	<0.001
HLADR	-0.164	<0.001
CD68	-0.015	0.012
PD-L1	0.015	<0.001
CD123	-0.045	<0.001

Supplementary Figure 6: Decreased proportion of B cells in post-entinostat peripheral blood. a, Graph depicts percent of proportion of B cells at different timepoints as assessed by CyTOF with the myeloid panel. **b,** Statistically significant expression of markers in B cells population at baseline vs post-entinostat run-in (C1D1) (n = 28 biologically independent pairs of samples) (p= 0.001). Significance was determined by paired two-sided Wilcoxon signed-rank test. Each line corresponds to one patient. Red lines correspond to non-responders, blue lines to responders. Box plots show the median and upper and lower quartiles
and whiskers extend to 1.5× the interquartile range. Box plots show the median and upper and lower quartiles and whiskers extend to 1.5× the interquartile range.
Source data are provided as a Source Data file.
NR, non-responders; R, responders.

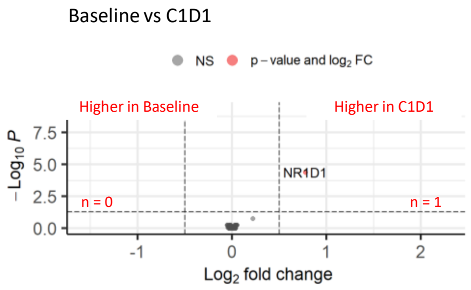

Supplementary Figure 7. RNA-seq analysis of differentially expressed genes between baseline and C1D1 samples. Volcano plots indicating differentially expressed genes following a paired analysis of samples between baseline and C1D1 (n = 16 biologically independent pairs of samples). Log10-transformed adjusted p-values are on the y-axis, and log2-transfomed fold change between time points is on the x-axis. Genes with the absolute log2-fold changes greater than 0.5 and with an adjusted p-value below 0.05 are shown.
Source data are provided as a Source Data file.

Cover Page for Protocol

NCT Number:	NCT03250273
Official title of study:	A Phase 2 Clinical Trial of Entinostat in Combination with Nivolumab for Patients with Previously Treated Unresectable or Metastatic Cholangiocarcinoma and Pancreatic Adenocarcinoma
Document Date:	May 21, 2020

TITLE: A Phase 2 Clinical Trial of Entinostat in Combination with Nivolumab for Patients with Previously Treated Unresectable or Metastatic Cholangiocarcinoma and Pancreatic Adenocarcinoma

**Protocol number #:**	J1798/ IRB00142149
**BMS Protocol #:**	CA209-9L6
**Syndax Protocol #:**	SNDX-275-0205
**Principal Investigator**	Nilofer Azad, MD<br>Sidney Kimmel Comprehensive Cancer Center<br>at Johns Hopkins<br>[redacted]
**Regulatory Specialist:**	Jennifer Durham, PhD, [redacted]
	Rose Parkinson, PhD, [redacted]
**Biostatistics**:	Leslie Cope
**Lead Study Coordinator:**	Alissa Rivas-Morello
**Lead Research Nurse:**	Rosalind Walker, BSN
**BMS Supplied Agent:**	Nivolumab (OPDIVO®, BMS-936558)
**Syndax Supplied Agent**	Entinostat (MS-275, SNDX-275)
**IND:**	IND 136051
**IND Sponsor:**	Nilofer Azad, MD<br>[redacted]

**Version:**	Original/ Version 1/July 5, 2017<br>Original/ Version 1.1/July 27, 2017<br>Original/ Version 1.2/July 28, 2017<br>Original/ Version 1.3/August 11, 2017<br>Original/ Version 1.4/October 10, 2017<br>Amendment 1/ Version 2.0/March 19, 2018<br>Amendment 2/ Version 3.0/April 25, 2019<br>Amendment 3/Version 4.0/October 10, 2019<br>Amendment 3/Version 5.0/May 21, 2020

STUDY GLOSSARY

Abbreviation/Acronym	Definition
J1798/Version 5.0/May 21, 2020	
AE	Adverse event
ALP	Alkaline phosphatase
ALT	Alanine aminotransferase
ANC	Absolute neutrophil count
aPTT	Activated partial thromboplastin time
AST	Aspartate aminotransferase
AZA	5-Azacitadine
BSA	Body surface area
BUN	Blood urea nitrogen
CBC	Complete Blood Count
CI	Confidence interval
CNS	Central nervous system
CpG	Dinucleotides containing cytosine and guanine
CR	Complete response
CrCl	Creatinine clearance
CT	Computed tomography
CTCAE	Common Terminology Criteria for Adverse Events
CTLA-4	Cytotoxic T-lymphocyte-associated antigen-4
DKA	Diabetic ketoacidosis
DNA	Deoxyribonucleic acid

STUDY GLOSSARY

Abbreviation/Acronym	Definition
EC	Ethics Committee
ECG	Electrocardiogram
ECI	Events of clinical interest
ECOG	Eastern Cooperative Oncology Group
eCRF	Electronic case report form
EDC	Electronic data capture
EMA	European Medicines Agency
EOT	End of Treatment
FDG	18F-deoxyglucose
FGFR	Fibroblast Growth Factor Receptor
FoxP3	Forkhead box P3
G-CSF	Granulocyte-colony stimulating factor
GI	Gastrointestinal
GM-CSF	Granulocyte macrophage-colony stimulating factor
G-MDSCs	Granulocytic myeloid-derived suppressor cells
HDAC	Histone deacetylase
HIV	Human immunodeficiency virus
IB	Investigator’s Brochure
ICH	International Conference on Harmonisation
IFN-□	Interferon-gamma
Ig	Immunoglobulin
IND	Investigational New Drug Application

STUDY GLOSSARY

Abbreviation/Acronym	Definition
INR	International normalized ratio
irAE	Immune-related adverse events
IRB	Institutional Review Board
irRECIST	Immune-related Response Evaluation Criteria in Solid Tumors
IV	Intravenous(ly)
LDH	Lactic dehydrogenase
mAb	Monoclonal antibody
MDSC	Myeloid-derived suppressor cells
MRI	Magnetic resonance imaging
MTD	Maximum tolerated dose
NCI	National Cancer Institute
NSCLC	Non-small cell lung cancer
NYHA	New York Heart Association
ORR	Overall response rate
OS	Overall survival
PBMCs	Peripheral Blood Mononuclear Cells
PD	Progressive disease
PDAC	Pancreatic Ductal Adenocarcinoma
PD-1	Programmed death receptor-1
PD-L1	Programmed death ligand-1
PD-L2	Programmed death ligand-2
PET	Positron emission tomography

STUDY GLOSSARY

Abbreviation/Acronym	Definition
PFS	Progression-free survival
PK	Pharmacokinetic
PO	By mouth
PR	Partial response
PT	Prothrombin time
RBC	Red blood cell (count)
RECIST	Response Evaluation Criteria in Solid Tumors
SAE	Serious adverse event
SC	Subcutaneous(ly)
SD	Stable Disease
SOC	System organ class
T1DM	Type 1 diabetes mellitus
T3	Triiodothyronine
T4	Thyroxine
TEAEs	Treatment-emergent adverse events
TGF	Transforming growth factor
TIL	Tumor-infiltrating lymphocyte
TKI	Tyrosine kinase inhibitor
TME	Tumor microenvironment
TSH	Thyroid stimulating hormone
ULN	Upper limit of normal
WBC	White blood cell (count)

STUDY GLOSSARY

Abbreviation/Acronym	Definition
WOCBP	Women of child-bearing potential

SCHEMA

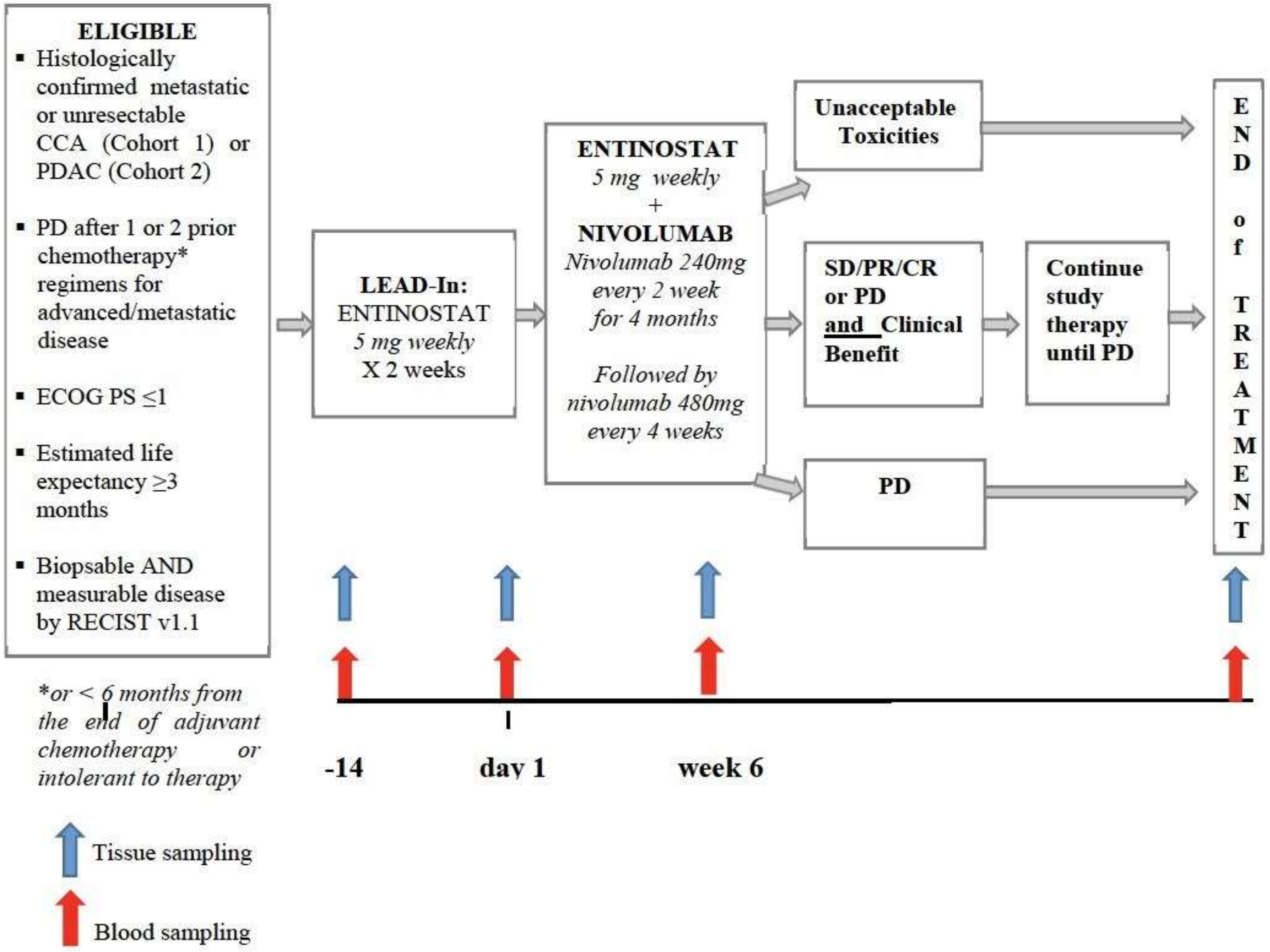

Mandatory research bloods and tumor biopsies will be obtained at baseline (day -14), after entinostat lead in (on approximately day 14 of entinostat therapy), and approximately 6 weeks after the beginning of the combination treatment of entinostat plus nivolumab.

An optional biopsy will be performed at the time of progression.

SD, Stable Disease; PR, Partial Response; CR, Complete Response; PD, Progression Disease

STUDY GLOSSARY ....
2 SCHEMA
.. 6 1.
OBJECTIVES 10

1.1 Primary Objective 10
1.2 Secondary Objectives.. 10
1.3 Exploratory Objectives 10
1.4 Primary Endpoint 11
1.5 Secondary Endpoints 11
1.6 Exploratory Endpoints 11

2. BACKGROUND AND RATIONAL ....
12

2.1 Disease(s) Background 12
2.2 Immunotherapy 13
2.3 Epigenetics Changes as a Target for New Therapy 15
2.4 Immunotherapy as an Anticancer Strategy in PDAC and CCA 18
2.5 Investigational Agents 20
2.6 Entinostat in Combination with PD-1 Checkpoint Inhibition: Rational of the Study 26

3. PATIENT SELECTION 33

3.1 Eligibility Criteria 33
3.2 Exclusion Criteria 34
3.3 Inclusion of Women and Minorities 37

4. TREATMENT PLAN ....
38

4.1 Agents Administration 38
4.2 Overall Study Design 38

4.3 Definition of Unacceptable Toxicities 39
4.4 Study Drugs 40
4.5 Supportive Care Guidelines and General Concomitant Medication 41
4.6 Definition of an Overdose for this Protocol 45
4.7 WOCBP, Contraception, Use in Pregnancy, Use in Nursing 45
4.8 Duration of Treatment and Criteria for Removal from Treatment 48
4.9 Treatment Beyond Progression 49
4.10 Duration of Follow Up 50
5. DOSING DELAYS/DOSE MODIFICATIONS 51
5.1 Dose Criteria 51
6. ADVERSE EVENTS: LIST AND REPORTING REQUIREMENTS 62
6.1 Study Drugs 62
6.2 Adverse Events Definition 63
6.3 Reporting Procedures for All Adverse Events 65
6.4 Serious Adverse Event Reporting Procedures 67
6.5 Pregnancy and Lactation Reporting Procedures 68
6.6 Reporting of Overdose to the Sponsor 69
6.7 Follow-Up of Adverse Events 69
6.8 Reconciliation of SAEs........69
6.9 Safety Reporting to Health Authorities, Ethics Committees/Institutional Review Boards and Investigators 70
6.10 Food and Drug Administration (FDA) Reporting 70
6.11 Protocol Deviations Due to an Emergency or Adverse Event 71
6.12 Safety Monitoring 71

7. PHARMACEUTICAL INFORMATION 72

7.1 Entinostat (NSC #706995) 72

7.2 Nivolumab (NSC #748726) 73

8. MEASUREMENT OF EFFECT 76

8.1 Antitumor Effect – Solid Tumors (RECIST 1.1) 76

8.2 Definitions.. 77

8.3 Response Criteria 80

8.4 Safety/tolerability 84

9. BIOMARKER, CORRELATIVE, AND SPECIAL STUDIES 85

9.1 Overview 85

9.2 Collection of Specimens 85

9.3 Methods.. 87

10. STUDY CALENDAR 89

11. DATA REPORTING / REGULATORY REQUIREMENTS 95

11.1 Data Management 95

11.2 Data Confidentiality and Subject Anonymity 95

11.3 Protocol Compliance 95

11.4 Safety meetings 96

11.5 Monitoring 96

12. STATISTICAL CONSIDERATIONS 97

12.1 Overall Study Design 97

12.2 Sample Size and Accrual Rate 97
12.3 Study Endpoints Analyses 98
13. REFERENCES .... 101 14. APPENDICES 111
Appendix A: Eastern Cooperative Oncology Group Performance Status Scale, with Equivalent Karnofsky Performance Status Scores 111
Appendix B: Patient Drug Information Handout and Wallet Card 112
Appendix C: Entinostat Drug Diary 114
Appendix D: Management Algorithms for Nivolumab -Related Adverse Events 115
Appendix E: Concomitant Medications to be Avoided 123
Appendix F: Serious Adverse Event Reporting Form 124
Appendix G: Nivolumab Product Information Table 126

1. OBJECTIVES

1.1 Primary Objective

1.1.1 To determine the objective response rate (ORR) of entinostat plus nivolumab in patients with unresectable or metastatic cholangiocarcinoma and unresectable or metastatic pancreatic adenocarcinoma, who have progressed after one more two lines of prior therapy.

1.2 Secondary Objectives

1.2.1 To assess the safety and tolerability of entinostat plus nivolumab in patients with unresectable or metastatic cholangiocarcinoma and unresectable or metastatic pancreatic adenocarcinoma.

1.2.2 To assess the progression free survival (PFS) status of patients with unresectable or metastatic cholangiocarcinoma and unresectable or metastatic pancreatic adenocarcinoma treated with entinostat plus nivolumab at 6, 12 and 24 months.

1.2.3 To determine the overall survival (OS) status of subjects with unresectable or metastatic cholangiocarcinoma and unresectable or metastatic pancreatic adenocarcinoma treated with entinostat plus nivolumab.

1.2.4 To determine the overall survival (OS) status of subjects with unresectable or metastatic cholangiocarcinoma and unresectable or metastatic pancreatic adenocarcinoma treated with entinostat plus nivolumab at 6 months, at 1, 2 and 3 years.

1.2.5 To assess the duration of response among subjects who demonstrate an objective response to treatment with entinostat in combination with nivolumab.

1.3 Exploratory Objectives

1.3.1 To measure the baseline levels of immune markers including CD8+ T effector cells and CD4+FoxP3+ T regulatory cells, PD-L1 expression and tumor infiltrating lymphocytes (TILs), major histocompatibility complex (MHC) class I and II expression, and natural killer (NK) cell receptors and ligands expression and correlate these variables with treatment response and toxicity.

1.3.2 To characterize changes in the immune markers described above after treatment

1.3.3 To identify gene expression changes in malignant tissue after therapy, and complete gene set analysis to elucidate affected pathways

1.3.4 To measure changes in circulating immune suppressor cells (myeloid derived suppressor cells (MDSC), Treg) in peripheral blood by flow cytometry and test for association with response to therapy.

1.3.5 To assess the baseline characteristics of the subjects enrolled and to correlate these molecular and clinicopathologic criteria with treatment response and toxicity.

1.4 Primary Endpoint

1.4.1 ORR, defined as the proportion of patients achieving a complete response (CR) or partial response (PR) based on the Response Evaluation Criteria in Solid Tumors (RECIST 1.1) at any time during the study, of entinostat in combination with nivolumab in patients with unresectable or metastatic cholangiocarcinoma and unresectable or metastatic pancreatic adenocarcinoma.

1.5 Secondary Endpoints

1.5.1 Adverse events as graded by NCI CTCAE active version.

1.5.2 Progression Free Survival (PFS) status at 6, 12 and 24 months.

1.5.3 Duration of Response (DOR).

1.5.4 Overall Survival (OS).

1.5.5 OS at 6 months, at 1, 2 and 3 years

1.6 Exploratory Endpoints

1.6.1 Changes in immune-related biomarkers (e.g., expression of checkpoint receptors (PD1/PD-L1), the number of MDSCs, inflammatory T cell signature, in tumor biopsies and peripheral blood pre- and post-therapy and their correlation with response to therapy.

1.6.2 Gene expression changes in circulating DNA and malignant tissue after therapy, and complete gene set analysis to elucidate affected pathways

1.6.3 Changes in circulating immune suppressor cells (MDSC, Treg) in peripheral blood by flow cytometry and test for association with response to therapy

2. BACKGROUND AND RATIONAL

2.1 Disease(s) Background

2.1.1 Pancreatic Adenocarcinoma

Pancreatic ductal adenocarcinoma (PDAC) constitutes one of the most aggressive malignancies with a 5-year survival rate of <7%, due to growing incidence, late diagnosis and insufficient treatment options [1]. Although intensified cytostatic combinations, particularly gemcitabine plus nabpaclitaxel and the folinic acid, fluorouracil, irinotecan, oxaliplatin (FOLFIRINOX) protocol, provide some improvement in efficacy and survival compared with gemcitabine alone, a breakthrough in the treatment of metastatic pancreatic cancer remains out of sight [2, 3]. The nanoliposomal formulation of irinotecan, MM-398, in combinations with 5FU resulted in a two month improvement in median survival compared to 5FU and leucovorin [4]. Similarly the combination of weekly 5FU, leucovorin and oxaliplatin after gemcitabine failure resulted in a median overall survival of 5.9 months versus 3.3 months for weekly 5FU and leucovorin in the CONKO-003 trial [5]. However, a more recent trial showed no difference in outcome with 5FU versus 5FU and oxaliplatin in the same setting [6].

Based on this, there is an urgent need for the development of more effective therapies in second line and beyond for advanced PDAC. One of the emerging interests in PDAC therapy is immunotherapy because these tumors generally escape immune surveillance through various mechanisms [7]. Unfortunately to date, monotherapy with immune checkpoint blockade that have being investigated, resulted ineffective in PDAC.

Given the great promise of immune-based therapies in other malignancies, there is reason to hope that modulating the tumor microenvironment could also augment tumor control in this context (see **Sections 2.4** and **2.5**).

2.1.2 Cholangiocarcinoma

Cholangiocarcinoma (CCA) is the most common biliary tract malignancy and the second most common primary hepatic malignancy. It is classified into intrahepatic (iCCA) and extrahepatic cholangiocarcinoma (eCCA) subtypes, the latter often further divided in perihilar (pCCA), and distal (dCCA). The overall prognosis of CCA is dismal, with surgery as the only option for cure, but given the propensity for early metastasis and high recurrence, 5-year OS ranges from 14 to 40 % after surgical resection [8, 9]. In the setting of metastatic disease we still have limited treatment options, and so far the combination of gemcitabine and cisplatin has been established as the standard of care first line systemic therapy option with a median survival of 11 months [10]. There are no established second line treatment options [9, 11].

This huge unmet clinical need supports exploring novel therapies. Our understanding of the molecular tumor biology of CCA has been increased in the last years. For instance, Isocitrate Dehydrogenase 1 or 2 (*IDH1/2*), *BAP1* mutations and *FGFR* fusions are more likely to occur in iCCA while *KRAS*, *p53*, and *SMAD4* mutations are more common in eCCA. The mutational aberrations landscape vary with etiology, ethnicity and association with chronic liver disease too [9, 11]. Several clinical trials have demonstrated the potential role of monoclonal antibodies in improving patient outcomes via the targeting of interactions between immune regulatory checkpoint molecules and their ligands.

Despite this growing body of evidence, it remains unknown whether this adaptive immune resistance pathway is relevant among patients with CCA and whether similar novel therapies might eventually be useful as adjunct therapies for these patients.
Several data suggest that the Programmed Death-1 (PD-1) and its ligands (Programmed DeathLigand 1, PD-L1) pathway may be actively suppressing the host immune response in patients with ICC and can be a potential target for future therapies, providing a rationale for clinical trials to assess the efficacy and safety of anti-PD-1/PD-L1 therapies among patients with ICC (see **Section**
2.4 and **2.5**).

2.2 Immunotherapy

Immunotherapy is defined as the approach to treating cancer by generating or augmenting an immune response against it. Immunomodulation is based on the finding that stimulation of

T-cell function with antibodies that block or activate regulatory receptors, can cause the regression of some tumors. The generation of immunological memory is another unique feature of immune modulation as an effective cancer therapy [12].

As far, antitumor immunotherapy has broad potential and could be used to treat many different types of advanced stage cancers owing to the durable and robust responses it elicits across a diverse spectrum of malignancies. Recent evidence highlights the pivotal role of the anti-cytotoxic T-cell lymphocyte-associated protein 4 (CTLA-4) receptor, and PD-1 T cell co-receptor and its ligands B7-H1/PD-L1 and B7-DC/PD-L2 in maintaining an immunosuppressive tumor microenvironment (TME), as part of the so called immune checkpoints family [13-15]. Immune-cell-targeted monoclonal antibodies (mAbs) therapy against these checkpoints inhibitors has emerged as particularly effective, having the potential to enhance and sustain endogenous immunity against tumor antigens and establishing durable tumor control.
Clinical trials with anti–CTLA-4, anti–PD-1, and anti–PD-L1 mAbs, showed remarkable therapeutic responses [16-19], underscoring the idea that disruption of immune checkpoints can be therapeutically useful.

Since, immunomodulatory mAbs target immune cells rather than cancer cells, we expect they are not necessarily specific to any cancer type. However, despite the breakthroughs of the past decade, the successes to date do not fully capture the promise of immunotherapy: the objective responses are observed in a minority of the treated patients and tumor types, and the reasons why certain tumors respond and others do not are not fully understood [20].

It has been hypothesized that some tumor types with a more "immunogenic profile" that naturally attract T cell infiltration, are more likely to respond to checkpoint blockade. Evidence for this hypothesis is supported by data from studies in which a subset of lung adenocarcinomas with higher levels of somatic mutations had increased levels of inflammation-related gene expression and immune-checkpoint effector molecules, including PD-L1 and the evidence that the prevalence of neoantigens can be predictive of the response to checkpoint blockade with PD-1-targeted therapy in patients with non-small cell lung cancer (NSCLC), and with CTLA-4-targeted therapy in those with melanoma [21, 22].

Conversely, tumors with low neoantigen-presenting capacity, such as those that have a reduced number of potentially immunogenic somatic mutations or that do not present neoantigens through downregulated antigen processing, presentation or HLA expression might be overlooked by endogenous T cells [12, 23]. In this situation, antigen presentation and tumor-infiltrating lymphocytes (TILs) burden will likely be low, and immune-modulating mAbs alone would be less likely to generate a robust antitumor response, unless it is administered in the proper therapeutic context.

For instance, CCA and PDAC, has generally been considered a nonimmunogenic malignancy, insofar as tumor-infiltrating effector T lymphocytes do not represent a histopathologic hallmark of this disease. To date, studies of anti–CTLA-4 and anti–PD-1 mAbs have failed to demonstrate any objective responses in this setting, unless in the small fraction (about 3% of PDAC) of patients with hypermutated tumors and microsatellite instability [24].

Strategies to sensitize these tumors to checkpoint inhibition are warranted and the epigenetic modulation seem to play a very important role in this framework.

2.3 Epigenetics Changes as a Target for New Therapy

The properties of a cell are determined both by its genetic information and by the pattern in which its genes are expressed. This pattern of expression must be heritable itself, and also highly adaptable. The non-genetic determinants that precisely control the patterns of gene expression in cells are commonly referred to as epigenetic mechanisms [25].
Cancer cells represent a complex interplay between genetic and epigenetic abnormalities that, from beginning to end, drive the evolution of each patient's malignancy. It has been demonstrated that virtually all tumors harbour mutations in genes that control the epigenome [26].

Four major mechanisms are involved in the epigenetic regulation of gene expression patterns: covalent modification of DNA; covalent modification of histones; non-protein-coding RNAs (microRNAs –miRNAs-) and long non-coding RNAs (lncRNAs). Currently, the most studied and recognized cancer-specific epigenetic changes are alterations in DNA methylation and histone acetylation [27-29].

Cancer-specific hypermethylation of hundreds of genes per tumor, typically involves normally unmethylated, dinucleotides containing cytosine and guanine (CpG) -enriched DNA sequences (so called the CpG islands) that reside in and around proximal gene promoters [27]. This hypermethylation can be associated with decreased gene expression, thus providing an alternative way to silence well known tumor suppressor genes. This mechanism is nowadays recognized as an early and central event in carcinogenesis [28, 29].

Additionally, cancer-specific epigenetic chromatin events involve modifications of histone proteins, including histone deacetylation as well as decreases in methylation at lysine 4 of histone 3 (H3K4an), that is known to be an active mark of gene transcription at promoters of DNA hypermethylated genes [30, 31]. These changes led to an important change in chromatin conformation, resulting in a close configuration and, indeed, in a decreased gene expression, even in the absence of DNA methylation.

These epigenetic changes contribute to tumorigenesis and to resistance to common therapies of many types of cancer, including PDAC and CCA. A number of elegantly conducted translational studies have provided significant evidence that dysregulation of chromatin organization strongly contributes to the aggressive behavior of PDAC and CCA, by promoting cellular plasticity and therapeutic resistance. In addition, whole genomic sequencing revealed pathogenic mutations and structural variants in several epigenetic regulator genes in these cancer types [32, 33].

Consequently, several clinical trials have been initiated to determine the safety and efficiency of epigenetic drugs in patients with PDAC and CCA.DNA methyltransferase and HDAC inhibitors have been in clinical development as single agents for a number of years. Three HDAC inhibitors, vorinostat (Zolinza), romidepsin (Istodax) and panobinostat (Farydak) have reached approval by the US Food and Drug Administration (FDA) or by the European Medicines Agency (EMA) for the treatment of cutaneous T-cell lymphoma (vorinostat and romidepsin), peripheral T-cell lymphoma (romidepsin) and multiple myeloma (vorinostat and panobinostat). The presumptive mechanism of action of these drugs is the activation of tumor suppressor genes or immunityassociated genes silenced in tumor cells [34, 35].

Study by Hopkins scientists discovered another important mechanism: the induction of a substantial reduction of the number of tumor-infiltrating myeloid derived suppressor cells (MDSCs) especially when combined with immune checkpoint blockade, as discussed below [36].

2.3.1 HDAC Inhibitors

Histone acetylation is controlled by a balance in activity between histone acetyltransferase (HAT) and HDAC. HDAC inhibitors are critically important in the regulation of gene expression and in the field of target-specific anticancer drug development [34, 35, 37, 38]. The HDACs exert their targeted action during post-translational acetylation of core nucleosomal histones, which affects chromatin structure, thereby regulating gene expression.

DNA that is wrapped around condensed, non-acetylated histones is transcriptionally inactive, whereas acetylation of N-terminal histone lysine residues exposes DNA to important transcription factors that promote transcriptional activity. The action of HDACs on nucleosomal histones leads

to tight coiling of chromatin and silencing of expression of various genes, including those implicated in the regulation of cell survival, proliferation, differentiation, and apoptosis [34, 35].

The effects of HDACs are not limited to histone deacetylation. HDACs also act as members of a protein complex to recruit transcription factors to the promoter region of genes, including those of tumor suppressors, and they affect the acetylation status of specific cell cycle regulatory proteins. Because aberrant HDAC activity has been implicated in a variety of cancers, development of HDAC inhibitors is a rational approach to the design of targeted anticancer therapeutics [35]. Several HDAC inhibitors have been studied in phase 1 and 2 trials in hematological malignancies and solid tumors. A number of recent observations have led to the suggestion that the antitumor activity of HDACi may be in part due also to their immunomodulatory properties, by regulating cytokine production, altering activation and function of macrophage and dendritic cells, regulating the transcription of major histocompatibility class I and II, or the activation of costimulatory molecules, and increasing the function of Tregs and enhancing their immunosuppressive effect in vivo [28, 29, 35].

Recent preclinical investigations highlight the Tregs-targeting and immune-promoting effect of a class I specific HDAC inhibitor, entinostat, in combination with either IL-2 in a murine renal cell carcinoma (RENCA) model or a surviving based vaccine therapy (SurVaxM) in a castration resistant prostate cancer (CR Myc-CaP) model [39].

2.3.1.1 Rationale for use HDAC inhibitors in cholangiocarcinoma

Expression of HDACs was studied in CCA cell lines (M213, M214 and KKU-100) and an immortal cholangiocyte (MMNK1) by semi-quantitative reverse transcription-PCR, demonstrating that HDACs were expressed in all studied cell types. HDAC inhibitors in this study inhibited cell proliferation in a dose-dependent manner. Interestingly, KKU-100 which was less sensitive to classical chemotherapeutic 5-FU was highly sensitive to HDAC inhibitors. Simultaneous combination of subtoxic doses of HDAC inhibitors and 5-FU significantly inhibited cell proliferation in CCA cell lines compared to single agent treatment, showing a synergistic antitumor potential of simultaneous combination of HDAC inhibitors and cytotoxic chemotherapy [40].

Another study investigate the antiproliferative effect of the HDAC inhibitor, entinostat, on CCA cells alone and in combination with conventional cytostatic drugs (gemcitabine or doxorubicin) or the novel anticancer agents sorafenib or bortezomib, using two human bile duct adenocarcinoma cell lines (EGI-1 and TFK-1). They demonstrated that entinostat treatment potently inhibited the proliferation of EGI-1 and TFK-1 CCA cells by inducing apoptosis and cell cycle arrest. Entinostat-induced apoptosis was characterized by activation of caspase-3, up-regulation of Bax and down-regulation of Bcl-2. Cell cycle was predominantly arrested at the G1/S checkpoint, which was associated with induction of the cyclin-dependent kinase inhibitor p21Waf/CIP1.
Furthermore, additive anti-neoplastic effects were observed when entinostat treatment was combined with gemcitabine or doxorubicin, while combination with the multi-kinase inhibitor sorafenib or the proteasome inhibitor bortezomib resulted in overadditive anti-neoplastic effects [41].

2.3.1.2 Rational for Use HDAC Inhibitors in PDAC

Whilst HDAC inhibitors have been investigated in numerous clinical trials with patients suffering from hematological cancers and other solid tumors, there are only few studies so far that tested them in PDAC patients.

In two phase I studies testing the safety and activity of entinostat given alone and in combination with 13-cis-retinoic acid (13-cR), one PDAC patient either was enrolled, achieving stable disease in the first setting, while progressive disease was noted after combined treatment [42].
In other phase I studies investigating the safety of oral treatment with mocetinostat and dacinostat respectively, PDAC patients had no objective tumor response.

In a phase I study tested the safety of combined treatment with belinostat plus carboplatin and/or paclitaxel, one PDAC patient of three showed partial remission after receiving belinostat and carboplatin. The same result was obtained in another phase I study investigating the combination of valproic acid with epirubicin.

A phase I study with 21 advanced PDAC patients investigated the maximally tolerated doses of concurrent vorinostat and capecitabine with radiation in neoadjuvant chemoradiation of

unresectable and borderline patients. The authors claimed potential activity of vorinostat in neoadjuvant chemoradiation to improve the intervention option in borderline patients.

The combinations of vorinostat with proteasome inhibitors showed some potential for the treatment of certain advanced solid tumors, thus it has investigating in PDAC. So far, all the study revealed no favorable responses in PDAC patients [43, 44].

Another phase I trial tested oral panobinostat combined with gemcitabine in advanced solid tumor patients, including 3 PDAC patients, of which 1 had stable disease under treatment [45]. Likewise, in a phase I study combining 9 PDAC-patients were treated, and five of them obtain stable disease [46].
A phase II study explored the activity of tacedinaline alone in 17 PDAC patients albeit well tolerated, no significant antitumoral activity was noted either in monotherapy or in combined treatment with gemcitabine [47].

2.4 Immunotherapy as an Anticancer Strategy in PDAC and CCA

Immunotherapeutic interventions have shown limited benefit to date in solid tumors that are not traditionally felt to be immunogenic (e.g., CCA and PDAC). This may relate to the presence of immunosuppressive factors which may be overcome through epigenetic modulation [48]. Indeed, the potential use of immune-based therapy to treat advanced CCA and PDAC has only recently been investigated.
The tumor microenvironment in PDAC is remarkable for its profound desmoplasia, absence of effector T cells and its T helper 2 (Th2) cell immunophenotype, which contribute to its ability to avoid immune surveillance [49, 50]. Many strategies to modulate the tumor microenvironment to a Th1 cell immunophenotype are under study: vaccination to induce or reinforce pre-existing immune responses is being investigated with agents such as GVAX (autologous pancreatic cell lines transfected with granulocyte–macrophage colony-stimulating factor) or CRS-207 (live attenuated Listeria monocytogenes-expressing mesothelin) alone or with a checkpoint inhibitor; directed cytolysis using T cells expressing chimeric antigen receptors; oncolytic virus therapy, to name but a few [51-54].

So far, treating PDAC with single-agent immune checkpoint inhibitors has not been effective [55, 56]. In prior studies, it was shown that membranous PD-L1 expression is scarce in PDACs [57], and since PD-L1 expression is shown to be activated in either by oncogenic signaling or by inflammatory cytokines, particularly interferon gamma, as a result of adaptive immune response, this low expression can be secondary to the lack in PDAC of effective T cell infiltration, as stated above [58, 59].

Evidence emerged recently that many genetic alterations in PDAC target epigenetic regulators [60, 61, 62]. In PDAC recent whole genomic sequencing revealed pathogenic mutations, structural variants and aberrant expression in several epigenetic regulator genes including *KDM6A*, *ARID1A*, *ARID1B*, *PBRM1*, *SMARCA2*, *SMARCA4*, and *MLL2* [31, 63]. Importantly, drug resistance in PDAC is mediated by pronounced plasticity enabling PDAC cells to switch between phenotypic states and to select for cellular clones that eventually evade therapy. The dynamic character of cell plasticity and drug resistance suggests the involvement of epigenetic regulation in controlling phenotypic heterogeneity in PDAC [64].
Nevertheless, recent translational research activities propose that combining modulation of the immune response and pharmacological targeting of epigenetic modifications might open highly powerful therapeutic avenues in the treatment of PDAC [65].

It has been demonstrated that PDL1 expression is present in a majority of CCA patients at baseline (49-94%), and is associated with poor survival, which suggests that CCA may be particularly sensitive to this strategy [66, 67]. In the study by Gani et al. the expression of PD-L1 within the tumor front was associated with an almost 60% worse survival [68]. These findings are very interesting insofar PD-L1 is an arguable biomarker for sensitivity to immune checkpoint inhibitors. Indeed, several clinical trials have investigated the potential role of immunotherapy in this malignance. Investigators at National Institute of Health (NIH) used a whole-exomic-sequencingbased approach to demonstrate that TILs from a patient with metastatic CCA contained CD4+Th1 cells recognizing a mutation in erbb2 interacting protein (*ERBB2IP*). After adoptive transfer of TILs containing mutation-specific polyfunctional TH1 cells, the patient achieved prolonged partial response. Upon disease progression, the patient was retreated with mutation-reactive TH1 cells and again experienced tumor regression [69].

Patients with MSI-H CCA who received pembrolizumab (an anti-PD-1) had a 71% objective response rate (ORR) and 67% progression free survival (PFS) [70]. Preliminary limited activity of immune checkpoint inhibitors in this disease is supported also by a phase 1B trial with pembrolizumab (Keynote 028), enrolling only patients with positive PD-L1 tumor expression, that reported a 17% ORR among CCA patients [71]. This result indicates that PD-1 inhibitors have modest but real activity in CCA and merit further investigation

Interestingly, exome sequencing in iCCA showed frequent mutations in genes involved in chromatin remodeling such as *BAP1*, *ARID1A*, and *PBRM1* [33]. Moreover, it has been established that *IDH1/2* mutations in CCA impair the activity of TET family of DNA dioxygenases resulting in a decrease of cytosine hydroxymethylation with a concurrent increase of DNA methylation [72]. The epigenetic alterations caused by IDH1/2 mutations lead to a blockade of cellular differentiation, causing an increase in the progenitor cells [73].

At the current time, there are no proven therapies for cancers having these mutations. In our opinion, HDAC inhibitors may offer therapeutic value and need further investigation.

All together these findings have reinforced the potential role of immune therapy and epigenetic approach in this disease, and they paved the way forward for investigating their appropriate combination in improving patient outcomes.

2.5 Investigational Agents

2.5.1 Entinostat (SNDX 275)

Entinostat belongs to the class of HDAC inhibitors which are critically important in the regulation of gene expression and in the field of target-specific anticancer drug development [34]. Entinostat is a member of the substituted pyridylcarbamate class of HDAC-inhibiting compounds with oral bioavailability [37, 38]. It selectively inhibits class I and IV HDACs, specifically, HDACs 1, 2, 3, and 11, thus promoting hyperacetylation of histones and allowing transcriptional activation of a distinct set of genes, ultimately inhibiting cell proliferation, terminal differentiation, and/or

apoptosis. This isoform selectivity may differentiate the safety and efficacy profiles from those of nonselective pan-HDAC inhibitors.

In addition, entinostat has demonstrated immunomodulatory activity that may translate to direct effects on the TME and enhanced antitumor immune responses [35, 36, 39].

2.5.2 Nonclinical Development of Entinostat

[redacted]

[redacted]

[redacted]

2.5.3 Clinical Development of Entinostat

[redacted]

[redacted]

[redacted]

[redacted]

2.5.4 Entinostat in Combination

[redacted]

[redacted]

2.5.5 Nivolumab (BMS-936558)

[redacted]

[redacted]

[redacted]

[redacted]

[redacted]

[redacted]

2.5.6 Nonclinical Development of Nivolumab

[redacted]

2.5.7 Clinical Development of Nivolumab

[redacted]

[redacted]

[redacted]

[redacted]

[redacted]

[redacted]

[redacted]

[redacted]

[redacted]

[redacted]

[redacted]

[redacted]

[redacted]

[redacted]

2.6 Entinostat in Combination with PD-1 Checkpoint Inhibition: Rational of the Study

One potential strategy for improving response to immune checkpoint inhibitors is to prime the tumor with an epigenetic therapy. Perhaps uniquely in the human body, the immune system is comprised of a complex network of interacting cells that is highly responsive and often stably adaptive to environmental cues.
Understandably, therefore, acknowledgments about the precise relationships between epigenetic aberrations, immune system and the consequences for cancer cell phenotypes could have tremendously important translational implications [95, 96]. Importantly, epigenetic changes may be reversible and thus represent an active and attractive area of new drug investigation [13, 27, 29].

[redacted]

[redacted]

[redacted]

[redacted]

[redacted]

[redacted]

[redacted]

[redacted]

[redacted]

[redacted]

[redacted]

[redacted]

[redacted]

[redacted]

[redacted]

[redacted]

[redacted]

[redacted]

[redacted]

[redacted]

[redacted]

[redacted]

[redacted]

[redacted]

[redacted]

[redacted]

[redacted]

[redacted]

3. PATIENT SELECTION

3.1 Eligibility Criteria

3.1.1. Patients must have histologically or cytologically confirmed cholangiocarcinoma or pancreatic adenocarcinoma that is metastatic or unresectable and has progressed despite standard therapy or is intolerant of therapy; 1 or 2 prior chemotherapy regimens in the metastatic setting or progression < 6 months from the completion of adjuvant chemotherapy.

3.1.2. Age ≥18 years.

3.1.3. ECOG performance status of 0 or 1 (Karnofsky ≥70%, see **Appendix A**).

3.1.4. Life expectancy of greater than 12 weeks.

3.1.5. Patients must have normal organ and marrow function as defined below:

- Hemoglobin (HgB) ≥ 9.0 g/dL
- Leukocytes ≥ 3,000/mcL
- Absolute neutrophil count ≥ 1,500/mcL
- Platelets ≥ 100,000/mcL
- Total bilirubin ≤ 1.5 x institutional upper limit of normal (ULN); an exception to this may be allowed for participants with Gilbert's syndrome with documented approval by the Protocol Chair
- AST(SGOT)/ALT (SGPT) ≤ 3 × institutional ULN
- Creatinine within normal institutional limits

or

- Creatinine clearance ≥ 60 mL/min using the Modified Cockcroft-Gault, as the formula below:

$$\text{Female CrCl} = \frac{(140 - \text{age in years}) \times \text{weight in kg} \times 0.85}{72 \times \text{serum creatinine in mg/dL}}$$

Male CrCl = (140 - age in years) x weight in kg x 1.00

72 x serum creatinine in mg/dL

3.1.6. Negative *(serum or urine)* pregnancy test, for women of childbearing potential. NOTE: If a patient has a positive or indeterminate serum or urine pregnancy test, then an ultrasound must be done to rule out pregnancy to enroll on trial. A serum B-HCG value of <25 is considered a negative pregnancy test.

3.1.7. Patients must have measurable disease per RECIST 1.1. (see **Section 8.1**)

3.1.8. Patient must have an accessible non-bone tumor lesion from which serial core biopsy specimens can be obtained.

NOTE: Patients with bone only disease are not eligible due to difficulties in obtaining serial bone biopsies for correlative analyses.

3.1.9. Women of child-bearing potential (WOCBP) and men must agree to use adequate contraception (hormonal or barrier method of birth control; abstinence) prior to study entry and during all the period of study participation for at least 5 months following the last dose of nivolumab (see **Section 4.7.2**). WOCBP must have a negative serum or urine pregnancy test (minimum sensitivity 25 IU/L or equivalent units of HCG) at baseline. Women must not be breastfeeding. Women who are not of childbearing potential (e.g., who are postmenopausal or surgically sterile as well as azoospermic men) do not require contraception. NOTE: If a patient has a positive or indeterminate serum or urine pregnancy test, then an ultrasound must be done to rule out pregnancy to enroll on trial. A serum B-HCG value of <25 is considered a negative pregnancy test.

3.1.10. Willingness to provide tissue and blood samples for mandatory translational research.

3.1.11. Ability to understand and the willingness to sign a written informed consent document.

3.2 Exclusion Criteria

3.2.1 Patients who have had chemotherapy or other systemic therapy or radiotherapy, or those who have not recovered from adverse events due to prior administered agents as follows:

- Chemotherapy < 3 weeks prior to registration
- Targeted therapy (other than below) < 2 weeks prior to registration
- Radiotherapy < 3 weeks prior to registration (**NOTE:** A previously irradiated lesion may not be used as a target lesion unless there is evidence of post-radiation progression.)
- Surgery < 3 weeks prior to registration
- Other approved or investigational agents <3 weeks prior to registration unless otherwise noted by the Protocol Chair.
- Patients who have received prior epigenetic therapy (e.g. HDAC inhibitors such as entinostat, panobinostat, vorinostat, romidepsin or demethylating agents such as 5azacitidine or decitabine)
- Those who have not recovered from acute adverse events to grade <2 or baseline due to agents administered, with exception of alopecia or stable neuropathy, unless approved by the Protocol Chair.

3.2.2 Patients with a history of prior treatment with anti-PD-1, anti-PD-L1, anti-PD-L2, antiCTLA4 antibodies or any other antibody or drug specifically targeting T-cell costimulation or immune checkpoint pathways.

3.2.3 Known sensitivity to or history of allergic reactions attributed to compounds of similar chemical or biologic composition of entinostat and/or nivolumab. History of severe hypersensitivity reaction to any monoclonal antibody.

3.2.4 Patients with active autoimmune disease or history of autoimmune disease that might recur, which may affect vital organ function or require immune suppressive treatment including systemic corticosteroids, should be excluded. These include but are not limited to patients with a history of immune related neurologic disease, multiple sclerosis, autoimmune (demyelinating) neuropathy, Guillain-Barre syndrome, myasthenia gravis; systemic autoimmune disease such as SLE, connective tissue diseases, scleroderma, inflammatory bowel disease (IBD), Crohn's, ulcerative colitis, autoimmune hepatitis; and patients with a history of toxic epidermal necrolysis (TEN), Stevens-Johnson syndrome, or phospholipid syndrome should be excluded because of the risk of recurrence or exacerbation of disease. Patients with vitiligo, endocrine deficiencies including thyroiditis managed with replacement hormones including physiologic

corticosteroids are eligible. Patients with rheumatoid arthritis and other arthropathies, Sjögren's syndrome and psoriasis controlled with topical medication and patients with positive serology, such as antinuclear antibodies (ANA), anti-thyroid antibodies should be evaluated for the presence of target organ involvement and potential need for systemic treatment but should otherwise be eligible.

NOTE: Patients are permitted to enroll if they have vitiligo, type I diabetes mellitus, residual hypothyroidism due to autoimmune condition only requiring hormone replacement, psoriasis not requiring systemic treatment, or conditions not expected to recur in the absence of an external trigger (precipitating event).

3.2.5 Subjects with interstitial lung disease that is symptomatic or may interfere with the detection or management of suspected drug-related pulmonary toxicity.

3.2.6 Patient has a pulse oximetry of <92% on room air or is on supplemental home oxygen.

3.2.7 Patients with active or untreated brain metastases or leptomeningeal metastases are excluded from this clinical trial because of their poor prognosis and because they often develop progressive neurologic dysfunction that would confound the evaluation of neurologic and other adverse events.

NOTE: Patients with previously treated brain metastases must have stable neurologic status and imaging following local therapy (surgery or radiation) for at least 4 weeks, with no requirement for immunosuppressive doses of systemic corticosteroids (>10 mg/day prednisone equivalents) for at least 2 weeks prior to study drug administration (stable low dose dexamethasone allowed at discretion of IND Sponsor).

3.2.8 Uncontrolled intercurrent illness including, but not limited to, ongoing or active infection, symptomatic congestive heart failure, unstable angina pectoris, cardiac arrhythmia, or psychiatric illness/social situations that in the judgment of the investigator would limit compliance with study requirements.

3.2.9 Pregnant women are excluded from this study because the agents have the potential for teratogenic or abortifacient effects. Because there is an unknown but potential risk for

adverse events in nursing infants secondary to treatment of the mother, breastfeeding should be discontinued.

3.2.10 Known HIV-positive patients on combination antiretroviral therapy are ineligible. Patients who have a positive test for hepatitis B virus surface antigen (HBV sAg) or hepatitis C antibody (HCV Ab)/RNA (HCV RNA) indicating acute or chronic infection are also ineligible (baseline testing required).

3.2.11 Patients should be excluded if they have a condition requiring systemic treatment with either corticosteroids (>10 mg daily prednisone equivalents) or other immunosuppressive medications within 14 days of study drug administration. Inhaled or topical steroids and adrenal replacement doses ≤10 mg daily prednisone equivalents are permitted in the absence of active autoimmune disease. Patients are permitted to use topical, ocular, intraarticular, intranasal, and inhalational corticosteroids (with minimal systemic absorption). Physiologic replacement doses of systemic corticosteroids are permitted, even if ≤10 mg/day prednisone equivalents. A brief course of corticosteroids for prophylaxis (e.g., contrast dye allergy) or for treatment of non-autoimmune conditions (e.g., delayed-type hypersensitivity reaction caused by contact allergen) is permitted.

3.2.12 Patients requiring concurrent administration of valproic acid are also excluded.

3.2.13 Patients who have had evidence of active or acute diverticulitis, intra-abdominal abscess, or GI obstruction which are known risk factors for bowel perforation should be evaluated for the potential need for additional treatment before coming on study.

3.2.14 Any contraindication to oral agents or significant nausea and vomiting, malabsorption, or significant small bowel resection that, in the opinion of the investigator, would preclude adequate absorption.

3.2.15 Another active malignancy ≤ 3 years prior to registration with the exception of nonmelanotic skin cancer or carcinoma-in-situ of any type. NOTE: If there is a history of prior malignancy, they must not be receiving other specific treatment for their cancer. Any malignancy considered to be indolent and that has never required therapy may allowed at the discretion of the IND Sponsor.

3.2.16 Patient is unwilling or unable to follow the study schedule for any reason.

3.2.17 Patient is, at the time of signing informed consent, a regular user (including "recreational use") of any illicit drugs or other substance abuse (including alcohol) that could potentially interfere with adherence to study procedures or requirements.

3.2.18 Evidence of clinical or radiographic ascites. Trace or small amounts of radiographic ascites without prior concern for malignant ascites or not associated with peritoneal carcinomatosis may be approved by the protocol chair. NOTE: 3-D CT scans and RECIST reads will not be used to determine eligibility at baseline.

3.3 Inclusion of Women and Minorities

NIH policy requires that women and members of minority groups and their subpopulations be included in all NIH-supported biomedical and behavioral research projects involving NIH-defined clinical research unless a clear and compelling rationale and justification establishes to the satisfaction of the funding Institute & Center (IC) Director that inclusion is inappropriate with respect to the health of the subjects or the purpose of the research. Exclusion under other circumstances must be designated by the Director, NIH, upon the recommendation of an IC Director based on a compelling rationale and justification. Cost is not an acceptable reason for exclusion except when the study would duplicate data from other sources. WOCBP should not be routinely excluded from participation in clinical research.

4. TREATMENT PLAN

4.1 Agents Administration

Treatment will be administered on an outpatient basis. Reported adverse events and potential risks are described in **Section 6**. Appropriate dose modifications are described in **Section 5**.

No investigational or commercial agents or therapies other than those described below may be administered with the intent to treat the patient's malignancy.

The cycle length is 28 days. Treatment will consist of the combination of entinostat and nivolumab. A lead-in of entinostat 2 weeks prior to combination therapy at the dose of 5 mg/weekly, will be incorporated to help determine the effect of entinostat on immune-related parameters in tumor biopsies.

4.2 Overall Study Design

The proposed study single institution, open-label, two-stage, phase 2 study evaluating the clinical activity and safety of entinostat plus nivolumab. We will enroll two cohorts of patients: unresectable or metastatic cholangiocarcinoma (Cohort 1) and unresectable or metastatic pancreatic adenocarcinoma (Cohort 2). The primary objective of the trial is to determine whether the combination of entinostat plus nivolumab yields a clinically compelling antitumor activity measured as objective response rate (ORR, assessed by RECIST 1.1).

Secondary endpoints include safety, progression-free survival (PFS), overall survival (OS), and immunologic correlates.

The study is planned with 27 evaluable subjects per histology based on a two-stage design that allows early termination for lack of efficacy.

Patients will receive a biopsy at baseline and on approximately day 14 of entinostat therapy and approximately after 6 weeks from Cycle 1 Day 1. An optional biopsy will be performed at the time of disease progression. After a fourteen-day lead in with entinostat monotherapy, patients will begin to concurrently receive entinostat 5 mg oral once a week plus nivolumab 240 mg every two weeks for approximately four months. After 4 months, nivolumab will be given at a dose of 480 mg fixed dose every 4 weeks (maintenance phase). Therapy may continue with entinostat 5 mg weekly plus nivolumab 480 mg every 4 weeks, until intolerance or progression. Nivolumab will be administered intravenously over a period of 30 minutes.

4.3 Definition of Unacceptable Toxicities

Unacceptable toxicities are defined as treatment-related:

- Grade 3 or greater non-hematologic toxicities. Exceptions include:

- Grade 3 nausea/vomiting if improved to Grade 2 levels or better within 72 hours
- Asymptomatic laboratory abnormalities
- Grade 3 fatigue
- Grade 3 dermatologic AEs that are considered mild in severity but only considered grade 3 because of >30% body surface involvement
- Grade 3 pruritus
- Grade 3-4 hyperglycemia or grade 3 endocrinopathies where symptoms are controlled on hormone replacement therapy

• Febrile neutropenia Grade 3 or Grade 4:
  - Grade 3 is defined as ANC <1.0×109/L with a single temperature of >38.3°C (101°F) or a sustained temperature of ≥38°C (100.4°F) for more than 1 hour
  - Grade 4 is defined as ANC <1.0×109/L with a single temperature of >38.3°C (101°F) or a sustained temperature of ≥38°C (100.4°F) for more than 1 hour, with life-threatening consequences and urgent intervention indicated.

• Grade 4 thrombocytopenia (i.e., platelet count <25×109/L)
• Grade 3 thrombocytopenia with clinically significant bleeding
• Any other toxicity resulting in a dose reduction in therapy (at the discretion of the IND Sponsor)
• Failure to complete all protocol specified treatment doses (at the discretion of the IND Sponsor) • Concurrent elevation of transaminases and bilirubin consistent with Hy's Law criteria

Note: The original Hy's Law parameters for treated patients relative to comparator patients have been broadened over time (Kaplowitz and DeLeve 2003, Zimmerman 1999) to include additional parameters, but still require that these 3 criteria be met:

i. injury: elevation of >3×ULN ALT or AST activity; and ii.function: >2×ULN total bilirubin (another clinical marker for function, such as >1.5×ULN INR may be acceptable if the change is clinically significant in the absence of obstruction) without >2×ULN alkaline phosphatase (ALP); and iii.clinical verification to ensure effect is health product-induced and not induced by disease or another cause of injury.

• Grade 5 toxicity

4.4 Study Drugs

NOTE: Subjects will be given an information sheet and wallet card to remind them of serious risks and that may be shared with their other healthcare providers, including a reminder to notify

us of all new medications started during the course of the study due to the possibility for drug interactions (**Appendix B**).

4.4.1 Entinostat (MS-275, SNDX-275)

[redacted]

[redacted]

[redacted]

4.3.1 Nivolumab (OPDIVO ®, BMS-936558; MDX-1106)

[redacted]

[redacted]

[redacted]

[redacted]

[redacted]

4.5 Supportive Care Guidelines and General Concomitant Medication

4.5.1 Supportive Care Guidelines

In general, concomitant medications and therapies deemed necessary for the supportive care and safety of the subject are allowed, provided their use is documented in the medical records and meets the dose modification guidelines outlined in **Section 5**.

The administration of any other therapies intended to treat the primary condition including chemotherapy and biologic agents is NOT permitted. Similarly, the use of other concurrent investigational drugs is not allowed.

Patients should receive appropriate supportive care measures as deemed necessary by the Investigator.

Blocking PD-1 function may permit the emergence of auto-reactive T cells and resultant clinical autoimmunity. Rash/pruritus, diarrhea/colitis, pneumonitis, hepatitis, and hypothyroidism were drug-related, presumptive autoimmune events noted in previous nivolumab studies.

All adverse events (AE) previously described in the Investigator's Brochures (IB) will be considered nivolumab-related AEs. For the purposes of this study, AE of unknown etiology, associated with drug exposure and consistent with an immune phenomenon, will be also considered nivolumab-related AE. Efforts should be made to rule out neoplastic, infectious, metabolic, toxin or other etiologic causes. Serological, immunological, and histological (biopsy) data should be used to support the diagnosis of an immune-mediated toxicity. Suspected nivolumab -related AEs must be documented on an AE or SAE CRF. Identification and treatment of nivolumab-related AEs can be found in **Appendix D**. Additional guidance can be found in the nivolumab IB.

Subjects who experience a grade 2 or higher nivolumab or entinostat-related AE should be discussed with the Principal Investigator (PI) and IND sponsor immediately

4.5.2 Hypersensitivity/Infusion Reactions

4.5.2.1 Entinostat

Hypersensitivity reactions should be managed as per standard of care at the treating institution. Severe reactions, such as hypotension requiring treatment, dyspnea requiring bronchodilators, angioedema or generalized urticaria require immediate discontinuation of study drug administration and aggressive symptomatic therapy.
Subjects who experience a severe hypersensitivity reaction to treatment should not be rechallenged.

4.5.2.2 Nivolumab

Nivolumab is a fully human monoclonal immunoglobulin (Ig) G4 antibodies. Subjects should be closely monitored for potential AEs during antibody infusion and potential AEs throughout the study.

Since nivolumab contains only human immunoglobulin protein sequences, it is unlikely to be immunogenic and induce an infusion or hypersensitivity reaction. However, if such a reaction were to occur, it might manifest with fever, chills, rigors, headache, rash, pruritus, arthralgia, hypo- or hypertension, bronchospasm, or other symptoms. All grade 3 or 4 infusion reactions should be reported within 24 hours to the IND Sponsor and BMS and Syndax as an SAE if the criteria are met. Infusion reactions should be graded according to CTCAE (version 4.03) guidelines. Treatment recommendations are provided below and may be modified based on local treatment standards and guidelines as appropriate:

- For Grade 1 symptoms: (Mild reaction; infusion interruption not indicated; intervention not indicated).
  Remain at bedside and monitor subject until recovery from symptoms. The following prophylactic premedications are recommended for future infusions: diphenhydramine 50 mg (or equivalent) and/or paracetamol 325 to 1000 mg (acetaminophen) at least 30 minutes before additional nivolumab administrations.

- For Grade 2 symptoms: (Moderate reaction requires therapy or infusion interruption but responds promptly to symptomatic treatment [e.g, antihistamines, non-steroidal antiinflammatory drugs, narcotics, corticosteroids, bronchodilators, IV fluids]; prophylactic medications indicated for 24 hours).

Stop the nivolumab infusion, begin an IV infusion of normal saline, and treat the subject with diphenhydramine 50 mg IV (or equivalent) and/or paracetamol 325 to 1000 mg (acetaminophen); remain at bedside and monitor subject until resolution of symptoms. Corticosteroid or bronchodilator therapy may also be administered as appropriate. If the infusion is interrupted, then restart the infusion at 50% of the original infusion rate when symptoms resolve; if no further complications ensue after 30 minutes, the rate may be increased to 100% of the original infusion rate. Monitor subject closely. If symptoms recur then no further nivolumab will be administered at that visit. Administer diphenhydramine 50 mg IV, and remain at bedside and monitor the subject until resolution of symptoms. The amount of study drug infused must be recorded on the electronic case report form (eCRF). The following prophylactic premedications are recommended for future infusions: diphenhydramine 50 mg (or equivalent) and/or paracetamol 325 to 1000 mg (acetaminophen) should be administered at least 30 minutes before additional nivolumab administrations. If necessary, corticosteroids (recommended dose: up to 25 mg of IV hydrocortisone or equivalent) may be used.

- For Grade 3 or Grade 4 symptoms: (Severe reaction, Grade 3: prolonged [e.g., not rapidly responsive to symptomatic medication and/or brief interruption of infusion]; recurrence of symptoms following initial improvement; hospitalization indicated for other clinical sequelae [eg, renal impairment, pulmonary infiltrates]). Grade 4: (life threatening; pressor or ventilatory support indicated).

  Immediately discontinue infusion of nivolumab. Begin an IV infusion of normal saline, and treat the subject as follows. Recommend bronchodilators, epinephrine 0.2 to 1 mg of a 1:1,000 solution for subcutaneous administration or 0.1 to 0.25 mg of a 1:10,000 solution injected slowly for IV administration, and/or diphenhydramine 50 mg IV with methylprednisolone 100 mg IV (or equivalent), as needed. Subject should be monitored until the investigator is comfortable that the symptoms will not recur.

  Nivolumab will be permanently discontinued. Investigators should follow their institutional guidelines for the treatment of anaphylaxis. Remain at bedside and monitor subject until recovery from symptoms. In the case of late-occurring hypersensitivity symptoms (eg, appearance of a localized or generalized pruritus within 1 week after treatment), symptomatic treatment may be given (eg, oral antihistamine, or corticosteroids).

In the case of late-occurring hypersensitivity symptoms (e.g., appearance of a localized or generalized pruritus within 1 week after treatment), symptomatic treatment may be given (e.g., oral antihistamine, or corticosteroids). Additional treatment prior to next dose as per guidelines above.

Please note that late occurring events including isolated fever and fatigue may represent the presentation of systemic inflammation. Please evaluate accordingly.

Nivolumab-related infusion reactions and adverse events must resolve prior to administration of entinostat.

4.5.3 Prohibited and Restricted Therapies

Patients may not use any of the following agents during the study:

- Any non-study anticancer or immunotherapy agent (investigational or non-investigational). **NOTE**: With approval of the IND Sponsor subjects may receive palliative radiation therapy as long as a site(s) disease outside of the radiation field is available to follow for response. No study drug should be given during radiation therapy.

- Concomitant use of valproic acid is prohibited due to its known activity as a HDAC inhibitor.

- CD137 or other immunologic activation agonists.

- Immunosuppressive agents.

- Chronic systemic corticosteroids at supraphysiologic doses.

NOTE: Subjects are permitted to use topical, ocular, intra-articular, intranasal, and inhalational corticosteroids (with minimal systemic absorption).
Physiologic replacement doses of systemic corticosteroids are permitted, in the absence of active autoimmune disease.

A brief course of corticosteroids for prophylaxis (e.g., contrast dye allergy), for treatment of non-autoimmune conditions (e.g., delayed-type hypersensitivity reaction caused by contact

allergen), and for treatment of infusion reactions and premedication (e.g., up to 25 mg IV hydrocortisone) is permitted.

Otherwise, steroid treatment should be completed at least 14 days prior to resuming studyrelated treatments unless approval to resume sooner is obtained from the IND Sponsor.

- Live vaccines (examples of live vaccines include, but are not limited to: measles, mumps, rubella, chicken pox, yellow fever, rabies, BCG, and typhoid [oral] vaccine). Seasonal influenza vaccines for injection are generally killed virus vaccines and are allowed. However, intranasal influenza vaccines (e.g. Flu-Mist®) are live attenuated vaccines, and are NOT allowed.

- The use of anticoagulants is known to increase the risk of gastrointestinal hemorrhage. Since gastrointestinal hemorrhage is an adverse reaction with nivolumab, subjects who require concomitant anticoagulant therapy should be monitored closely.

- Concomitant use of the drugs below with entinostat should be avoided during the study (also listed in **Appendix E**):
  - Sensitive substrates of CYP1A2, CYP2C8, CYP3A with a narrow therapeutic window
  - Drugs that are known to inhibit or induce P-gp

4.5.4 Precautions

Combination therapy may result in unexpected toxicity especially in novel combinations with other immune modifying agents. A striking example in macaques is presented in Vaccari, et al. 2012 [120].

4.6 Definition of an Overdose for this Protocol

An overdose is defined as the accidental or intentional administration of any dose of a product that is considered both excessive and medically important. All occurrences of overdose must be reported as SAEs (see **Section 6.6**).

4.7 WOCBP, Contraception, Use in Pregnancy, Use in Nursing

4.7.1 WOCBP

A WOCBP is defined as any female who has experienced menarche and who has not undergone surgical sterilization (hysterectomy or bilateral oophorectomy) and is not postmenopausal. Menopause is defined as 12 months of amenorrhea in a woman over age 45 years in the absence of other biological or physiological causes. In addition, women under the age of 55 years must have a serum follicle stimulating hormone (FSH) level > 40mIU/mL to confirm menopause. Women treated with hormone replacement therapy (HRT) are likely to have artificially suppressed FSH levels and may require a washout period in order to obtain a physiologic FSH level. The duration of the washout period is a function of the type of HRT used. The duration of the washout periods below are suggested guidelines and the investigators should use their judgment in checking serum FSH levels. If the serum FSH level is >40 mIU/ml at any time during the washout period, the woman can be considered postmenopausal:

- 1 week minimum for vaginal hormonal products (rings, creams, gels)
- 4 week minimum for transdermal products
- 8 week minimum for oral products

4.7.2 Contraception

The investigational agents used in this protocol may have adverse effects on a fetus in utero. Furthermore, it is not known if the investigational agents have transient adverse effects on the composition of sperm. Non-pregnant, non-breast-feeding women may be enrolled if they are considered highly unlikely to conceive. Highly unlikely to conceive is defined as 1) surgically sterilized, or 2) postmenopausal, or 3) amenorrheaic for <2 years without a hysterectomy and oophorectomy and with a documented FSH value in the postmenopausal range, or 4) not heterosexually active for the duration of the study, or 5) heterosexually active and willing to use 2 methods of birth control (which is also required for the female partners of male subjects). The 2 birth control methods can be 2 barrier methods or a barrier method plus a hormonal method to prevent pregnancy, used throughout the study starting with Visit 1 through 5 months after the last

dose of study medication. Male subjects enrolled in this study must also agree to use an adequate method of contraception starting with Visit 1 through 7 months after the last dose of study drug.

Investigators shall counsel WOCBP and male subjects who are sexually active with WOCBP on the importance of pregnancy prevention and the implications of an unexpected pregnancy. Investigators shall advise WOCBP and male subjects who are sexually active with WOCBP on the use of highly effective methods of contraception. Highly effective methods of contraception have a failure rate of < 1% per year when used consistently and correctly.

4.7.2.1 Highly Effective Methods of Contraception

- Male condoms with spermicide.
- Hormonal methods of contraception including combined oral contraceptive pills, vaginal ring, injectables, implants, and intrauterine devices (IUDs) such as Mirena by WOCBP subject or male subject's WOCBP partner.
- Nonhormonal IUDs, such as ParaGard.
- Tubal ligation.
- Vasectomy.
- Complete abstinence.

Complete abstinence is defined as complete avoidance of heterosexual intercourse and is an acceptable form of contraception for all study drugs. Abstinence is only acceptable when this is in line with the preferred and usual lifestyle of the subject. Periodic abstinence (e.g., calendar, ovulation, symptothermal, profession of abstinence for entry into a clinical trial, post-ovulation methods) and withdrawal are not acceptable methods of contraception. Subjects who choose complete abstinence are not required to use a second method of contraception, but female subjects must continue to have pregnancy tests. Acceptable alternate methods of highly effective contraception must be discussed in the event that the subject chooses to forego complete abstinence. NOTE: If a patient has a positive or indeterminate serum or urine pregnancy test, then

an ultrasound must be done to rule out pregnancy to enroll on trial. A serum B-HCG value of <25 is considered a negative pregnancy test.

Subjects should be informed that taking the study medication may involve unknown risks to the fetus (unborn baby) if pregnancy were to occur during the study. In order to participate in the study they must adhere to the contraception requirement (described above) for the duration of the study. If there is any question that a subject will not reliably comply with the requirements for contraception, that subject should not be entered into the study.

4.7.3 Use in Pregnancy

The investigational agents used in this protocol may have adverse effects on a fetus; therefore, women with a positive pregnancy test at screening will not be eligible for enrollment. If a subject inadvertently becomes pregnant while on treatment, the subject will immediately be removed from the study treatment. The study team will contact the subject at least monthly and document the subject's status until the pregnancy has been completed or terminated.

The investigator must immediately notify the Syndax Sponsor and BMS of any pregnancy using the Pregnancy Surveillance Form within 24 hours of notification and in accordance with the SAE reporting procedures described in Section 6.5. Any pregnancy that occurs in a female partner of a male study participant should also be reported to the Syndax and BMS.

Protocol required procedures for study treatment discontinuation and follow-up must be performed on the subject unless contraindicated by pregnancy (e.g. x-ray studies). Other appropriate pregnancy follow-up procedures should be considered if indicated.

Follow-up information regarding the course of the pregnancy, including perinatal and neonatal outcome and where applicable, offspring information must be reported on the Pregnancy Surveillance Form.

4.7.4 Use in Nursing Women

Since many drugs are excreted in human milk, and because of the potential for serious adverse reactions in the nursing infant, subjects who are breast-feeding are not eligible for enrollment.

4.8 Duration of Treatment and Criteria for Removal from Treatment

In general, treatment will continue until one of the following criteria are met:

- Disease progression. **NOTE**: Treatment may continue under the guidelines discussed in **Section 4.9**, as per opinion of the Investigators.

- The patient or legal representative (such as a parent or legal guardian) withdraws consent for treatment but not follow up.

- Intercurrent illness that prevents further administration of treatment.

- Unacceptable adverse events. If the study treatment has provided clinical benefit, the IND sponsor may approve trial continuation for patients experiencing entinostat-related toxicities that are not life threatening or of major clinical concern (such as pruritis or rash). **NOTE**: Assessment for discontinuation of entinostat may be made independently of nivolumab, continuation of entinostat may be considered with approval by the IND sponsor. There are instances where treatment with nivolumab may continue if entinostat has been discontinued. (**Section 5.1)**

- If in the opinion of the Investigator, a change or temporal or permanent discontinuation of therapy would be in the best interest of the patient. The Sponsor should be included in this decision.

- Noncompliance with trial treatment or procedure requirements.

- Patient becomes pregnant. All women of child bearing potential should be instructed to contact the investigator immediately if they suspect they might be pregnant (e.g., missed or late menstrual period) at any time during study participation. The investigator must immediately notify CTEP in the event of a confirmed pregnancy in a subject participating in the study.

- Treatment required with other chemotherapeutic or investigational anti-neoplastic drugs.

- Patient is lost to follow-up.

- In the case that a participant develops intolerance or side effect that is attributable to entinostat, the participant may, at his or her discretion, continue to receive only Nivolumab as otherwise indicated as long as they have received at least 1 full dose of both entinostat and nivolumab and nivolumab re-treatment criteria are met as outlined above.

- If nivolumab has to be permanently discontinued because drug-related toxicity, the patient will be permanently discontinued from entinostat treatment too.

The subject must be discontinued from the trial for any of the following reasons:

- The patient or legal representative (such as a parent or legal guardian) withdraws consent for follow-up.

- Termination of the study.

- Patient is lost to follow up.

The reason for study removal and the date the subject was removed must be documented in the Case Report Form.

4.9 Treatment Beyond Progression

A minority of subjects treated with immunotherapy may derive clinical benefit either delayed responses, stable disease, or increased overall survival despite initial evidence of progressive disease (PD) with nivolumab or combination treatment.

Subjects may be permitted to continue treatment beyond initial RECIST 1.1-defined PD as long as the following criteria are met:

- Patients must be clinically stable with no change in performance status due to disease progression.

- No indication for immediate alternative treatment

- Patient [assessed by the investigator] is showing clinical benefit and tolerates study drug. The assessment of clinical benefit should take into account whether the subject is clinically stable or deteriorating and likely or unlikely to receive further benefit from continued treatment

- The time of progression is noted from the first assessment that exceeds standard criteria All decisions to continue treatment beyond initial progression must be discussed with the PI in advance, including if for any exception to the criteria noted above.

New lesions are considered measurable at the time of initial progression if the longest diameter is at least 10 mm (except for pathological lymph nodes, which must have a short axis of at least 15 mm). Any new lesion considered non-measurable at the time of initial progression may become measurable and therefore included in the tumor burden measurement if the longest diameter increases to at least 10 mm (except for pathological lymph nodes, which must have an increase in short axis to at least 15 mm).

For statistical analyses that include the investigator-assessed progression date, subjects who continue treatment beyond initial investigator-assessed, RECIST 1.1-defined progression will be considered to have investigator-assessed progressive disease at the time of the initial progression event. Subjects who have tumor shrinkage following RECIST-defined progression (e.g., irRC) will be also summarized separately.

4.10 Duration of Follow Up

The Follow-Up Phase begins when the decision to discontinue a subject from study therapy is made (no further treatment with study therapy).

All subjects who discontinue investigational product should comply with protocol specified follow-up procedures as outlined in Section 10, Study Calendar.

A mandatory End of treatment (EOT) visit (+/- 7 days) should be performed 28 days after the last dose of study drug(s).

After completion of the EOT visit, subjects will be followed every 3 months for survival.

Subjects who are discontinued from the study treatment due to an unacceptable toxicity will be monitored for safety until the resolution of the AE to ≤ grade 1 or stabilization or until initiation of a new therapy for their cancer, whichever occurs first.

SAEs will be collected and recorded throughout the study period, from the first dose of the investigational agent through 100 days (+ 14 days) after the last dose of study drug or end of the study (if thought to be related to study drug), or 30 days after the initiation of a new anticancer therapy, whichever is earlier.

5. DOSING DELAYS/DOSE MODIFICATIONS

In order to maintain dose-intensity and cumulative dose-delivery on this study, reasonable efforts will be made to minimize dose reduction and treatment delays as specified below.
Although entinostat and immune checkpoint blockade have distinct toxicity profiles, they do share some adverse events such as fatigue, nausea and diarrhea. There is the theoretical possibility that one agent may potentiate the other and hence drug causality will not always be clear. In the event of uncertainty, dose reductions and/or delays will follow the most conservative approach.

For any event which is apparent at baseline, the dose modifications will apply according to the corresponding shift in toxicity grade, if the investigator feels it is appropriate (e.g., if a subject has grade 1 asthenia at baseline which increases to grade 2 during treatment, this will be considered as a shift of 1 grade and treated as a grade 1 toxicity for dose modification purposes).

All toxicity grades below are described using the NCI Common Terminology Criteria for Adverse Events (CTCAE) version 4.03.

All discontinuations, relationships to treatment, and treatment decisions must be clearly documented in the medical record. Any questions should be discussed with the IND Sponsor.

Dose reductions due to toxicity are permanent. Dose escalation is not allowed. Dose reduction is not allowed for Nivolumab treatment.

NOTE: Tumor assessments should continue as per protocol even if dosing is delayed.

5.1 Dose Criteria

Entinostat

Prior to administration of first dose of entinostat in each cycle, subject's organ function and treatment-related toxicities must have recovered to the following values:

- Absolute Neutrophil Count (ANC) ≥ 1,000 x 106/L
- Platelets ≥ 75,000 x 106/L
- Creatinine ≤ 1.5 x ULN OR > 60 mL/min for patient with creatinine levels >1.5× institutional ULN.
- Non-hematologic toxicities ≤ Grade 2 as noted below for entinostat-related toxicity **Nivolumab**

Dosing of nivolumab will be delayed for any of the criteria listed below:

- Total bilirubin ≥ 1.5×ULN (for patients with diagnosed Gilbert's Syndrome, direct bilirubin should be within normal institutional limits).
- Aspartate aminotransferase (AST) and alanine aminotransferase (ALT) > 3 ×ULN **OR** ≥5 ×ULN for patients with liver metastases: NOTE - appropriate evaluation for autoimmune transaminitis must be conducted
- Any ≥ Grade 2 non-skin, non-laboratory, drug-related adverse event, with the following exception:
- Grade 2 drug-related fatigue does not require a treatment delay.
- Grade 2 hypothyroidism or thyroiditis does not require a treatment delay

- Subjects with Grade 3 thyroiditis or hypopituitarism may be restarted with replacement hormones including thyroid hormone and physiologic doses of corticosteroids.

  NOTE: grading for hypophysitis with symptoms of headache, visual or neurologic changes or radiologic evidence of pituitary enlargement and other CNS events such as aseptic meningitis or encephalitis should be considered grade 3 events

- Any ≥ Grade 3 skin, drug-related adverse event.

- Any ≥ Grade 3 drug-related laboratory abnormality with the following exceptions:

- Grade 3 lymphopenia does not require dose delay

- Any Grade ≥ 3 drug-related amylase or lipase abnormality that is not associated with symptoms or clinical manifestations of pancreatitis does not require dose delay.

- Isolated grade 3 or 4 electrolyte imbalances/abnormalities that are not associated

with clinical sequelae and are corrected with supplementation/appropriate management.

- Any adverse event, laboratory abnormality, or intercurrent illness which, in the judgment of the investigator, warrants delaying the dose of study medication

If the criteria to resume treatment are met, the patient should restart treatment no sooner than the next scheduled time point per protocol. However, if the treatment is delayed past the next scheduled time point per protocol the treatment should resume at the earliest convenient point that is within the six week delay period.

5.1.1 Entinostat Dose Adjustments

[redacted]

[redacted]	[redacted]
[redacted]	[redacted]

[redacted]	[redacted]
[redacted]	[redacted]
[redacted]	[redacted]
[redacted]	[redacted]

[redacted]

[redacted]

[redacted]

[redacted]	[redacted]
[redacted]	[redacted]
[redacted]	[redacted]

[redacted]

5.1.2 Discontinuation of Entinostat Treatment

Entinostat treatment should be discontinued if any of the following criteria are met:
• For any grade 4 toxicity that recurs despite prophylaxis or dose reduction

• Any adverse event, laboratory abnormality, or intercurrent illness which, in the judgment of the investigator, presents a substantial clinical risk to the subject with continued study drug dosing

5.1.3 Nivolumab Dose Managements

[redacted]

[redacted]	[redacted]
[redacted]	[redacted]
[redacted]	[redacted]
[redacted]	[redacted]

[redacted]	[redacted]
[redacted]	[redacted]
[redacted]<br><br>[redacted]	[redacted]
[redacted]<br><br>[redacted]	[redacted]

[redacted]	[redacted]
[redacted]	[redacted]
[redacted]	[redacted]

[redacted]	[redacted]
[redacted]	[redacted]
[redacted]	[redacted]
[redacted]	

[redacted]	[redacted]
[redacted]	[redacted]
[redacted]	[redacted]
[redacted]	[redacted]

[redacted]	[redacted]
[redacted]	[redacted]
[redacted]	[redacted]
[redacted]	[redacted]
[redacted]	[redacted]

[redacted]	[redacted]
[redacted]	[redacted]
[redacted]	[redacted]
[redacted]	[redacted]

[redacted]	[redacted]
[redacted]	[redacted]
[redacted]	[redacted]
[redacted]	[redacted]

[redacted]	[redacted]
[redacted]	[redacted]
[redacted]	[redacted]
[redacted]	[redacted]

[redacted]	[redacted]
[redacted]	[redacted]
[redacted]	[redacted]
[redacted]	[redacted]

[redacted]	[redacted]
[redacted]	[redacted]
[redacted]	[redacted]
[redacted]	[redacted]

[redacted]	[redacted]
[redacted]	[redacted]
[redacted]	[redacted]
[redacted]	[redacted]

[redacted]	[redacted]
[redacted]	[redacted]
[redacted]	[redacted]
[redacted]	[redacted]

[redacted]	[redacted]
[redacted]	[redacted]
[redacted]	
[redacted]	[redacted]
[redacted]	

5.1.4 Nivolumab Treatment Discontinuation

Nivolumab treatment should be discontinued if any of the following criteria are met:

- Any Grade 2 treatment-related uveitis, eye pain, or blurred vision that does not respond to topical therapy and does not improve to Grade 1 severity within 2 weeks of starting therapy requires systemic treatment

- Any Grade 3 non-skin, drug-related AE lasting > 7 days, with the following exceptions:
  - Grade 3 treatment-related uveitis, pneumonitis, bronchospasm, diarrhea, colitis, neurologic toxicity, hypersensitivity reaction, or infusion reaction of any duration requires discontinuation
  - Grade 3 treatment-related endocrinopathies (except for adrenal insufficiency) adequately controlled with only physiologic hormone replacement DO NOT require discontinuation
  - Grade 3 fatigue that lasts >14 days

- Grade 3 treatment-related thrombocytopenia > 7 days OR that is associated with bleeding requires discontinuation

- Any treatment-related liver function test (LFT) abnormality that meets the following criteria, require discontinuation:
  - Total bilirubin > 5 × ULN
  - Concurrent AST or ALT ≥ 3 × ULN and total bilirubin > 2 × ULN

- Any treatment-related liver function test (LFT) abnormality that meets the following criteria, require discontinuation:
  - AST or ALT >5 x ULN in patients with AST/ALT level within normal limits at baseline
  - AST or ALT >10 × ULN in patients with AST/ALT level more than 1 and up to 3 times ULN at baseline
  - Total bilirubin >3 x ULN irrespective of baseline levels

- Concurrent AST or ALT ≥ 3 × ULN and total bilirubin > 2 × ULN, irrespective of baseline levels

- Any Grade 4 treatment-related AE or laboratory abnormality, except for the following events which DO NOT require discontinuation:

  - Grade 4 amylase or lipase abnormalities that are not associated with symptoms or clinical manifestations, or radiographic signs of pancreatitis.
  - Isolated grade 4 electrolyte imbalances/abnormalities that are not associated with clinical sequelae and are corrected with supplementation/appropriate management within 72 hours of their onset.
  - Grade 4 lymphopenia and leukopenia.
  - Grade 4 treatment-related endocrinopathy adverse events, such as ACTH deficiency, hyper- or hypothyroidism, or glucose intolerance, which resolve or are adequately controlled with physiologic hormone replacement (corticosteroids, thyroid hormones) or glucose-controlling agents, respectively, may not require discontinuation after discussion with and approval from the Sponsors.

- Grade 3 and 4 adrenal insufficiency require discontinuation

- Any dosing interruption lasting >6 weeks, with the following exceptions: NOTE: Dosing interruptions >6 weeks that occur for non-drug-related reasons as well as for treatment of related AEs and subsequently tapering of steroids may be allowed if approved by the IND Sponsor. Patients must have had no recurrence of symptoms or new symptoms during steroid taper.

New immune related events or exacerbation of existing events during steroid treatment or taper suggest the presence of ongoing immune activation and should require permanent discontinuation.

- Any subjects who require additional immune suppressive treatment beyond steroids should go off study treatment

If nivolumab has to be permanently discontinued because drug-related toxicity, the patient will be permanently discontinued from entinostat treatment too.

In the case that a participant develops intolerance or side effect that is attributable to entinostat, the participant may, at his or her discretion, continue to receive only Nivolumab as otherwise indicated as long as they have received at least 1 full dose of both entinostat and nivolumab and nivolumab re-treatment criteria are met as outlined above.

6. ADVERSE EVENTS: LIST AND REPORTING REQUIREMENTS

6.1 Study Drugs

6.1.1 Entinostat

Commonly encountered AEs across clinical studies of entinostat monotherapy in patients with solid tumors included hypoalbuminemia, fatigue, nausea, hypophosphatemia, anemia, and thrombocytopenia. Incidence and severity were dose- and schedule-dependent. In a Phase 2, randomized, placebo-controlled study in patients with lung cancer, in which patients received erlotinib+entinostat or erlotinib+placebo, treatment-emergent adverse events (TEAEs) occurring at a ≥10% higher incidence in entinostat-treated patients versus placebo-treated patients included nausea (49% versus 25%); anorexia (40% versus 16%); weight decreased (32% versus 18%); dyspnea (31% versus 18%); vomiting (31% versus 13%); peripheral edema (28% versus 13%); anemia (22% versus 11%); thrombocytopenia (15% versus 3%); hypotension (14% versus 2%); and stomatitis (12% versus 2%). In a Phase 2 study in patients with metastatic melanoma treated with entinostat monotherapy, the most common TEAEs were nausea (39%), hypophosphatemia (29%), pain in extremity (21%), and back pain and diarrhea (each 18%) [122]. Additional clinical experience is summarized in the entinostat IB.

6.1.2 Nivolumab

Blocking PD-1 function may permit the emergence of auto-reactive T cells and resultant clinical autoimmunity. Rash/pruritus, diarrhea/colitis, pneumonitis, hepatitis, and hypothyroidism were drug-related, presumptive autoimmune events noted in previous nivolumab studies.

For the purposes of this study, a nivolumab-related AE is defined as an AE of unknown etiology, associated with drug exposure and is consistent with an immune phenomenon. Efforts should be made to rule out neoplastic, infectious, metabolic, toxin or other etiologic causes. Serological, immunological, and histological (biopsy) data should be used to support the diagnosis of an immune-mediated toxicity. Suspected nivolumab-related AEs must be documented on an AE CRF. Identification and treatment of nivolumab-related AEs can be found in **Section 4.4** as well as in **Appendix D**. Additional guidance can be found in the nivolumab IB.

6.2 Adverse Events Definition

6.2.1 Adverse Events

An AE is defined in the International Conference on Harmonisation (ICH) Guideline for Good Clinical Practice as "any untoward medical occurrence in a patient or clinical investigation patient administered a pharmaceutical product and that does not necessarily have a causal relationship with this treatment" (ICH E6:1.2).

Worsening of a pre-existing medical condition, (e.g., diabetes, migraine headaches, gout) should be considered an AE if there is either an increase in severity, frequency, or duration of the condition or an association with significantly worse outcomes.

Interventions for pretreatment conditions (e.g., elective cosmetic surgery) or medical procedures that were planned before study enrollment are not considered AEs.

Disease progression (PD) should not be recorded as an AE. If PD occurs, record the date first documented in the EOT visit eCRF. Also record all methods of assessment, e.g., 1 target/nontarget lesion, tumor response assessment, and/or clinical disease assessment. Indicate if the patient starts new treatment.

In the case of death, only record "Fatal" for the event causing death. AEs that are ongoing at the end of the study or time of death are to be noted as "continuing."

Classification of AEs is to be done by the Investigator is according to the NCI CTCAE, Version 4.03. The Death eCRF must also be completed.

The Investigator is responsible for reviewing laboratory test results and determining whether an abnormal value in an individual patient represents a significant change from baseline. In general, abnormal laboratory findings without clinical significance (based on the Investigator's judgment) should not be recorded as AEs; however, laboratory value changes requiring therapy or adjustment in prior therapy are considered AEs.

6.2.2 Suspected Adverse Reactions

A suspected adverse reaction is any AE for which there is a reasonable possibility that the drug caused the adverse event. For the purposes of Investigational New Drug Application (IND) safety reporting, 'reasonable possibility' means there is evidence to suggest a causal relationship between the drug and the AE. A suspected adverse reaction implies a lesser degree of certainty about causality than adverse reaction, which means any AE caused by a drug.

6.2.3 Expectedness

Unexpected AE: An AE, which varies in nature, intensity or frequency from information on the investigational drug/agent provided in the product IB, package insert or safety reports. Any AE that is not included in the IB consent is considered "unexpected".

Expected (known) AE: An AE, which has been reported in the IB. An AE is considered "expected", only if it is included in the IB document as a risk.

6.2.4 Serious Adverse Events

An AE or suspected adverse reaction is considered "serious" if, in the view of either the Investigator or Sponsor, it results in any of the following outcomes:

- is fatal
- is life-threatening (e.g., places the patient at immediate risk of death)
- requires in-patient hospitalization or prolongation of existing hospitalization for >24 hours
- results in persistent or significant disability/incapacity

- is a congenital anomaly/birth defect
- is an important medical event that may not result in death, be life-threatening, or require hospitalization but may be considered a serious adverse drug experience when, based upon appropriate medical judgment, it may jeopardize the patient and may require medical or surgical intervention to prevent one of the outcomes listed in this definition. Examples of such medical events include allergic bronchospasm requiring intensive treatment in an emergency room or at home, blood dyscrasias or convulsions that do not result in inpatient hospitalization, or the development of drug dependency or drug abuse.
- Potential drug induced liver injury (DILI) is also considered an important medical event.
- Hemophagocytic lymphohistiocytosis is also considered an important medical event.
- Suspected transmission of an infectious agent (eg, pathogenic or nonpathogenic) via the study drug is an SAE.
- Is a new cancer (that is not a condition of the study)
- Is associated with an overdose
- Is a pregnancy or pregnancy outcome of spontaneous abortion, missed abortion, benign hydatidiform mole, blighted ovum, fetal death, intrauterine death, miscarriage, or stillbirth.

A hospitalization meeting the regulatory definition for "serious" is any in-patient hospital admission that includes a minimum of an overnight stay in a health care facility. Any AE that does not meet one of the definitions of serious (e.g., emergency room visit, out-patient surgery, or requires urgent investigation) may be considered by the Investigator to meet the "other significant medical hazard" criterion for classification as a serious adverse event (SAE).

6.3 Reporting Procedures for All Adverse Events

The Investigator is responsible for ensuring that all AEs observed by the Investigator or reported by patients are properly captured in the patients' medical records and reported on the eCRF. The evaluation time period for all adverse events is from the first dose of the investigational agent to at least 30 days after the last dose of entinostat or nivolumab, or until resolution of all acute toxicities associated with study drug administration, whichever is longer, with the exception of SAEs, which must be documented through 100 days following cessation of treatment, or 30 days after the initiation of a new anticancer therapy, whichever is earlier.

The following AE attributes must be assigned by the Investigator:

- event description (with detail appropriate to the event);

- seriousness;
- dates of onset and resolution;
- severity;
- assessment of relatedness to entinostat and to nivolumab; the action taken.

Laboratory abnormalities: Laboratory abnormalities present at the screening visit will be recorded as pre-treatment signs and symptoms. After study treatment administration, all grade 3 and 4 clinical laboratory results that represent an increase in severity from baseline will be reported as AEs. A grade 1 or 2 clinical laboratory abnormality should be reported as an AE only if it is considered clinically significant by the investigator (induce clinical signs or symptoms or require therapy)

The Investigator may be asked to provide follow-up information, discharge summaries, and extracts from medical records.

If applicable, the study drug relationship will be assessed by means of the question: "Is there a reasonable possibility that the event may have been caused by one of the two drugs based on mechanism of action and/or toxicity profile, or by the combination of entinostat with nivolumab?" The relationship of an AE to the administration of the study drug is to be assessed by the investigator according to the following definitions:

- No (unrelated, not related, no relation): The time course between the administration of study drug and the occurrence or worsening of the adverse event rules out a causal relationship and another cause (concomitant drugs, therapies, complications, etc.) is suspected.

- Yes (related): The time course between the administration of study drug and the occurrence or worsening of the adverse event is consistent with a causal relationship and no other cause (concomitant drugs, therapies, complications, etc.) can be identified.

The following factors should also be considered:

- The temporal sequence from study drug administration - The event should occur after the study drug is given. The length of time from study drug exposure to event should be evaluated in the clinical context of the event.

- Underlying, concomitant, intercurrent diseases - Each report should be evaluated in the context of the natural history and course of the disease being treated and any other disease the subject may have.
- Concomitant medication - The other medications the subject is taking or the treatment the subject receives should be examined to determine whether any of them might be recognized to cause the event in question.
- Known response pattern for this class of study drug - Clinical and/or preclinical data may indicate whether a particular response is likely to be a class effect.
- Exposure to physical and/or mental stresses - The exposure to stress might induce adverse changes in the recipient and provide a logical and better explanation for the event.

The pharmacology and pharmacokinetics of the study drug - The known pharmacologic properties (absorption, distribution, metabolism, and excretion) of the study drug should be considered.

Whenever possible, the CTCAE, Version 4.03, should be used for assessing the severity of AEs (http://evs.nci.nih.gov/ftp1/CTCAE/CTCAE_4.03_2010-06-4_QuickReference_8.5x11.pdf).

For AEs that are not adequately addressed in the NCI CTCAE, the standard severity grading scale may be used (*Table 6.3.1*).

Table 6.3.1 Standard Severity Grading Scale

Grade	Standard Adverse Event Severity Scoring System	
1	Mild:	Aware of sign or symptom, but easily tolerated.
2	Moderate:	Discomfort enough to cause interference with usual activity.
3	Severe:	Incapacitating with inability to work or do usual activity.
4	Life-Threatening:	Refers to an event in which the patient was, in the view of the Investigator, at risk of death at the time of the event. (This category is not to be used for an event that hypothetically might have caused death if it were more severe.)
5	Fatal:	Event resulted in death.

It will be left to the Investigator's clinical judgment to determine whether an AE is related and of sufficient severity to require the patient's removal from treatment or from the study.

A patient may also voluntarily withdraw from treatment due to what he/she perceives as an intolerable AE. If either of these situations arises, the patient should be strongly encouraged to undergo an end-of-study assessment and be under medical supervision until symptoms cease or the condition becomes stable.

6.4 Serious Adverse Event Reporting Procedures

SAEs will be collected and recorded throughout the study period, from the first dose of the investigational agent through 100 days after the last dose of study drug or end of the study (if thought to be related to study drug), or 30 days after the initiation of a new anticancer therapy, whichever is earlier.

All SAEs, regardless of causality to study drug and/or administration device, will be promptly to the IND sponsor [redacted] **Syndax** [redacted] **and BMS** [redacted] **reported within 24 hours of discovery or notification of the event**.
Initial SAE information and all amendments or additions must be recorded on a Serious Adverse Event Report Form and provided to Syndax and BMS or their representative. The SAE reporting procedure is provided in **Appendix F**. If this falls on a weekend or holiday, an email notification is acceptable but must be followed by an SAE reporting form on the next business day.

After the initial SAE report, the investigator is required to proactively follow each subject and provide further information to the safety department in regards to the subject's condition. All SAE(s) will be followed until:

- Resolution
- The condition stabilizes
- The event is otherwise explained
- The subject is lost to follow-up
- Death

As soon as relevant information is available, a follow-up SAE report will be submitted to the IND Sponsor, BMS, and Syndax.

For all deaths, available autopsy reports and relevant medical reports should be provided to the IND Sponsor, Syndax and BMS or their representative. If a patient is permanently withdrawn from the study because of an SAE, this information must be included in the initial or follow-up Serious Adverse Event Report Form as well as the EOT eCRF.

The Investigator should notify the IRB or EC of SAEs occurring at the site and other AE reports received from Syndax and BMS, in accordance with local procedures and statutes.

6.5 Pregnancy and Lactation Reporting Procedures

Although pregnancy and lactation are not considered AEs, it is the responsibility of Investigators or their designees to report any pregnancy or lactation in a patient (spontaneously reported to them) that occurs during the study or within 120 days of completing the study or 30 days following cessation of treatment if the patient initiates new anticancer therapy, whichever is earlier. All patients who become pregnant must be followed to the completion/termination of the pregnancy.

Pregnancy outcomes of spontaneous abortion, missed abortion, benign hydatidiform mole, blighted ovum, fetal death, intrauterine death, miscarriage, and stillbirth must be reported as SAEs (Important Medical Events). If the pregnancy continues to term, the outcome (health of infant) must also be reported.

Such events must be reported within 24 hours to the Sponsor either by electronic media or paper.

6.6 Reporting of Overdose to the Sponsor

6.6.1 Entinostat Overdose

No information on the treatment of overdose of entinostat is currently available. Entinostat overdoses are defined as a single dose greater than 15 mg. This overdose will not be considered an SAE unless the outcome of the overdose meets seriousness criteria as defined in **Section 6.2.4** In the event of an entinostat overdose, the Sponsor should be immediately notified. The patient should be carefully monitored for potential adverse reactions and symptomatic treatment instituted as per institutional standards of care.

6.6.2 Nivolumab Overdose

A nivolumab overdose is considered an SAE. No specific information is available on the treatment of overdose of nivolumab. In the event of overdose, the patient should be observed closely for signs of toxicity. Appropriate supportive treatment should be provided if clinically indicated.

6.7 Follow-Up of Adverse Events

The Investigator must continue to follow all SAEs and non-serious AEs considered to be reasonably or possibly related to study drug either until resolution or the Investigator assesses them as chronic or stable. This follow-up may extend after the end of the study.

6.8 Reconciliation of SAEs

The Principal Investigator (or designee) will reconcile the clinical database SAE cases (case level only) transmitted to the IND sponsor and BMS Global Pharmacovigilance [redacted]. Frequency of reconciliation should be approximately every 3 months and prior to the database lock or final data summary. BMS GPVE will email, upon request from the Investigator, the reconciliation report. Requests for reconciliation should be sent to [redacted] and [redacted]. The data elements listed on the BMS GPVE reconciliation report will be used for case identification purposes. If the sponsor determines a case was not transmitted to the IND sponsor and/or BMS GVPE, the case should be sent immediately to the IND sponsor and BMS.

6.9 Safety Reporting to Health Authorities, Ethics Committees/Institutional Review Boards and Investigators

The Sponsor will send appropriate safety notifications to Health Authorities in accordance with applicable laws and regulations.

The Investigator must comply with any applicable site-specific institutional requirements related to the reporting of SAEs involving his/her patients to the EC/IRB that approved the trial.

In accordance with local regulations, the IND Sponsor or designee will notify investigators of all SAEs that are unexpected (i.e., not previously described in the IB), and related to the study drugs. This notification will be in the form of an expedited safety report (ESR) that is to be faxed to the investigators and the study coordinators. Upon receiving such notices, the investigator must review and retain the notice with the IB and where required by local regulations, the investigator will

submit the ESR to the appropriate IRB. The investigator and IRB will determine if the informed consent requires revision. The investigator should also comply with the IRB procedures for reporting any other safety information.

6.10 Food and Drug Administration (FDA) Reporting

All reporting to the FDA will be completed by the IND Sponsor.

7 Calendar-Day Telephone or Fax Report:

The IND Sponsor is required to notify the FDA of any fatal or life-threatening adverse event that is unexpected and assessed by the investigator to be possibly related to the investigational agent. Such reports are to be telephoned or faxed (301-796-9849) to the FDA within 7 calendar days of first learning of the event. Follow-up information will be submitted to the FDA as soon as relevant information is available.

10 Calendar-Day Written Report:

The IND Sponsor is required to notify the FDA of any SAE that is unexpected and related to the investigational agent in a written IND Safety Report.

Written IND Safety Reports should include an Analysis of Similar Events in accordance with regulation 21 CFR § 312.32. All safety reports previously filed with the IND concerning similar events should be included in the analysis. The new report should contain comments on the significance of the new event in light of the previous, similar reports.

Follow-up information will be submitted to the FDA as soon as relevant information is available.

IND Annual Reports:

In accordance with the regulation 21 CFR § 312.33, the IND Sponsor shall within 60 days of the anniversary date that the IND went into effect submit a brief report of the adverse events and progress of the investigation. Please refer to Code of Federal Regulations, 21 CFR § 312.33 for a

list of the elements required for the annual report. All IND annual reports will be submitted to the FDA by the IND Sponsor.

6.11 Protocol Deviations Due to an Emergency or Adverse Event

Departures from the protocol will be determined as allowable on a case-by-case basis and only in the event of an emergency. The Investigator or other physician in attendance in such an emergency must contact the PI as soon as possible to discuss the circumstances of the emergency.

The PI in conjunction with the Investigators, will decide whether the patient should continue to participate in the study. All protocol deviations and reasons for such deviations must be noted on the eCRF.

6.12 Safety Monitoring

The safety and tolerability of the investigational treatments will be monitored throughout the course of the study by the Investigators and the Sponsor Study Physicians(s).
The safety and tolerability data that will be reviewed will include, but are not limited to: AEs, unacceptable toxicities, laboratory test results, and patient discontinuations. Additionally, the development of SAEs will be assessed by the IND Sponsor Study Physician(s) on a continuous basis. (**NOTE**: all SAEs must be reported to the IND Sponsor, Syndax and BMS or designee within 24 hours of discovery or notification of the event [**Section 6.4**].)

No formal safety stopping rules are specified in the protocol. However, if any significant safety issues arise, a decision to modify or terminate the study will be made by the Sponsor in collaboration with the Investigators.

7. PHARMACEUTICAL INFORMATION

A list of the adverse events and potential risks associated with the investigational agents administered in this study can be found in **Section 6.1**. Additional drug information can be found in the investigational agent's Investigator Brochure.

[redacted]

[redacted]

[redacted]

[redacted]

[redacted]

[redacted]

[redacted]

[redacted]

[redacted]

[redacted]

[redacted]

[redacted]

[redacted]

[redacted]

[redacted]

[redacted]

7.2 Nivolumab (NSC #748726)

[redacted]

[redacted]

[redacted]

[redacted]

[redacted]

[redacted]

[redacted]

[redacted]

[redacted]

[redacted]

[redacted]

[redacted]

[redacted]

[redacted]

[redacted]

8. MEASUREMENT OF EFFECT

8.1 Antitumor Effect – Solid Tumors (RECIST 1.1)

Although the clinical benefit of these drugs has not yet been established, the intent of offering this treatment is to provide a possible therapeutic benefit, and thus the subjects will be carefully monitored for tumor response and symptom relief in addition to safety and tolerability. Subjects will be assessed by standard criteria for response per the timelines noted in the Study Calendar (**Section 10**).

Response and progression will be evaluated in this study using the new international criteria proposed by the revised Response Evaluation Criteria in Solid Tumors (RECIST) guideline (version 1.1) [123]. Changes in the largest diameter (unidimensional measurement) of the tumor lesions and the shortest diameter in the case of malignant lymph nodes are used in the RECIST criteria.

In addition, if a formal reading for RECIST measurements is performed, but the results are not available prior to the scheduled start of the next cycle, treatment should continue based upon review of the routine/clinical radiologist report of the status of disease (e.g., response, stability or progression). In the event that a RECIST re-read would have changed the treatment decision for the subject, if known prior, the IND Sponsor/designee should be consulted and course of action

clearly documented in the medical record. If it is in the best interests of the subject to continue on study, this is permissible. These cases will be reviewed in detail at the time of study analyses.

8.2 Definitions

Evaluable for toxicity: All patients will be included in the toxicity summaries, from the time of their first treatment with study drug.

Evaluable for objective response: All patients must be accounted for in the summary of objective response. To formally classify a patient as RECIST-evaluable, only those patients who have measurable disease present at baseline (one of the eligibility criteria, **Section 3.1**) and start treatment (received at least one dose of entinostat) will be considered evaluable. These patients will have their response classified according to the definitions stated below (**NOTE**: Subjects who exhibit objective disease progression prior to the end of cycle 1 will also be considered evaluable.)

8.2.1 Disease Parameters

Measurable disease: Measurable lesions are defined as those that can be accurately measured in [redacted] be recorded in millimeters (or decimal fractions of centimeters).

NOTE: Tumor lesions that are situated in a previously irradiated area might or might not be considered measurable. In most cases, in the event of post-radiation disease progression, previously irradiated lesions may be considered measurable lesions. Otherwise, these lesions should be considered non-measurable.

Malignant lymph nodes: To be considered pathologically enlarged and measurable, a lymph node must be ≥15 mm (≥1.5 cm) in short axis when assessed by CT scan (CT scan slice thickness recommended to be no greater than 5 mm [0.5 cm]). At baseline and in follow-up, only the short axis will be measured and followed.

Non-measurable disease: All other lesions (or sites of disease), including small lesions (longest

[redacted]

axis), are considered non-measurable disease. Bone lesions, leptomeningeal disease, ascites, pleural/pericardial effusions, lymphangitis cutis/pulmonitis, inflammatory breast disease, and abdominal masses (not followed by CT or MRI), are considered as non-measurable.

NOTE: Cystic lesions that meet the criteria for radiographically defined simple cysts should not be considered as malignant lesions (neither measurable nor non-measurable) since they are, by definition, simple cysts.
'Cystic lesions' thought to represent cystic metastases can be considered as measurable lesions, if they meet the definition of measurability described above. However, if non-cystic lesions are present in the same subject, these are preferred for selection as target lesions.

Target lesions: All measurable lesions up to a maximum of 2 lesions per organ and 5 lesions in total, representative of all involved organs, should be identified as target lesions and recorded and measured at baseline. Target lesions should be selected on the basis of their size (lesions with the longest diameter), be representative of all involved organs, but in addition should be those that lend themselves to reproducible repeated measurements. It may be the case that, on occasion, the largest lesion does not lend itself to reproducible measurement in which circumstance the next largest lesion which can be measured reproducibly should be selected. A sum of the diameters (longest for non-nodal lesions, short axis for nodal lesions) for all target lesions will be calculated and reported as the baseline sum diameters. If lymph nodes are to be included in the sum, then only the short axis is added into the sum. A sum of the longest diameter (LD) for all target lesions will be calculated and reported as the baseline sum LD. The baseline sum LD will be used as reference by which to characterize the objective tumor response.

Non-target lesions: All other lesions (or sites of disease) including any measurable lesions over and above the 5 target lesions should be identified as non-target lesions and should also be recorded at baseline. Measurements of these lesions are not required, but the presence, absence, or in rare cases unequivocal progression of each should be noted throughout follow-up.

8.2.2 Methods for Evaluation of Measurable Disease

All measurements should be taken and recorded in metric notation using a ruler or calipers. All baseline evaluations should be performed as closely as possible to the beginning of treatment and

never more than 28 days before the beginning of the treatment. Tumor response will be assessed per RECIST 1.1 using radiographic scans every 8 weeks±7 days.

The same method of assessment and the same technique should be used to characterize each identified and reported lesion at baseline and during follow-up. Imaging-based evaluation is preferred to evaluation by clinical examination unless the lesion(s) being followed cannot be imaged but are assessable by clinical exam.

Clinical lesions: Clinical lesions will only be considered measurable when they are superficial [redacted] calipers (e.g., skin nodules). In the case of skin lesions, documentation by color photography, including a ruler to estimate the size of the lesion, is recommended.
<u>Chest x-ray</u>: Lesions on chest x-ray are acceptable as measurable lesions when they are clearly defined and surrounded by aerated lung. However, CT is preferable.

<u>Conventional CT and MRI</u>: This guideline has defined measurability of lesions on CT scan based on the assumption that CT slice thickness is 5 mm (0.5 cm) or less. If CT scans have slice thickness greater than 5 mm (0.5 cm), the minimum size for a measurable lesion should be twice the slice thickness. MRI is also acceptable in certain situations (e.g., for body scans).

MRI has excellent contrast, spatial, and temporal resolution; however, there are many image acquisition variables involved in MRI, which greatly impact image quality, lesion conspicuity, and measurement. As with CT, if an MRI is performed, the technical specifications of the scanning sequences used should be optimized for the evaluation of the type and site of disease. Furthermore, as with CT, the modality used at follow-up should be the same as was used at baseline and the lesions should be measured/assessed on the same pulse sequence.

<u>PET-CT:</u> At present, the low dose or attenuation correction CT portion of a combined PET-CT is not always of optimal diagnostic CT quality for use with RECIST measurements. However, if the site can document that the CT performed as part of a PET-CT is of identical diagnostic quality to a diagnostic CT (with IV and oral contrast), then the CT portion of the PET-CT can be used for RECIST measurements and can be used interchangeably with conventional CT in accurately measuring cancer lesions over time. Note, however, that the PET portion of the CT introduces

additional data which may bias an investigator if it is not routinely or serially performed. Further, if the subject is allergic to contrast dye, the case should be discussed with the IND Sponsor.

Ultrasound: Ultrasound is not useful in assessment of lesion size and should not be used as a method of measurement. If new lesions are identified by ultrasound in the course of the study, confirmation by CT or MRI is advised. If there is concern about radiation exposure at CT, MRI may be used instead of CT in selected instances.

Endoscopy, Laparoscopy: The utilization of these techniques for objective tumor evaluation is not advised.

Tumor markers (CA 19-9): Tumor markers alone cannot be used to assess response. If markers are initially above the upper normal limit, they must normalize for a subject to be considered in complete clinical response.

Cytology: The cytological confirmation of the neoplastic origin of any effusion that appears or worsens during treatment when the measurable tumor has met criteria for response or stable disease is mandatory to differentiate between response or stable disease (an effusion may be a side effect of the treatment) and progressive disease.

FDG-PET: While FDG-PET response assessments need additional study, it is sometimes reasonable to incorporate the use of FDG-PET scanning to complement CT scanning in assessment of progression (particularly possible 'new' disease). New lesions on the basis of FDG-PET imaging can be identified according to the following algorithm:

- Negative FDG-PET at baseline, with a positive FDG-PET at follow-up is a sign of PD based on a new lesion.
- No FDG-PET at baseline and a positive FDG-PET at follow-up: If the positive FDG-PET at follow-up corresponds to a new site of disease confirmed by CT, this is PD. If the positive FDG-PET at follow-up is not confirmed as a new site of disease on CT, additional follow-up CT scans are needed to determine if there is truly progression occurring at that site (if so, the date of PD will be the date of the initial abnormal FDG-PET scan). If the positive FDG-PET at follow-up corresponds to a pre-existing site of disease on CT that is not progressing on the basis of the anatomic images, this is not PD.

- FDG-PET may be used to upgrade a response to a CR in a manner similar to a biopsy in cases where a residual radiographic abnormality is thought to represent fibrosis or scarring. The use of FDG-PET in this circumstance should be prospectively described in the protocol and supported by disease-specific medical literature for the indication. However, it must be acknowledged that both approaches may lead to false positive CR due to limitations of FDGPET and biopsy resolution/sensitivity.

NOTE: A 'positive' FDG-PET scan lesion means one which is FDG avid with an uptake greater than twice that of the surrounding tissue on the attenuation corrected image.

8.3 Response Criteria

8.3.1 Evaluation of Target Lesions

Complete Response (CR): Disappearance of all target lesions determined by two separate observations conducted not less than 4 weeks apart. Any pathological lymph nodes (whether target or non-target) must have reduction in short axis to <10 mm (<1 cm). There can be no appearance of new lesions.

Partial Response (PR): At least a 30% decrease in the sum of the diameters of target lesions, taking as reference the baseline sum diameters.

Progressive Disease (PD): At least a 20% increase in the sum of the diameters of target lesions, taking as reference the smallest sum on study (this includes the baseline sum if that is the smallest on study). In addition to the relative increase of 20%, the sum must also demonstrate an absolute increase of at least 5 mm (0.5 cm). (**NOTE:** the appearance of one or more new lesions is also considered progressions).

Stable Disease (SD): Neither sufficient shrinkage to qualify for PR nor sufficient increase to qualify for PD, taking as reference the smallest sum diameters while on study.

8.3.2 Evaluation of Non-Target Lesions

Complete Response (CR): Disappearance of all non-target lesions and normalization of tumor marker level. All lymph nodes must be non-pathological in size (<10 mm [<1 cm] short axis). **NOTE**: If tumor markers are initially above the upper normal limit, they must normalize for a subject to be considered in complete clinical response.

Non-CR/Non-PD: Persistence of one or more non-target lesion(s) and/or maintenance of tumor marker level above the normal limits.

Progressive Disease (PD): Appearance of one or more new lesions and/or unequivocal progression of existing non-target lesions. Unequivocal progression should not normally trump target lesion status. It must be representative of overall disease status change, not a single lesion increase.

Although a clear progression of "non-target" lesions only is exceptional, the opinion of the treating physician should prevail in such circumstances, and the progression status should be confirmed at a later time by the review panel (or PI).

8.3.3 Evaluation of Best Overall Response

The best overall response is the best response recorded from the start of the treatment until disease progression/recurrence (taking as reference for progressive disease the smallest measurements recorded since the treatment started).

The subject's best response assignment will depend on the achievement of both measurement and confirmation criteria (*Table 8.3.1*).

Table 8.3.1 For Subjects with Measurable Disease (e.g., Target Disease)

Target Lesions	Non-Target Lesions	New Lesions	Overall Response	Best Overall Response when Confirmation is Required[1]
CR	CR	No	CR	>4 wks confirmation

CR	Non-CR/Non-PD	No	PR	>4 wks confirmation
CR	Not evaluated	No	PR	
PR	Non-CR/Non-PD/not evaluated	No	PR	
SD	Non-CR/Non-PD/not evaluated	No	SD	Documented at least once >4 wks. from baseline
PD	Any	Yes or No	PD*	no prior SD, PR or CR
Any	PD**	Yes or No	PD*	
Any	Any	Yes	PD	

*A minority of subjects treated with immunotherapy may derive clinical benefit either delayed responses, stable disease, or increased overall survival despite initial evidence PD with nivolumab or combination treatment.

Subjects may be permitted to continue treatment beyond initial RECIST 1.1-defined PD (see **Section 4.9**). In these cases, a radiological assessment will be repeated 4 weeks later to confirm or not the progression disease, as defined by RECIST criteria.

** In exceptional circumstances, unequivocal progression in non-target lesions may be accepted as disease progression.

NOTE: Subjects with a global deterioration of health status requiring discontinuation of treatment without objective evidence of disease progression at that time should be reported as "symptomatic deterioration;" (e.g., weight loss >10% of baseline body weight; worsening of tumor-related symptoms, decline in performance status >1 left on ECOG scale) after discussion with IND Sponsor. .Every effort should be made to document the objective progression even after discontinuation of Treatment.

8.3.4 Confirmation of Response

Confirmation of response is required per RECIST 1.1.

8.3.5 Duration of Response

Duration of overall response: The duration of overall response is measured from the time measurement criteria are met for CR or PR (whichever is first recorded) until the first date that recurrent or progressive disease is objectively documented (taking as reference for progressive disease the smallest measurements recorded since the treatment started) or death. Subjects who remain alive and have not progressed or received new therapy will be censored on the date of last protocol specified tumor assessment. Subjects who receive new therapy will be censored at the start of the therapy.

Duration of stable disease: The duration of stable disease is measured from the start of the treatment until the first date that recurrent or progressive disease is objectively documented (taking as reference the smallest measurements recorded since the treatment started, including the baseline measurements) or death. Subjects who remain alive and have not progressed or received new therapy will be censored on the date of last protocol specified tumor assessment. Subjects who receive new therapy will be censored at the start of the therapy.

8.3.6 Progression-Free Survival

Progression-free survival (PFS) is defined as the duration of time from start of treatment to time of progression per RECIST1.1 or death whichever comes first. PFS for patients who are not progressed at the time of data analysis will be censored on the date of the latest tumor assessment. Patients who go off treatment and begin alternate therapy, prior to progressing on this protocol will be censored at the time on the date of the latest tumor assessment before starting the alternate therapy. We assume that all patients have radiographic scans during screening period and before starting treatment. The following table describes how PFS event is defined and PFS time is calculated. Only radiographic scans used to tumor assessment per RECIST 1.1 before starting the alternate therapy will be counted if patients go off treatment and begin alternate therapy. The following table shows how PFS is defined in various scenarios (*Table 8.3.2*).

Table 8.3.2 Definition of PFS

Scenario	PFS event (1) or censor (0)	PFS time
At least one post treatment scan is available and tumor response is evaluated according to RECIST 1.1 and all scans are completed on the schedule or off the schedule within a month and patient is alive at the latest follow-up.	1 if PD<br>0 if no PD	The date of the latest tumor assessment – the date of starting treatment

At least one post treatment scan is available and tumor response is evaluated according to RECIST 1.1, but the gap between radiographic scans is greater than 3 months. No PD is observed on the latest scan before the gap occurs.	0	The date of the latest tumor assessment before the gap– the date of starting treatment
No disease progression per RECIST 1.1 is observed on the post-treatment scans and patient dies before the next scheduled scan + 1 month.	1	The date of death – the date of starting treatment
No disease progression per RECIST 1.1 is observed on the post-treatment scans and patient dies. The period from the latest scan to death is greater than 3 months.	0	The date of the latest tumor assessment – the date of starting treatment
No post-treatment scan is available and patient dies before the first scheduled scan + 1 month.	1	The date of death – the date of starting treatment
No post-treatment scan is available and patient is lost follow up or patient dies >3 months later after starting treatment.	0	0

8.3.7 Overall survival

Overall survival is defined as the time from start of treatment until death due to any cause. If patient is still alive at the time of analysis, OS will be censored on the date of last known being alive.

8.4 Safety/tolerability

Analyses of safety/ toxicity will be performed for all patients having received at least one dose of study drug. The study will use the CTCAE version 4.03 for reporting of adverse events (http://ctep.cancer.gov/reporting/ctc.html).

9. BIOMARKER, CORRELATIVE, AND SPECIAL STUDIES

9.1 Overview

The major hypothesis of our correlative studies are that:

1) Treatment with entinostat will result in changes in TME enhancing T cell effectors and/or reducing suppressive ability of CD4+FoxP3+ T regulatory cells and MDCSs in the tumors.
2) Immunologic changes in biopsy specimens will correlate with subsequent clinical response to anti-PD-1/PD-L1 therapy.

Explorative correlative studies will be performed on biopsy specimens (pre - and post- entinostat treatment and approximately after 6 weeks from Cycle 1 day 1) and blood samples.

Mandatory research bloods and tumor biopsies will be obtained at baseline (day -14), after two weeks of entinostat lead in period (on approximately day 14 from the first entinostat administration), and approximately after 6 weeks from Cycle 1 day 1 visit. (see Study Calendar, **Section 10**). Biopsies at progression are optional.

These three mandatory biopsies will provide valuable information regarding the mechanism of action of entinostat and the impact on the TME when PD1 inhibition is added in, as well as primary resistance mechanisms to the strategy.

NOTE: All samples will be collected as outlined below, initially processed, and stored/bank until future analysis.

9.2 Collection of Specimens

9.2.1 Tissue Samples

Mandatory and optional tumor biopsies will be obtained at the time points as outlined above and on the Study Calendar.

Subjects will undergo a direct or image-guided research biopsy procedure. FNA and on-site cytopathologic evaluation, where appropriate, will be done for sample/site adequacy followed by

core biopsies for collection of tissue for the research correlates outlined. The biopsy site will be clearly documented in the records; baseline and each follow-up biopsy will preferentially be done in the same organ site.

Approximately six (6) core biopsy specimens will be obtained with each biopsy as outlined; the cytopathology slide will also be retained and submitted, if performed. Up to three cores will be placed in formalin solution and paraffin embedded. The remaining biopsy specimens will be flash frozen in liquid nitrogen and then stored as fresh tissue at ≤ -70°C for extraction of DNA and RNA. The biopsy specimens will be stored and batch shipped at the latest at the conclusion of study enrollment.

The study staff will be notified when a biopsy is taking place. If core biopsy is not possible, other methods may be approved in advance by the IND Sponsor/designee.

If a subject discontinues the study prior to a planned follow-up biopsy, an attempt should be made to collect the next scheduled biopsy when the decision is made to discontinue and prior to initiation of additional treatment, if possible.

In the event that a subject cannot undergo a second biopsy for any reason, including refusal, or the baseline biopsy was unsuccessful or invaluable, the second and the third biopsies, as well as the optional biopsy at the time of progression, will not be required and the subject will continue to be treated per study protocol.

NOTE: Archival tumor specimens from previously collected samples will be obtained for correlative analysis, if available.

Biopsy Instructions

Acceptable biopsy sites are:

- Percutaneous biopsy of deep sites with local anesthetic and/or sedation (with an expected risk of severe complications < 2%)
- Cutaneous biopsy of palpable sites with local anesthetic and/or sedation with an expected risk of severe complications < 2%
- No open surgical or laparoscopic procedures will be performed solely to obtain a biopsy for this protocol.

Such biopsies can be safely performed as evidenced by literature reports [122, 123], as well as, in the prior experience of our PI at the NIH Clinical Center [124], where, among 244 research tumor biopsies (18G needle) in liver (126), subcutaneous/chest wall/abdominal wall (36), intramuscular (18), lung and lymph nodes, there were 8 minor cases of bleeding, and one pneumothorax requiring 48h hospitalization (21). This evidence support that serial percutaneous core-needle biopsies can be obtained safely yielding material applicable for multiple translational applications.

Considering the tumor histologies to be enrolled in this study are pancreatic cancer and cholangiocarcinoma, biopsies will probably be of liver. In our institution, the expected complication rate of percutaneous liver biopsy similar to those that will be done for this trial is less than 1 %. Risks of the procedure include, but are not limited to, bleeding, infection, pain, and scarring.

9.2.2 Blood Samples

Whole blood for PBMCs (up to 180cc) will be collected at the specified timepoints (See Section 10). Detailed instructions for blood collection, processing, and storage are provided in the Laboratory Manual.

Sera (up to 10 cc) and plasma (up to 20cc) will be collected at the specified timepoints (See Section 10). Detailed instructions for blood collection, processing, and storage are provided in the Laboratory Manual.

In the event that a subject cannot have all blood samples or volume needed at a single visit, the samples and/or remaining samples should be collected at the next possible visit.

9.2.3 Handling and Shipping of Specimens

A unique subject identifier will be assigned to each subject by the coordinating center and will be used to label the samples. The protocol scientific investigator(s) analyzing the samples will be blinded as to the direct subject identifiers.

A **Laboratory Manual** will be provided for specific handling and processing instructions; this document will be disseminated with the clinical protocol and is also available to investigators on request from the Coordinating Center.

After shipping, samples, and associated data, will be stored at Johns Hopkins unless the subject withdraws consent.

9.3 Methods

9.3.1 Immunohistochemistry (IHC) and Gene Array Analyses

The density of $CD8^+$ Teffs, PD-1+, $FoxP3^+$ Tregs, and the number of MDSCs will be evaluated by IHC in tumor specimens. Human PD-L1 IHC staining of paraffin embedded tumor specimens will be done using the Dako Catalyzed Signal Amplification system [126]. The density of cells will be determined at the junction between tumor tissue and non-tumor tissue (invasive front) and within the tumor (TILs). The densities will be reported as positive cells / mm^2 of tissue using digital analysis. Each slide will be digitally scanned at 20X magnification and areas to be analyzed will be indicated using Aperio software. PD-L1 expression is considered positive if membranous staining is present in more than 5% of the neoplastic cells [127]. In addition, immune aggregates will be microdissected and evaluated for immune signatures using gene array analyses. These studies will be conducted as previously described [128].

9.3.2 Next Generation Sequencing (NGS)

Tumor specific somatic mutations will be identified by whole-exome sequencing (WES) using DNA isolated from tumor tissue, along with subject-matched normal lymphocytes. Genomic DNA from tissue and lymphocyte sample will be sonicated to a modal size of 200 bp and ligated to Illumina TruSeq adaptors with barcodes, and subjected to minimal amplification. The resulting libraries will be subjected to selection of all exonic sequences in the genome via hybrid-capture with 120 bp biotinylated RNA-baits using the Agilent SureSelect platform. Enriched exonic sequences will be further amplified and subject to massively parallel sequence-by-synthesis 100x100 bp paired end sequencing on an Illumina HiSeq 2000 at a minimal depth of 75x average coverage. The resulting reads will be aligned to the genome using bowtie2 and variants will be called on each sample compared to the human reference using the GATK software. Somatic variants will be identified using the strelka software.

The T cell repertoire in TILs will be evaluated in tumor specimens using Adaptive Biotechnologies' next-generation sequencing-based approach.

9.3.3 HLA-Binding Prediction Algorithms and Elispot Assays

Quantification of tumor neoantigen-specific $CD8^+$ T cell levels in PBL: Each of the somatic missense mutations identified by WES will be screened for potential HLA class I-binding neoepitopes using the predictive algorithm NetMHC. 19mer peptides will be synthesized spanning each mutation predicted to generate a neoeptiope; and used in IFN ELISPOT assays to measure neoepitope-specific $CD8^+$ T cell levels in PBL.

9.3.4 MDSC Analysis

Levels of granulocytic and monocytic MDSCs in peripheral blood will be evaluated by 6-color fluorescence-activated cell sorting (FACS) using monoclonal antibodies specific to CD11b, CD14, CD15, CD33, HLA-DR and the IL-4 receptor.

10. STUDY CALENDAR

Notations/reminders for all calendars:

- Baselines assessments are to be conducted within 28 days of registration unless otherwise noted. ▯ Correlative blood and tissue sample participation is mandatory.
- Additional tests may be performed at the discretion of the treating investigator as per routine practice, or as otherwise clinically indicated.

The schedule should be followed as closely as is realistically possible; the schedule may be modified due to problems such as scheduling delays or conflicts (e.g., clinic closure, vacations, etc.) with the guidance of the IND Sponsor/designee, as appropriate, and will not be reportable as a deviation unless the endpoints of the study are affected.

* Required ≤ 7 days prior to the first dose of entinostat

** Required assessment windows during combination treatment for each cycle as noted (e.g., Days 1 and 15 is 3 days, weekly is ±1 day). Assessments on Days 1 and 15 should be completed/reviewed prior to planned nivolumab administration.

*** After 4 months, nivolumab will be given at a dose of 480 mg fixed dose every 4 weeks (maintenance phase). Therapy may continue with entinostat 5 mg weekly plus nivolumab 480 mg every 4 weeks, until intolerance or progression.

Patients will be assessed in clinic on D1 of every cycle, and will be contacted by phone weekly, on the weeks entinostat alone is self-administered at home.

Required ≤ 14 days prior to the first dose of entinostat

NOTE: All timeframes noted after the lead-in phase refer to duration on combination treatment (e.g., first-restaging should occur after 8 weeks of initiation of combination treatment of entinostat and nivolumab.)

Table 10.1 Study Calendar

Procedure	Baseline[1]	Lead-In Day -14*	Lead-In Day -7	Every cycle ** (1 cycle = 28 days)				Week 6 (+14 days)	Maintenance Phase	Every 8 weeks (±7 days)[2]	EOT[3] 28 days After Last Dose (±7 days)
				Day 1 (±3 days)	Day 8 (±1 day)	Day 15 (±3 days)	Day 22 (±1 days)		Every 4 weeks, Beginning on week 16***		
Informed Consent & Review of Eligibility Criteria	X										
Medical History & Demographics	X										
Physical Exam[4]	X	X[#]		X				X	X		X
Vital Signs, Height (only baseline), Weight	X	X		X				X	X		X
ECOG Performance Status	X	X		X		X		X	X		X
Oxygen Saturation	X			X		X		X	X		X
Hematology Panel[5]	X	X		X		X		X	X[6]		X
Chemistry Panel[7]	X	X		X		X		X	X		X
Coagulation Panel [8]	X										
Thyroid Function[9]	X			X		X		X	X		X
Hepatitis Serologies[10]	X										
Electrocardiogram (ECG) [11]	X										

Procedure	Baseline[1]	Lead-In Day -14*	Lead-In Day -7	Every cycle ** (1 cycle = 28 days)				Week 6 (+14 days)	Maintenance Phase	Every 8 weeks (±7 days)[2]	EOT[3] 28 days After Last Dose (±7 days)
				Day 1 (±3 days)	Day 8 (±1 day)	Day 15 (±3 days)	Day 22 (±1 days)		Every 4 weeks, Beginning on week 16***		
Urinalysis[12]	X										
Pregnancy test/ Assessment WOCBP[13]	X	X*		X					X		
Tumor Assessments Imaging[14]	X									X	X[20]
CA19-9		X*								X	X[20]
Entinostat[15]		X	X	X	X	X	X	X	X		
Nivolumab[16]				X		X		X	X		
Peripheral blood for PBMC (up to 180cc)[17]		X		X				X			X
Peripheral blood for plasma (up to 20cc)[17]		X		X				X			X
Serum (up to 10cc)[17]		X		X				X			X
Fresh Tumor Biopsy[18]		X		X[18]				X[19]			X[20]
Archival Tumor Sample[21]	X										
Concomitant Medication Review	X	X		X		X		X	X		X
Symptom/Toxicity Assessment[22]			X	X		X		X	X[22]		X

Procedure	Baseline[1]	Lead-In Day -14*	Lead-In Day -7	Every cycle ** (1 cycle = 28 days)				Week 6 (+14 days)	Maintenance Phase	Every 8 weeks (±7 days)[2]	EOT[3] 28 days After Last Dose (±7 days)
				Day 1 (±3 days)	Day 8 (±1 day)	Day 15 (±3 days)	Day 22 (±1 days)		Every 4 weeks, Beginning on week 16***		
Follow-up/Survival[23]											X[24]

Study Calendar: Entinostat and Nivolumab

1. Baseline evaluations should occur within 28 days prior to registration, unless otherwise noted. **NOTE**: No window applies to demographics, informed consent, or height at baseline. If any assessments are repeated after registration and prior to dosing on Day -14, subjects must maintain eligibility requirements in order to initiate therapy. Biopsies should be performed only after patient is deemed eligible and registered on the trial.
2. Disease assessments will be conducted every 8 weeks (+/- 1 week) from Cycle 1 Day1 for the first 6 months; this timeframe may then be extended to every 12 weeks (+/- 1 week) at the discretion of the treating provider.
3. A mandatory End of treatment (EOT) visit should be performed 28 days (± 7 days) after the last dose of entinostat to determine patients' safety outcome. Repeat any physical or laboratory assessments after the last dose of any study treatment if last assessment >7 days prior.
4. Complete physical exam will be completed at baseline; focused physical examinations may be conducted thereafter.
5. Hematology Panel includes at a minimum: basophils, eosinophils, hematocrit, hemoglobin, lymphocytes, monocytes, neutrophils, platelet count, and total white cell count.
6. Patients receiving entinostat in the maintenance phase will have hematology panel (as defined above) checked every two weeks (either in clinic or at home). When done at home, patients will be instructed to send the results to the study team and will communicate the results via fax to the study team
7. Chemistry panel includes (if possible): albumin, alkaline phosphatase, total bilirubin [nitrogen if total bilirubin is ≥2xULN (and no evidence of Gilbert's syndrome) then fractionate into direct and indirect bilirubin)], bicarbonate, BUN, calcium, chloride, creatinine, glucose, potassium, total protein, SGOT [AST], SGPT [ALT], sodium, LDH, magnesium, amylase and lipase.
8. INR (international normalized ratio), PT (prothrombin time) and PTT (partial thromboplastin time) required at screening and within 30 days of image-guided research biopsies. If patient receives a biopsy at disease progression, the coagulation panel must be completed within 30 days prior to biopsy.

9. Thyroid function will be assessed by TSH (Thyroid Stimulating Hormone) at baseline and prior to each dose of nivolumab. If abnormal, Free T3 (triiodothyronine) and T4 (thyroxine) should be assessed.
10. The following should be assessed at baseline: Hepatitis B surface antigen (HBV sAg) and Hepatitis C antibody (HCV Ab) or Hepatitis C RNA (HCV RNA).
11. An ECG is required during screening (prior to starting study treatment), as well as at any other time point when clinically indicated. All 12-lead ECGs should be recorded while the subject is in the supine position. The same method of assessment should be used throughout the study. Twelve-lead ECGs will be obtained after the subject has been resting in a supine position for at least 5 minutes.
12. Bilirubin, blood, glucose, ketones, pH, protein, specific gravity, color and appearance. Microscopy should be used as appropriate to investigate white blood cells and use the high power field for red blood cells
13. For WOCBP (women of childbearing potential) only. A serum or urine pregnancy testing is required prior to study enrollment/registration, ≤7 days prior to the first dose of entinostat and <24 hours prior to the first dose of nivolumab. After the first dose of nivolumab, an interim history to evaluate any risk of exposure to a fetus must be done prior to each dose in lieu of routine HCG testing, if appropriate.

    Female subject of childbearing potential should have a negative urine or serum pregnancy test within 72 hours prior to receiving retreatment with study medication. NOTE: If a patient has a positive or indeterminate serum or urine pregnancy test, then an ultrasound must be done to rule out pregnancy to enroll on trial. A serum B-HCG value of <25 is considered a negative pregnancy test.
14. Radiologic imaging of chest/abdomen/pelvis (CT preferred, abdominal/pelvis MRI will be performed for patients with CT contrast allergy) and any other imaging required per RECIST for the particular subject are required at baseline. A baseline bone scan or PET/CT could be required to assess bone disease in patients with suspected or known bone metastases. A baseline brain CT scan or MRI is requested in patients with suspected or known brain metastases. Repeat same modality(ies) in follow-up to follow known site(s) of disease, with other assessments as clinically indicated if one has not been done within the past 6 weeks. .
15. Entinostat is to be self-administered by the patient weekly.
16. Patients will receive entinostat 5 mg oral once a week plus nivolumab 240 mg every two weeks for approximately four months. After 4 months (16 weeks), nivolumab will be given at a dose of 480 mg fixed dose every 4 weeks. Therapy may continue with entinostat 5 mg weekly plus nivolumab 480 mg every 4 weeks, until intolerance or progression.

17. Blood for serum and plasma samples will be collected at baseline (D -14), after entinostat lead in (on approximately day 14 (D-1) of entinostat therapy), on week 6, prior to dosing on C2D15, and at the time of the EOT visit. Detailed instructions for blood collection, processing, and storage are provided in the Laboratory Manual.

18. Mandatory fresh tissue samples will be collected at D-14 and after entinostat lead-in (on approximately day 14 (D-1) of entinostat therapy prior to dosing on C1D1) and on week 6, prior to dosing on C2D15. **A ±3 day window applies to both time points.** All

samples are collected pre-dose on treatment days). Approximately six (6) core biopsy specimens will be obtained with each biopsy as outlined; the cytopathology slide will also be retained and submitted, if performed (See **Section 9.2.1**) All correlative blood/tissue studies should be performed on the same day.

NOTE: If applicable, the daily dose of entinostat may occur prior to hematology/chemistry tests and these laboratory tests may be drawn at the same time as the correlative blood samples.

19. A third mandatory biopsy will be performed at week 6 from Cycle 1, Day1. A window of +14 days applies to this time point

20. An optional biopsy will be performed at the time of disease progression.

21. Archival tumor specimens from previously collected samples will be obtained for correlative analysis, if available.

22. Weekly assessments for toxicity/adverse events may occur by phone on days when entinostat alone is administered. Additionally, assessments are to continue for 30 days after the last dose of nivolumab (at intervals <21 days) or to resolution of drug-related AEs to ≤ Grade 1, whichever occurs later. SAEs that occur within 100 days of the last treatment or before initiation of a new antineoplastic treatment should also be followed and recorded. These may occur by phone for subjects no longer being seen at the institution; however, an in-person clinic visit may be needed to confirm event resolution and/or to effectively evaluate possible immune-related events. Subjects must be told of follow-up requirements at study entry.

23. Subjects who complete or discontinue from treatment because of progression disease will be followed (by phone or email) every three months (+/- 2 weeks) after completion of the EOT visit for up to 2 years or study closure to monitor overall survival. Information of other cancer therapies after discontinuation from the study treatment will also be collected.

For subjects who discontinue treatment for reasons other than tumor progression, will be contacted (by phone or email) every three months (+/- 2 weeks) after completion of the EOT for up to 2 years or study closure to monitor overall survival, and information

about disease status will be also collected if possible (i.e. radiological imaging and/or tumor markers) until documented tumor progression.

NOTE: For evaluation of PFS, all subjects who discontinue study drug administration prior to 6 months must have restaging scans/imaging within a 7 day window of the 6 month time point from the date of initiation of combination treatment

11. DATA REPORTING / REGULATORY REQUIREMENTS

Adverse event lists, guidelines, and instructions for AE reporting can be found in **Section 6** (Adverse Events: List and Reporting Requirements).

11.1 Data Management

All information will be collected on study-specific case report forms (CRFs) by study staff. These data will be reviewed for completeness and accuracy by the PI.

CRFs will be used to capture study results and data. The study coordinator or other authorized study personnel will transcribe data from source documents onto eCRFs. Before or between visits, the IND Sponsor,, Syndax and BMS or designee may request copies of the CRFs for preliminary medical review. Once the CRFs are complete and source-verified, the investigator must sign and date all required pages, verifying the accuracy of all data contained within the CRF. Training will be provided on proper completion of CRFs.

11.2 Data Confidentiality and Subject Anonymity

All information about the nature of the proposed investigation (with the exception of information required by law or regulations to be disclosed to the IRB, the subject or the appropriate regulatory authority) must be kept in confidence by the entire study personnel.

The anonymity of participating subjects must be maintained. Subjects will be identified by their initials and an assigned subject number on CRFs and other documents retrieved from the site or sent to the IND Sponsor, Syndax and BMS, study monitor, regulatory agencies, or central laboratories/reviewers. Documents that identify the subject (e.g., the signed ICF) must be maintained in strict confidence by the investigator, except to the extent necessary to allow auditing by the appropriate regulatory authority, the study monitor, IND Sponsor or their representative.

11.3 Protocol Compliance

Substantive changes in the protocol include changes that affect the safety of subjects or changes that alter the scope of the investigation, the scientific quality of the study, the experimental design, dosages, assessment variable(s), the number of subjects treated or the subject selection criteria.

A protocol amendment must receive IRB approval before implementation. In parallel with the IRB approval process, the protocol amendment will be submitted to the appropriate regulatory authority as an amendment to the regulatory submission under which the study is being conducted. If a protocol amendment requires changes in the ICF, the revised ICF and protocol prepared by the investigator must also be approved by the IND Sponsor, Syndax/BMS, and the IRB before implementation.

Emergency departures from the protocol that eliminate an apparent immediate hazard to a particular subject and that are deemed crucial for the safety and well-being of that subject may be instituted for that subject only. The investigator or the attending physician also will contact the IND Sponsor and/or designee as soon as possible in the case of such a departure. These departures do not require preapproval by the IRB; however, the IRB and the IND Sponsor and/ or designee must be notified in writing as soon as possible after the departure has been made. In addition, the investigator will document in the subject's CRF the reasons for the protocol deviation and the ensuing events.

11.4 Safety meetings

Scheduled meetings will take place weekly and will include the protocol PI, study coordinator(s), research nurse (s), and sub-investigators (as appropriate). During these meetings matters related to the following will be discussed: safety of protocol participants, validity and integrity of the data, enrollment rate relative to expectation, characteristics of participants, retention of participants, adherence to protocol (potential or real protocol violations), data completeness, and progress of data for objectives.

Quarterly teleconferences will be scheduled to include the Investigator and Syndax and BMS representatives. During these meetings, the Investigator shall provide Syndax and BMS with study progress updates. The Investigator will provide a summary of key points from the weekly meetings with a focus on safety of the protocol participants, enrollment status, and progress of data for objectives. In addition, both Syndax and BMS will provide safety and applicable program updates to the Sponsor.

11.5 Monitoring

The SKCCC Compliance Monitoring Program will provide external monitoring for JHU-affiliated sites in accordance with SKCCC DSMP (Version 6.0, 02/21/2019). The SMC Subcommittee will determine the level of patient safety risk and level/frequency of monitoring.

Dr. Nilofer Saba Azad will be holding the IND for this study. She will comply with all regulated reporting requirements to the FDA.

12. STATISTICAL CONSIDERATIONS

12.1 Overall Study Design

This is an open-label, two-stage, phase 2 study evaluating the clinical activity and safety of entinostat plus nivolumab. We will enroll two cohorts of patients: unresectable or metastatic cholangiocarcinoma (Cohort 1) and unresectable or metastatic pancreatic adenocarcinoma (Cohort 2). Patients must have progressed (or were intolerant) to 1 or 2 prior chemotherapy regimens in the metastatic setting, or progression < 6 months from the completion of adjuvant chemotherapy.

The primary objective of the trial is to determine whether the combination of entinostat plus nivolumab yields a clinically compelling antitumor activity measured as objective response rate (ORR, assessed by RECIST 1.1). Secondary endpoints include safety, progression-free survival (PFS), overall survival (OS), and immunologic correlates.

The study is planned with 27 evaluable subjects per histology, based on a two-stage design that allows early termination for lack of efficacy.

12.2 Sample Size and Accrual Rate

Approximately 27 patients with unresectable or metastatic CCA and 27 patients with locally advanced or metastatic PDAC will be enrolled, with ORR as the primary endpoint. Enrollment will be carried out in 2 stages so that the study can terminate early if the combination of entinostat plus nivolumab is not sufficiently effective.

Simon's two-stage, minimax, design will be employed in each cohort to test the null hypothesis that the true ORR is 5% or less (not considered clinically compelling for this combination). In the first stage, 13 subjects will be accrued.

If there are no responders among the first 13 subjects, the study will be terminated for futility. Otherwise, 14 additional subjects will be accrued to target a total of 27 treated and response evaluable subjects. The null hypothesis will be rejected if 4 or more responses are observed in 27 subjects per each cohort.
The probability of stopping the trial early for futility is 51% if the true ORR is 5% or less. This design yields 80% power at a one-sided type I error rate of 5% when the true response rate is 20% The study may enroll 33 subjects per cohort to ensure 27 would be evaluable for the primary efficacy endpoint. We expect to enroll 4-6 patients per month. We estimate that we could reach the accrual goal within 1 year.

Any patient who comes off of study due to toxicity before completing the first cycle of treatment, without evidence of disease progression may be replaced.

12.3 Study Endpoints Analyses

12.3.1 Analysis of Primary Endpoints

The objective response rate defined as the proportion of response evaluable subjects who have a CR or PR using RECIST 1.1 at any time during the study.

The evaluable population includes all subjects who have completed at least one dose of entinostat..

An exact binomial test will be used to evaluate the primary question of whether the response rate for combination therapy exceeds the historic rate for the single agent. Response rate will be reported with exact confidence interval.

12.3.2 Analysis of Secondary Endpoints

- OS is defined as the duration of time from start of study treatment to time of death. For subjects lost to follow-up or whose vital status is unknown, every effort will be made to determine the date such subjects were last known to be alive. Such efforts may include phone calls, certified mail, and the checking of public records. Subjects who are alive or lost to follow-up as of the data analysis cutoff date will be right-censored. The censoring date will be determined from the date the subject was last known to be alive.

- PFS is defined as the time from cycle 1, day 1 of immunotherapy until first documented local progression or death due to any cause. Disease progression will be assessed using RECIST (version 1.1). Due to the expectation that some patients may experience delayed clinical responses to therapy, patients with disease progression by radiographic imaging or laboratory parameters during a 12-week evaluation period but without rapid clinical deterioration or significant change in performance status that requires additional immediate therapy may continue to receive treatment on study.

  Subjects that meet the above criteria and continue on study therapy must discontinue treatment upon documentation of disease progression on the second scan. The date of progression will be backdated to the time of first RECIST criteria progression. Tumor assessments will be made using RECIST 1.1 and immune-related response criteria (irRC). Individuals will be censored at the date of the last scan for PFS if no event occurs.

  Median time to event will be calculated and reported with confidence intervals.

  Analysis of PFS will be restricted to evaluable individuals who complete at least one dose of entinostat, while the analysis of OS will be performed on all evaluable patients.

  OS curves, OS medians with 95% CIs, and OS rates at 6, 12, 24 and 36 months with 95% CIs will be estimated using Kaplan-Meier methodology

  PFS curves, PFS medians with 95% CIs, and PFS rates at 6, 12 and 24 months with 95% CIs will be estimated using Kaplan-Meier methodology.

- Duration of response (DOR) will be calculated for subjects who achieve a best overall response of CR or PR with entinostat in combination with nivolumab. For such subjects,

duration of response is defined as the number of weeks from the start date of PR or CR (whichever response is recorded first) and subsequently confirmed to the first date that recurrent or progressive disease or death is documented. In such cases, recurrent or progressive disease will be assessed relative to the smallest tumor measurements recorded since the start of study treatment. The primary analysis of duration of response will be based on the time point responses, best overall response, and PD status that are determined by the review of the investigator

DOR will be summarized descriptively using the Kaplan-Meier method with 95% confidence intervals (CIs)

- The safety analysis will be performed in all subjects who receive any amount of study drug. A baseline measurement and at least 1 laboratory or other safety-related measurement obtained after at least 1 dose of study treatment may be required for inclusion in the analysis of a specific safety parameter (e.g., lab shifts from baseline).

  A complete list of all AE data will be provided along with an assessment of NCI CTCAE grade and relationship to study drug. The incidence of AEs will be tabulated by subgroups of interest (e.g. grade 3 or higher, organ class, relationship to study drug).

  For analyses at the individual level, the highest grade and relationship to study drug will be assumed if multiple events have occurred.

Toxicity will be tabulated by type and grade and will be summarized with descriptive statistics. Other safety data will be assessed in terms of physical examination, clinical chemistry, hematology, vital signs, and ECGs. Negative binomial regression and Cox proportional hazards models will be used to assess the rate of AE and time to first toxicity, respectively.

12.3.3 Data & Safety Monitoring

The PI and the study statistician will review the study as needed based upon rate of accrual for toxicity monitoring to identify accrual, and any endpoint problems that might be developing. The Sidney Kimmel Comprehensive Cancer Center Clinical Research Review Committee and Safety Monitoring Committee are responsible for reviewing accrual and safety data for this trial at least twice a year.

12.3.4 Analysis of Exploratory Endpoints

Exploratory correlative studies will be performed to investigate:

- To measure the baseline levels of immune markers including CD8+ T effector cells and CD4+FoxP3+ T regulatory cells, PD-L1 expression and tumor infiltrating lymphocytes (TILs), major histocompatibility complex (MHC) class I and II expression, and natural killer (NK) cell receptors and ligands expression and correlate these variables with treatment response and toxicity.
- To characterize changes in the immune markers described above after treatment
- To identify gene expression changes in malignant tissue after therapy, and complete gene set analysis to elucidate affected pathways
- To measure changes in circulating immune suppressor cells (MDSC, Treg) in peripheral blood by flow cytometry and test for association with response to therapy.

The overarching aim of these exploratory studies is to establish baseline levels and measure changes in immunological pathways after treatment. Little is known about baseline levels for these variables in Cholangiocarcinoma and Pancreatic Adenocarcinoma, let alone changes after treatment, and if the primary and secondary aims are successful, these investigations will establish a biological foundation on which to build in follow-up studies.

Immunological variables will be examined in plots and summary statistics, to characterize distributions, identify outliers and other potential problems in the data. Joint exploratory analysis will identify associations and potential interactions. Variables may be transformed on the basis of these investigations, to reduce skewness, minimize the influence of outliers and/or to regularize relationships between predictors and response for better model fit. Exploratory analysis of the molecular markers, including visualizations and statistical summaries such as hierarchical cluster analysis, heat maps, multidimensional scaling, and principle component analysis will offer important views of the structural characteristics of the expression data.

Response will be described categorically, and associations between response and immunological variables will be characterized, in each cohort, using multivariate logistic regression models, with adjustment for clinical and pathological co-variates that may be associated with response. Estimated effects will be reported with standard errors and confidence intervals.

We will fit empirical Bayes linear models [129] to identify genes for which expression is associated with response to treatment, controlling type I error at an FDR of <10%. Gene set enrichment analysis [130, 131] will be applied to the results to identify immune pathways that are significantly altered after treatment.

13. REFERENCES

1. Kleeff J, Korc M, Apte M, et al. Pancreatic Cancer. Nat Rev Dis Primers. 2016;2:16022
2. Von Hoff, D. D. et al. Increased survival in pancreatic cancer with nab-paclitaxel plus gemcitabine. N Engl J Med.2015;369:1691–1703.
3. Conroy, T. et al. FOLFIRINOX versus gemcitabine for metastatic pancreatic cancer. N Engl J Med. 2011;364: 1817–1825.
4. Wang-Gillam A, Li CP, Bodoky G et al. Nanoliposomal irinotecan with fluorouracil and folinic acid in metastatic pancreatic cancer after previous gemcitabine-based therapy (NAPOLI-1): a global, randomised, open-label, Phase 3 trial. Lancet. 2016;387:545-557
5. Oettle H, Riess H, Stieler JM et al. Second line oxaliplatin, folinic acid, and fluorouracil versus folinic acid and fluorouracil alone for gemcitabine-refractory pancreatic cancer: outcomes from the CONKO-003 trial. J CliN. Oncol. 2014;32:2423–2429.
6. Gill S, Ko YJ, Cripps C, Beaudoin A, et al. PANCREOX: A Randomized Phase III Study of 5-Fluorouracil/Leucovorin With or Without Oxaliplatin for Second-Line Advanced Pancreatic Cancer in Patients Who Have Received Gemcitabine-Based Chemotherapy. J Clin Oncol. 2016;Sep 12. pii: JCO685776. [Epub ahead of print].
7. Ko, A.H., Progress in the treatment of metastatic pancreatic cancer and the search for next opportunities. J Clin Oncol, 2015;33:1779-1786.
8. Siegel RL, Miller KD, Jemal A. Cancer statistics, 2016. A cancer journal for clinicians: CA; 2015.
9. Ebata T, Ercolani G, Alvaro D, et al. Current Status on Cholangiocarcinoma and Gallbladder Cancer. Liver Cancer. 2016;6:59-65.
10. Valle J, Wasan H, Palmer DH, et al. Cisplatin plus gemcitabine versus gemcitabine for biliary tract cancer. N Engl J Med 2010;362:1273-1281.
11. Blechacz B. Cholangiocarcinoma: Current Knowledge and New Developments. Gut and Liver. 2017;11:13-26.
12. Danny N. Khalil DN, Smith E , et al. The future of cancer treatment: immunomodulation, CARs and combination immunotherapy. Nat Rev Clin Oncol. 2016;13:273-90

13. Phan, G. Q. et al. Cancer regression and autoimmunity induced by cytotoxic T lymphocyte associated antigen 4 blockade in patients with metastatic melanoma. Proc.Natl Acad Sci. USA. 2003;100:8372–8377.

14. Parry, R. V. et al. CTLA-4 and PD-1 receptors inhibit T-cell activation by distinct mechanisms. Mol Cell Biol. 2005;25:9543–9553.

15. Okazaki, T. & Honjo T. PD-1 and PD-1 ligands: from discovery to clinical application. Int. Immunol. 2007;19: 813–824.

16. Hodi, F. S. et al. Improved survival with ipilimumab in patients with metastatic melanoma. N Engl J Me. 2010:363:711–723.

17. Robert C. et al. Nivolumab in previously untreated melanoma without BRAF mutation. N Engl J Med. 2014:372: 320–330.

18. Larkin, J. et al. Combined nivolumab and ipilimumab or monotherapy in untreated melanoma. N Engl J Med. 2015;373:23–34.

19. Brahmer, J. et al. Nivolumab versus docetaxel in advanced squamous-cell non-small-cell lung cancer. N Engl J Med. 2015;373:123–134.

20. Topalian, S. L. et al. Safety, activity, and immune correlates of anti-PD-1 antibody in cancer. N Engl J Med. 2012;366:2443–2454.

21. Rizvi NA, Hellmann MD, Snyder A, et al. Cancer immunology. Mutational landscape determines sensitivity to PD-1 blockade in non-small cell lung cancer. Science. 2015;348:124–128.

22. Snyder A, Makarov V, Merghoub T, et al. Genetic basis for clinical response to CTLA-4 blockade in melanoma. N Engl J Med. 2014;371:2189–99.

23. Alexandrov, L. B. et al. Signatures of mutational processes in human cancer. Nature. 2013;500:415–421.

24. Naboush A, Roman CA, Shapira I. Immune checkpoint inhibitors in malignancies with mismatch repair deficiency: a review of the state of the current knowledge. J Investig Med. 2017 Jan 19. doi: 10.1136/jim-2016-000342.

25. Jones, P. A. & Baylin, S. B. The fundamental role of epigenetic events in cancer. Nat Rev Genet.2002;3:415–428.

26. Esteller, M. Epigenetics in cancer. N Engl J Med. 2008;358:1148–1159.

27. Baylin SB. & Jones PA. A decade of exploring the cancer epigenome — biological and translational implications. Nat Rev Cancer. 2011;11:726–734.
28. Dawson MA & Kouzarides T. Cancer epigenetics: from mechanism to therapy. Cell. 2012;150:12–27.
29. Azad N, Zahnow CA, Rudin CM, Baylin SB. The future of epigenetic therapy in solid tumours--lessons from the past. Nat Rev Clin Oncol. 2013;10:256-266.
30. Doi A. et al. Differential methylation of tissue- and cancer-specific CpG island shores distinguishes human induced pluripotent stem cells, embryonic stem cells and fibroblasts. Nat Genet. 2009;41:1350–1353.
31. Waddell N, Pajic M, Patch AM, et al. Whole genomes redefine the mutational landscape of pancreatic cancer. Nature 2015;518:495–501.
32. Hashimoto, H., Vertino, P. M. & Cheng, X. Molecular coupling of DNA methylation and histone methylation. Epigenomics. 2010;2:657–669.
33. Jiao Y, Pawlik TM, Anders RA, et al. Exome sequencing identifies frequent inactivating mutations in BAP1, ARID1A and PBRM1 in intrahepatic cholangiocarcinomas. Nat Genet 2013;45:1470-1473.
34. Bolden JE, Peart MJ, Johnstone RW. Anticancer activities of histone deacetylase inhibitors. Nat Rev Drug Discov 2006;5:769–84.
35. Khan O, La Thangue NB. HDAC inhibitors in cancer biology: Emerging mechanisms and clinical applications. Immunol Cell Biol 2012;90:85–94.
36. Kim K, Skora AD, Li Z, et al. Eradication of metastatic mouse cancers resistant to immune checkpoint blockade by suppression of myeloid-derived cells. Proc Natl Acad Sci U S A. 2014;111:11774-11779.
37. Robert C. & Rassool FV. HDAC inhibitors: roles of DNA damage and repair. Adv Cancer Res. 2012;116:87–129.
38. Popovic R & Licht JD. Emerging epigenetic targets and therapies in cancer medicine. Cancer Discov. 2012;2: 405–413.
39. Shen L, Ciesielski M, Ramakrishnan S,et al. Class I Histone Deacetylase Inhibitor Entinostat Suppresses Regulatory T Cells and Enhances Immunotherapies in Renal and Prostate Cancer Models. PLoS One. 2012;7: e30815.
40. Sriraksa R, Limpaiboon T. Histone deacetylases and their inhibitors as potential therapeutic drugs for cholangiocarcinoma - cell line findings. Asian Pac J Cancer Prev. 2013;14:25032508.

41. Baradari V, Höpfner M, Huether A, et al. Histone deacetylase inhibitor MS-275 alone or combined with bortezomib or sorafenib exhibits strong antiproliferative action in human cholangiocarcinoma cells. World J Gastroenterol. 2007;13:4458–4466.
42. R Pili, B Salumbides, M Zhao, et al. Phase I study of the histone deacetylase inhibitor entinostat in combination with 13-cis retinoic acid in patients with solid tumours. Br J Cancer. 2012;106:77–84.
43. Alexander Arlt & Heiner Schäfer. Investigational histone deacetylase inhibitors for treating pancreatic adenocarcinoma Expert opinion on investigational drugs. 2016;25:1251–1254.
44. Hessmann E, Johnsen SA, Siveke JT, et al. Epigenetic treatment of pancreatic cancer: is there a therapeutic perspective on the horizon? Gut 2017;66:168–179.
45. Koutsounas I, Giaginis C, Theocharis S. Histone deacetylase inhibitors and pancreatic cancer: are there any promising clinical trials? World J Gastroenterol. 2013;19:1173–1181.
46. Jones SF, Bendell JC, Infante JR, et al. A phase I study of panobinostat in combination with gemcitabine in the treatment of solid tumors. Clin Adv Hematol Oncol. 2011;9:225-230.
47. Jones SF, Infante JR, Spigel DR, et al. Phase 1 Results From a Study of Romidepsin in Combination With Gemcitabine in Patients With Advanced Solid Tumors. Cancer Investigation. 2012;30:6
48. Richards DA, Boehm KA, Waterhouse DM, et al. Gemcitabine plus CI-994 offers no advantage over gemcitabine alone in the treatment of patients with advanced pancreatic cancer: results of a phase II randomized, double-blind, placebo-controlled, multicenter study. Ann Oncol. 2006;17:1096–1102
49. Kleeff, J. et al. Pancreatic cancer microenvironment. Int. J. Cancer. 2007;121:699–705.
50. Wang, L. M. et al. The prognostic role of desmoplastic stroma in pancreatic ductal adenocarcinoma. Oncotarget. 2016;7:4183–4194.
51. Amedei A, Niccolai E & Prisco D. Pancreatic cancer: role of the immune system in cancer progression and vaccine-based immunotherapy. Hum. Vaccin. Immunother. 2014;10:3354–3368.
52. Le DT. et al. Safety and survival with GVAX pancreas prime and Listeria monocytogenesexpressing mesothelin (CRS-207) boost vaccines for metastatic pancreatic cancer. J Clin Oncol. 2015;33:1325–1333.
53. Khaled, Y. S., Wright, K., Melcher, A. & Jayne, D. Anti-cancer effects of oncolytic viral therapy combined with photodynamic therapy in human pancreatic cancer cell lines. Lancet. 2015;385, S56.

54. Soares, K. C. et al. PD-1/PD-L1 blockade together with vaccine therapy facilitates effector T-cell infiltration into pancreatic tumors. J. Immunother 2015;38:1–11.

55. Brahmer, J. R. et al. Safety and activity of anti-PD-L1 antibody in patients with advanced cancer. N Engl J Med. 2012;366:2455–2465.

56. Feig C. et al. Targeting CXCL12 from FAP-expressing carcinoma-associated fibroblasts synergizes with anti-PD-L1 immunotherapy in pancreatic cancer. Proc Natl Acad Sci USA. 2013;110:20212–20217.

57. Lutz, E. R. et al. Immunotherapy converts nonimmunogenic pancreatic tumors into immunogenic foci of immune regulation. Cancer Immunol Res. 2014;2:616–631.

58. Beatty, G. L. et al. CD40 agonists alter tumor stroma and show efficacy against pancreatic carcinoma in mice and humans. Science. 2011;331:1612–1616.

59. Beatty, G. L. et al. Exclusion of T cells from pancreatic carcinomas in mice is regulated by Ly6Clow F4/80+ extratumoral macrophages. Gastroenterology. 2015;149:201–210.

60. Silverman BR and Jiaqi Shi J. Alterations of Epigenetic Regulators in Pancreatic Cancer and Their Clinical Implications. Int J Mol Sci 2016;17:2138-2145

61. Jones PA & Baylin SB. The epigenomics of cancer. Cell. 2007;128:683–692.

62. Baylin SB & Jones PA. A decade of exploring the cancer epigenome- biological and translational implications. Nat Rev Cancer. 2011;11:726–734.

63. Witkiewicz AK, McMillan EA, Balaji U, et al. Whole-exome sequencing of pancreatic cancer defines genetic diversity and therapeutic targets. Nat Commun. 2015;6:6744–6755.

64. Ougolkov AV, Bilim VN, Billadeau DD. Regulation of pancreatic tumor cell proliferation and chemoresistance by the histone methyltransferase enhancer of ZESTE homologue 2. Clin Cancer Res. 2008;14:6790–6796.

65. Sigalotti L, Fratta E, Coral S, et al. Epigenetic drugs as immunomodulators for combination therapies in solid tumors. Pharmacol Ther. 2014;142:339–350.

66. Suleiman Y, Coppola D, Zibadi S, et al. Prognostic value of tumor-infiltrating lymphocytes (TILs) and expression of PD-L1 in cholangiocarcinoma.J Clin Oncol. 2015; (suppl 3; abstr 294)

67. Sabbatino F, Villani V, Yearley JH, et al. PD-L1 and HLA class I antigen expression and clinical course of the disease in intrahepatic cholangiocarcinoma. Clin Cancer Res. 2016;22:470–478.
68. Gani F, Nagarajan N, Kim Y,et al. Program Death 1 Immune Checkpoint and Tumor Microenvironment: Implications for Patients With Intrahepatic Cholangiocarcinoma. Ann Surg Oncol. 2016;23:2610–2617.
69. Tran E, Turcotte S, Gros A, et al. Cancer Immunotherapy Based on Mutation-Specific CD4+ T Cells in a Patient with Epithelial Cancer. Science. 2014;344:641-645.
70. Le DT, Uram JN, Wang H, et al. PD-1 blockade in tumors with mismatch-repair deficiency. N Engl J Med. 2015;372:2509–2520.
71. Bang YJ, Doi T, Braud FD, et al. Safety and efficacy of pembrolizumab (MK-3475) in patients (pts) with advanced biliary tract cancer: interim results of KEYNOTE-028. Eur J Cancer. 2015;51:S112.
72. Wang P, Dong Q, Zhang C, et al. Mutations in isocitrate dehydrogenase 1 and 2 occur frequently in intrahepatic cholangiocarcinomas and share hypermethylation targets with glioblastomas. Oncogene. 2013;32:3091–100.
73. Apurva JA, Kwong NK, Javle M. Genomic Profiling of Biliary Tract Cancers and Implications for Clinical Practice Curr Treat Options in Oncol. 2016:17:58.
74. Hess-Stumpp H. Histone deacetylase inhibitors and cancer: from cell biology to the clinic. Eur J Cell Biol. 2005;84:109-121.
75. Abraham J, Nuñez-Álvarez Y, Hettmer S, et al. Lineage of origin in rhabdomyosarcoma informs pharmacological response. Genes & Dev. 2014;28:1578-1591.
76. Qian, D.Z.; Kachhap, S.K.; Collis, S.J.; Verheul, H.M.; Carducci, M.A.; Atadja, P.; Pili, R. Class II histone deacetylases are associated with VHL-independent regulation of hypoxiainducible factor 1 alpha. Cancer Res. 2006;66:8814–8821.
77. Schech A, Kazi A, Yu S, Shah P. Histone Deacetylase Inhibitor Entinostat Inhibits TumorInitiating Cells in Triple-Negative Breast Cancer Cells. Mol Cancer Ther. 2015;14:18481857.
78. Investigator Brochure: Etinostat, SNDX-27. 2014.
79. Hodges-Gallagher L, Valentine CD, Bader SE, Kushner PJ. Inhibition of histone deacetylase enhances the anti-proliferative action of antiestrogens on breast cancer cells and blocks tamoxifen-induced proliferation of uterine cells. Breast Cancer Res Treat 2007;105:297-309.

80. Acharya MR, Karp JE, Sausville EA et al. Factors affecting the pharmacokinetic profile of MS-275, a novel histone deacetylase inhibitor, in patients with cancer. Invest New Drugs 2006;24:367-375.
81. Ordentlich P. Pharmacodynamic analysis of ENCORE 301, a placebo-controlled, randomized phase 2 study of exemestane with and without entinostat in postmenopausal ER+ breast cancer patients demonstrates an association of lysine hyperacetylation with clinical outcome. AACR Molecular Targets Conference 2011
82. Investigator Brochure: Nivolumab, BMS-936558, MDX1106. 2014.
83. Sznol M, Kluger HM, Hodi FS et al. Survival and long-term follow-up of safety and response in patients (pts) with advanced melanoma (MEL) in a phase I trial of nivolumab (anti-PD-1; BMS-936558; ONO-4538). J Clin Oncol 2013;31:ACRA9006.
84. Brahmer JR, Horn L, Antonia SJ et al. Survival and long-term follow-up of the phase I trial of nivolumab (Anti-PD-1; BMS-936558; ONO-4538) in patients (pts) with previously treated advanced non-small cell lung cancer. J Clin Oncol 2013;31:A8030.
85. Drake CG, McDermott DF, Sznol M et al. Survival, safety, and response duration results of nivolumab (Anti-PD-1; BMS-936558; ONO-4538) in a phase I trial in patients with previously treated metastatic renal cell carcinoma (mRCC): Long-term patient follow-up. J Clin Oncol 2013;31:A4514.
86. Powles, T. et al. MPDL3280A (anti‑PD‑L1) treatment leads to clinical activity in metastatic bladder cancer. Nature. 2014;515:558–562.
87. Ansell, S. M. et al. PD‑1 blockade with nivolumab in relapsed or refractory Hodgkin’s lymphoma. N Engl J Med. 2014;372:311–319.
88. Woo SR, Turnis ME, Goldberg MV et al. Immune inhibitory molecules LAG-3 and PD-1 synergistically regulate T-cell function to promote tumoral immune escape. Cancer Res. 2012;72:917-927.
89. Taube JM, Anders RA, Young GD et al. Colocalization of inflammatory response with B7h1 expression in human melanocytic lesions supports an adaptive resistance mechanism of immune escape. Sci Transl Med. 2012; 4:127ra137.
90. Ohigashi Y, Sho M, Yamada Y et al. Clinical significance of programmed death-1 ligand1 and programmed death-1 ligand-2 expression in human esophageal cancer. Clin Cancer Res. 2005; 11: 2947-2953.

91. Wu C, Zhu Y, Jiang J et al. Immunohistochemical localization of programmed death-1 ligand-1 (PD-L1) in gastric carcinoma and its clinical significance. Acta Histochem. 2006;108:19-24.
92. Thompson RH, Kuntz SM, Leibovich BC et al. Tumor B7-H1 is associated with poor prognosis in renal cell carcinoma patients with long-term follow-up. Cancer Res. 2006;66:3381-3385.
93. Nomi T, Sho M, Akahori T et al. Clinical significance and therapeutic potential of the programmed death-1 ligand/programmed death-1 pathway in human pancreatic cancer. Clin Cancer Res. 2007;13:2151-2157.
94. Topalian SL, Hodi FS, Brahmer JR, et al. Safety, Activity, and Immune Correlates of Anti–PD-1 Antibody in Cancer. N Engl J Med. 2012;366:2443-2454.
95. Tough DF, Tak PP, Tarakhovsky A, et al. Epigenetic drug discovery: breaking through the immune barrier. Nature Reviews Drug Discovery. 2016;15:835–853.
96. Juergens, R. A. et al. Combination epigenetic therapy has efficacy in patients with refractory advanced non-small cell lung cancer. Cancer Discov. 2011;1:598–607.
97. Vanneman M and Dranoff G. Combining Immunotherapy and Targeted Therapies in Cancer Treatment. Nat Rev Cancer. 2012;12:237–251.
98. Sharma, P. and Allison, J. The future of immune checkpoint therapy. Science 2015;348:56–61.
99. Mahoney KM, Rennert PD, Freeman GJ. Combination cancer immunotherapy and new immunomodulatory targets. Nat Rev Drug Discov. 2015;14:561-584.
100. Marvel D and Gabrilovich DI. Myeloid-derived suppressor cells in the tumor microenvironment: expect the unexpected. J Clin Invest. 2015;125:3356-3364.
101. Solito S, Falisi E, Diaz-Montero CM, Doni A, Pinton L, Rosato A, Francescato S, Basso G, Zanovello P, Onicescu G, et al. A human promyelocytic-like population is responsible for the immune suppression mediated by myeloid-derived suppressor cells. Blood. 2011;118:2254–2265.
102. Markowitz J, Wesolowski,R, Papenfuss T, et al. Myeloid Derived Suppressor Cells in Breast Cancer. Breast Cancer Res Treat. 2013;140:13–21.
103. Woods DM, Sodré AL, Villagra A, et al. HDAC Inhibition Upregulates PD-1 Ligands in Melanoma and Augments Immunotherapy with PD-1 Blockade. Cancer Immunol Res. 2015;3:1375-1385.

104. Nomi T, Sho M, Akahori T et al. Clinical significance and therapeutic potential of the programmed death-1 ligand/programmed death-1 pathway in human pancreatic cancer. Clin Cancer Res 2007;13:2151-2157.
105. Bates GJ, et al. Quantification of regulatory T cells enables the identification of high-risk breast cancer patients and those at risk of late relapse. J Clin Oncol. 2006;24:5373–5380.
106. Fu J, et al. Increased regulatory T cells correlate with CD8 T-cell impairment and poor survival in hepatocellular carcinoma patients. Gastroenterology. 2007;132:2328–2339.
107. Quail DF and Joyce JA. Microenvironmental regulation of tumor progression and metastasis. Nat Med. 2013; 19:1423–1437.
108. Leoni, F. et al. The antitumor histone deacetylase inhibitor suberoylanilide hydroxamic acid exhibits antiinflammatory properties via suppression of cytokines. Proc Natl Acad Sci USA. 2002;99:2995–3000.
109. Yang H, Bueso-Ramos C, DiNardo C, et al. Expression of PD-L1, PD-L2, PD-1 and CTLA4 in myelodysplastic syndromes is enhanced by treatment with hypomethylating agents. Leukemia. 2014;28:1280–1288.
110. Abdelfatah E, Kerner Z, Nanda N, and Nita Ahuja. Epigenetic therapy in gastrointestinal cancer: the right combination. Therap Adv Gastroenterol. 2016;9:560–579.
111. Li H, Chiappinelli KB, Guzzetta AA, et al. Immune regulation by low doses of the DNA methyltransferase inhibitor 5-azacitidine in common human epithelial cancers. Oncotarget. 2014;5:587–598
112. Zitvogel L, Tesniere A, Kroemer G. Cancer despite immunosurveillance: immunoselection and immunosubversion. Nat Rev Immunol 2006;6:715-727.
113. Tumeh PC, Harview CL, Yearley JH et al. PD-1 blockade induces responses by inhibiting adaptive immune resistance. Nature 2014;515:568-571.
114. Llosa NJ, Cruise M, Tam A et al. The vigorous immune microenvironment of microsatellite instable colon cancer is balanced by multiple counter-inhibitory checkpoints. Cancer Discov 2015;5:43-51.
115. Dulaimi E, Hillinck J, de C, II et al. Tumor suppressor gene promoter hypermethylation in serum of breast cancer patients. Clin Cancer Res 2004;10:6189-6193.
116. Tomita Y, Lee MJ, Lee S, et al. The interplay of epigenetic therapy and immunity in locally recurrent or metastatic estrogen receptor-positive breast cancer: Correlative analysis of ENCORE 301, a randomized, placebo-controlled phase II trial of exemestane with or without entinostat. Oncoimmunology. 2016;5: e1219008.
117. https://clinicaltrials.gov/ct2/show/NCT02453620

118. https://clinicaltrials.gov/ct2/show/NCT02437136
119. Zhu S, Denman CJ, Cobanoglu ZS, et al. The narrow-spectrum HDAC inhibitor entinostat enhances NKG2D expression without NK cell toxicity, leading to enhanced recognition of cancer cells. Pharmaceutical research. 2015;32:779-792.
120. Vaccari M, Boasso A, Fenizia C et al. Fatal pancreatitis in simian immunodeficiency virus SIV(mac251)-infected macaques treated with 2',3'-dideoxyinosine and stavudine following cytotoxic-T-lymphocyte-associated antigen 4 and indoleamine 2,3-dioxygenase blockade. J Virol 2012;86:108-113
121. Johnson DB, Balko JM, Compton ML, et al. Fulminant Myocarditis with Combination Immune Checkpoint Blockade. N Engl J Med. 20163;375:1749-1755.
122. Hauschild A, Trefzer U, Garbe C, Kaehler KC, Ugurel S, Kiecker F, et al. Multicenter phase II trial of the histone deacetylase inhibitor pyridylmethyl-N-{4-[(2-aminophenyl)carbamoyl]-benzyl}-carbamate in pretreated metastatic melanoma. Melanoma Res 2008;18:274-8.
123. Therasse P, Arbuck SG, Eisenhauer EA, et al. New Guidelines to Evaluate the Response to Treatment in Solid Tumors. J Natl Cancer Inst. 2000;9:2205-2216.
124. Dowlati A, et al. Sequential Tumor Biopsies in Early Phase Clinical Trials of Anticancer Agents for Pharmacodynamic Evaluation. Clin Cancer Res 2001; 7: 2971-76.
125. Gupta S et al. Quality Improvement Guidelines for Percutaneous Needle Biopsy. J Vasc Interv Radiol 2010; 21:969-75
126. Lee J; Hays JL, Noonan Am,et al. Feasibility and Safety of Sequential Research-Related Tumor Core Biopsies in Clinical Trials. Cancer.;119:1357-64, 2013
127. Lutz ER, Wu AA, Bigelow E, et al. Immunotherapy Converts Nonimmunogenic Pancreatic Tumors into Immunogenic Foci of Immune Regulation Cancer Immunol Res. 2014;2:616632.
128. Bigelow E, Bever KM, Xu H et al. Immunohistochemical staining of B7-H1 (PD-L1) on paraffin-embedded slides of pancreatic adenocarcinoma tissue. J Vis Exp 2013(71), e4059, doi:10.3791/4059.
129. Ritchie ME, Phipson B, Wu D, et al. limma powers differential expression analyses for RNA-sequencing and microarray studies. Nucleic Acids Res. 2015:43:e47
130. Subramanian A, Tamayo P, Mootha VK, et al. Gene set enrichment analysis: a knowledgebased approach for interpreting genome-wide expression profiles. Proc Natl Acad Sci U S A. 2005;102:15545-50.

131. Mootha VK, Lindgren CM, Eriksson KF et al. PGC-1alpha-responsive genes involved in oxidative phosphorylation are coordinately downregulated in human diabetes. Nat Genet. 2003 Jul;34(3):267-73.

14. APPENDICES

Appendix A: Eastern Cooperative Oncology Group Performance Status Scale, with Equivalent Karnofsky Performance Status Scores

ECOG[1]		Karnofsky[2]	
**Score**	**Criterion**	**%**	**Criterion**
0	Normal activity	100	Normal; no complaints; no evidence of disease
		90	Able to carry on normal activity; minor signs or symptoms of disease
1	Symptoms but ambulatory	80	Normal activity with effort; some signs or symptoms of disease
		70	Cares for self; unable to carry on normal activity or do active work
2	In bed <50% of time	60	Requires occasional assistance but is able to care for most of his/her needs
		50	Requires considerable assistance and frequent medical care
3	In bed >50% of time	40	Disabled, requires special care and assistance
		30	Severely disabled; hospitalization is indicated though death is not imminent
4	100% bedridden	20	Very sick; hospitalization is necessary
		10	Moribund; fatal processes progressing rapidly
5	Dead	0	Dead

[1]Oken MM, Creech RH, Tormey DC, Horton J, Davis TE, McFadden ET, Carbone PP. Toxicity and response criteria of the Eastern Cooperative Oncology Group. Am J Clin Oncol. 1982;5:649-655.

2 Mor V, Laliberte L, Morris JN, Wiemann M. The Karnofsky Performance Status Scale: an examination of its reliability and validity in a research setting. Cancer. 1984;53:2002-2007.

Appendix B: Patient Drug Information Handout and Wallet Card

Information for Patients, Their Caregivers, and Non-Study Healthcare Team on Possible Interactions with Other Drugs and Herbal Supplements

[Note to site: This appendix consists of an "information sheet" to be handed to the patient at the time of enrollment. Update the "patient name" and study doctor's/team's contact information prior to giving to the patient.]

The patient, ____________________________, is enrolled on a clinical trial using the experimental study drugs: entinostat and nivolumab. This clinical trial is sponsored by the National Cancer Institute. This form is addressed to the patient, but includes important information for others who care for this patient.

These are the things that you as a healthcare provider need to know:

Entinostat and nivolumab are not known or expected to interact with certain enzymes in the body, the heart's electrical activity, or with other medications; however, there is the possibility of interactions. For instance, entinostat may interact with other drugs that are processed by your liver.

To the patient: Take this paper with you to your medical appointments and keep the attached information card in your wallet.

Entinostat and nivolumab may interact with other drugs which can cause side effects. For this reason, it is very important to tell your study doctors of any medicines you are taking before you enroll onto this clinical trial. It is also very important to tell your doctors if you stop taking any regular medicines, or if you start taking a new medicine while you take part in this study. When you talk about your current medications with your doctors, include medicine you buy without a prescription (over-the-counter remedy), or any herbal supplements such as St. John's Wort. It is helpful to bring your medication bottles or an updated medication list with you.

Many health care providers can write prescriptions. You must tell all of your health care providers (doctors, physician assistants, nurse practitioners, pharmacists) you are taking part in a clinical trial.

These are the things that you and they need to know:

Before you enroll onto the clinical trial, your study doctor will work with you and your regular health care providers, as needed, to review any medicines and herbal supplements that you are taking or may start during the study.

- Please be very careful! Over-the-counter drugs (including herbal supplements) may contain ingredients that could interact with your study drug. Speak to your doctors or pharmacist to determine if there could be any side effects.
- Your regular health care provider should check a frequently updated medical reference or call your study doctor before prescribing any new medicine or discontinuing any medicine. Your study doctor's name is…___________________________________

  and he or she can be contacted at …_________________________________.

**STUDY DRUG INFORMATION WALLET CARD**<br>You are enrolled on a clinical trial using the experimental study drugs entinostat and nivolumab . This clinical trial is sponsored by the NCI. **Entinostat and nivolumab** may interact with other drugs.<br>Because of this, it is very important to:<br>➢ Tell your doctors if you stop taking any medicines or if you start taking any new medicines.<br>➢ Tell all of your health care providers (doctors, physician assistants, nurse practitioners, or pharmacists) that you are taking part in a clinical trial.<br>➢ Check with your doctor or pharmacist whenever you need to use an over-the-counter medicine or herbal supplement.	**Entinostat and nivolumab** must be used very carefully with other medicines.<br>➢ Before you enroll onto the clinical trial, your study doctor will work with you and your regular health care providers, as needed, to review any medicines and herbal supplements that you are taking or may start during the study.<br>➢ Before prescribing new medicines, your regular health care providers should go to a frequently-updated medical reference for a list of drugs to avoid, or contact your study doctor.<br>➢ Your study doctor's name is _____________________________ and can be contacted at _________________________________.

Appendix C: Entinostat Drug Diary

Subject ID: ______________, Subject Initials: ______________, Cycle #: _____

Directions:

- Use this diary to record each dose of entinostat that you take.
- You should take your scheduled dose of each pill. If you miss a dose and it is within 24 hours of when it should have been taken, you should take the dose. If it has been more than 24 hours of when you should have taken the dose, call the study staff. Do not "double-up" to try and make-up missed doses.
- Entinostat should be taken on an empty stomach, at least 1 hour before or 2 hours after a meal/snack.
- Please bring the empty bottle or any leftover tablets and your pill calendar to your next clinic visit.
- If you develop any side effects, please record them and anything you would like to tell the doctor in the space provided.

Assigned entinostat dose: _____ mg　　Number of pills to take at each dose: _____
Start date of calendar: _______________

Day	Date	Entinostat		Comments
	(Month / Day / Year)	# pills	Time	(such as side effects, symptoms, other medications)
-14				
-7				
Additional information (if needed):				

Week	Date	Entinostat		Comments
	(Month / Day / Year)	# pills	Time	(such as side effects, symptoms, other medications)
1				
2				
3				
4				
Additional information (if needed):				

Completed by: ______________________________ Date: ______________
Signature of Participant

Reviewed by: ______________________________ Date: ______________

Appendix D: Management Algorithms for Nivolumab -Related Adverse Events

Adverse Event Management Algorithms:

GI
Renal
Pulmonary
Hepatic
Endocrinopathy
Skin
Neurological

[redacted]

[redacted]

[redacted]

[redacted]

[redacted]

[redacted]

[redacted]

Appendix E: Concomitant Medications to be Avoided

Examples of sensitive *in vivo* CYP substrates and CYP substrates with narrow therapeutic range are summarized in *Table 14.1*

Table 14.1 Examples of substrates that may be affected by entinostat

CYP Enzymes	Substrates with narrow therapeutic range[1]
CYP1A2	Theophylline, tizanidine
CYP2C8	Paclitaxel
CYP3A[2]	Alfentanil, astemizole[3], cisapride[4], cyclosporine, ihydroergotamine, ergotamine, fentanyl, pimozide, quinidine, sirolimus, tacrolimus, terfenadine[4]

[1] *CYP substrates with narrow therapeutic range refers to drugs whose exposure-response relationship indicates that small increases in their exposure levels by the concomitant use of CYP inhibitors may lead to serious safety concerns (e.g., Torsades de Pointes).*
[2] *Because a number of CYP3A substrates (e.g., darunavir, maraviroc) are also substrates of P-gp, the observed increase in exposure could be due to inhibition of both CYP3A and P-gp.*
[3] *Withdrawn from the United States market because of safety reasons.*

Refer to *Table 14.2* for examples of transporter inhibitors and inducers.

Table 14.2 P-gp Inhibitors and Inducers

	Inhibitor	Inducer
P-gp, MDR1	Amiodarone, azithromycin, captopril, carvedilol, clarithromycin, conivaptan, diltiazem, dronedarone, felodipine, lopinavir,quercetin, ranolazine,ticagrelor, ritonavir, cyclosporine, verapamil erythromycin, ketoconazole, itraconazole, quinidine	Avasimibe, carbamazepine, phenytoin, rifampin, St John's Wort, tipranavir/ritonavir

Appendix F: Serious Adverse Event Reporting Form

**Protocol Title:**	A Phase 2 Clinical Trial of Entinostat in Combination with Nivolumab for Patients with Previously Treated Unresectable or Metastatic Cholangiocarcinoma and Pancreatic Adenocarcinoma		
**Protocol Number:**<br>J1798<br>CA209-9L6<br>SNDX-275-0205	**Signature of PI:**	**Principal Investigator:**	**Date:**
**Report Type:**<br>☐**Initial**<br>☐**Follow-up**<br>☐**Final Follow-up**<br>☐**Death**<br>☐**Addendum to:**	**Serious Criteria (check all that apply):**<br>☐Death<br>☐Life-threatening<br>☐ Hospitalization or Elongation of Existing Hospitalization<br>☐ Persistent or Significant Disability<br>☐ Congenital Anomaly<br>☐ Other Important Medical Event<br>☐ Cancer<br>☐ Overdose	**Hospital Admission Date:**<br><br>**Hospital Discharge Date:**	**Date Event Discovered:**

**Section A: Subject Information**			
**Subject ID:**	**Subject Initial:**	**DOB:**	**Subject Gender:**<br>☐**Male**<br>☐**Female**

**Section B: Event Information**			
**Event diagnosis or symptoms:**	**Date of First Dose:**		**Action taken with the study drug:**<br>☐**None**<br>☐**Interrupted**<br>☐**Discontinued**<br>☐**Delayed**
	**Entinostat**	**Nivolumab**	
	**Date of Last Dose Prior to Event:**		
	**Entinostat**	**Nivolumab**	
	**Number of Total Doses:**		
	**Entinostat**	**Nivolumab**	

**Event Onset Date:**			**Event End Date:**
**Relationship to:**	**Entinostat**	**Nivolumab**	**Underlying Disease**
**Unrelated**	☐	☐	☐
**Related**	☐	☐	☐

Section C: Brief Description of the Event:

Section D: Relevant Medical History

Section E: Concomitant Drug (Not related to SAE)

Name of the Drug	Start Date	Stop Date	Route	Dose	Frequency

Section F: Comments

Additional Documents: ☐ Please specify

[redacted]
