# Supplementary material for: Entinostat in combination with nivolumab in metastatic pancreatic ductal adenocarcinoma: a phase 2 clinical trial
Source: Nat Commun. 2024 Nov 12;15:9801. doi: 10.1038/s41467-024-52528-7 (PMC11557583; doi:10.1038/s41467-024-52528-7)
Supplement: Supplementary file 3 — Description of Additional Supplementary Files [file 41467_2024_52528_MOESM3_ESM.pdf]

Description of Additional Supplementary Files

Supplementary Data 1 - mIHC Cell Densities

Supplementary Data 2 - mIHC Wilcox pValues Densities

Supplementary Data 3 - mIHC Percentages

Supplementary Data 4 - mIHC Wilcox pValues Percentage

Supplementary Data 5 - mIHC Ratios

Supplementary Data 6 - CyTOF Cell Type Annotation

Supplementary Data 7 - CyTOF Myeloid Proportions

Supplementary Data 8 - CyTOF Myeloid Wilcox pValues Proportions

Supplementary Data 9 - CyTOF Myeloid and B cells Markers Expression

Supplementary Data 10 - CyTOF Myeloid and B cells Wilcox pValues Markers Expression

Supplementary Data 11 - Cytokines Panel

Supplementary Data 12 - Cytokines values at different timepoints

Supplementary Data 13 - Cytokine Wilcox pValues

Supplementary Data 14 - CyTOF Lymphoid cells Proportions

Supplementary Data 15 - CyTOF Lymphoid populations Wilcox pValues Proportions

Supplementary Data 16 - Top Differential Expressed Genes between Baseline and C1D1

Supplementary Data 17 - Top Differential Expressed Genes between Baseline and C2D1

Supplementary Data 18 - Top Differential Expressed Genes Between C1D1 and C2D1

Supplementary Data 19 - Hallmark Pathway Analysis Baseline versus C2D1

Supplementary Data 20 - Hallmark Pathway Analysis C1D1 versus C2D1

Supplementary Data 21 - Hallmark Pathways Analysis Baseline versus C1D1

Supplementary Data 22 - CyTOF panel design for myeloid cell populations

Supplementary Data 23 - CyTOF panel design for lymphoid cell populations

Supplementary Data 24 - Antibody-metal conjugation kits used in CyTOF panel designs

Supplementary Data 25 - RNAseq
