# Supplementary material for: Entinostat in combination with nivolumab in metastatic pancreatic ductal adenocarcinoma: a phase 2 clinical trial
Source: Nat Commun. 2024 Nov 12;15:9801. doi: 10.1038/s41467-024-52528-7 (PMC11557583; doi:10.1038/s41467-024-52528-7)
Supplement: Supplementary file 29 — Reporting Summary [file 41467_2024_52528_MOESM29_ESM.pdf]

nature portfolio

Corresponding author(s): Nilofer Azad

Last updated by author(s): 07/22/2024

# Reporting Summary

Nature Portfolio wishes to improve the reproducibility of the work that we publish. This form provides structure for consistency and transparency in reporting. For further information on Nature Portfolio policies, see our Editorial Policies and the Editorial Policy Checklist.

## Statistics

For all statistical analyses, confirm that the following items are present in the figure legend, table legend, main text, or Methods section.

| n/a | Confirmed | |
|---|---|---|
| ☐ | ☒ | The exact sample size (*n*) for each experimental group/condition, given as a discrete number and unit of measurement |
| ☐ | ☒ | A statement on whether measurements were taken from distinct samples or whether the same sample was measured repeatedly |
| ☐ | ☒ | The statistical test(s) used AND whether they are one- or two-sided<br>*Only common tests should be described solely by name; describe more complex techniques in the Methods section.* |
| ☐ | ☒ | A description of all covariates tested |
| ☐ | ☒ | A description of any assumptions or corrections, such as tests of normality and adjustment for multiple comparisons |
| ☐ | ☒ | A full description of the statistical parameters including central tendency (e.g. means) or other basic estimates (e.g. regression coefficient) AND variation (e.g. standard deviation) or associated estimates of uncertainty (e.g. confidence intervals) |
| ☐ | ☒ | For null hypothesis testing, the test statistic (e.g. *F*, *t*, *r*) with confidence intervals, effect sizes, degrees of freedom and *P* value noted<br>*Give P values as exact values whenever suitable.* |
| ☒ | ☐ | For Bayesian analysis, information on the choice of priors and Markov chain Monte Carlo settings |
| ☒ | ☐ | For hierarchical and complex designs, identification of the appropriate level for tests and full reporting of outcomes |
| ☒ | ☐ | Estimates of effect sizes (e.g. Cohen's *d*, Pearson's *r*), indicating how they were calculated |

*Our web collection on statistics for biologists contains articles on many of the points above.*

## Software and code

Policy information about availability of computer code

Data collection

For all CyTOF analyses, all data were acquired using Helios™ at the Sidney Kimmel Comprehensive Cancer Center in Baltimore, Maryland. For preprocessing of CyTOF data, randomization, bead-based normalization, and bead removal of data were performed in CyTOF software (Fluidigm®) v6.7 followed by gating of cell events (rhodium vs. cell length signal) that are viable (106Pd vs. 108Pd) in FlowJo (BD Biosciences) v10.5. Individual samples were debarcoded by hierarchal gating (three positive and two negative CD45 axes) and exported as separate FCS files for analysis.

For RNA sequencing analysis, each cryovial containing a single core biopsy sample in RNAlaterTM was placed in the refrigerator (4ºC) for ≥16-24 hours after the biopsy procedure. After ≥16-24 hours, the RNAlaterTM vials were stored at -70oC or below for storage. For RNA sequencing experiments, RNA libraries were generated using the TruSeq Stranded Total RNA Library kit according to manufacturer instructions (Illumina; San Diego, CA). Quality and quantity of the resulting cDNA were monitored using the Bioanalyzer High Sensitivity kit (Agilent). mRNA libraries were sequenced on an Illumina Novaseq 6000 instrument using 150bp paired-end dual indexed reads and 1% of PhiX control on an S4 flowcell. Depth of coverage was targeted to a total of 50 million reads per library. Illumina's CASAVA (v1.8.4) with default parameters was used to generate FASTQ files with reads. Then the reads were trimmed with trimgalore (v0.6.3) with default parameters and aligned to human genome (hg38) and quantified with the rsem algorithm (v1.3.0)58. The RSEM expected counts were used for gene level expression.

The concentrations of cytokines and chemokines were assessed by the Sidney Kimmel Comprehensive Cancer Center (SKCCC) Immune Monitoring Core using Luminex bead-based immunoassays. The Bioplex 200 platform (Biorad, Hercules CA) was used to determine the concentration of multiple target proteins in serum/plasma specimens following Immune Monitoring Core SOPs, and concentrations were determined using 5 parameter log curve fits (using Bioplex Manager 6.0) with vendor-provided standards and quality controls.

Data analysis

For all CyTOF analyses, a computational pipeline based on diffcyt2 was employed using R. For unsupervised clustering, the FlowSOM3

algorithm was used to identify meta clusters that were then annotated and merged into final cell subtypes based on published literature. Analysis of RNA sequencing data was performed in R. For differential expression analysis, we completed a paired analysis between time points using the DESeq2 package (v1.32.0), including the patient as a covariate in our model, along with our comparison of interest. Estimated fold changes are shrunk with ashr using lfcShrink to account for the variation in the samples in this dataset. Gene set statistics were run with fgsea using MSigDb61 v7.4.1 pathways annotated in the HALLMARK databases.

For manuscripts utilizing custom algorithms or software that are central to the research but not yet described in published literature, software must be made available to editors and reviewers. We strongly encourage code deposition in a community repository (e.g. GitHub). See the Nature Portfolio guidelines for submitting code & software for further information.

# Data

Policy information about availability of data

All manuscripts must include a data availability statement. This statement should provide the following information, where applicable:

- Accession codes, unique identifiers, or web links for publicly available datasets
- A description of any restrictions on data availability
- For clinical datasets or third party data, please ensure that the statement adheres to our policy

The raw RNA sequencing data are publicly available in dbGaP repository under accession code phs003615.v1.p1.
The RSEM expected gene counts are publicly available in GEO database under accession code GSE248014 [ https://www.ncbi.nlm.nih.gov/geo/query/acc.cgi?acc=GSE248014] .
The CyTOF data are publicly available in Zenodo under doi: 10.5281/zenodo.12802613 [https://doi.org/10.5281/zenodo.12802613].
The remaining data are available within the Article, Supplementary Information or Source Data files. Source data are provided with this paper.

CODE AVAILABILITY
The code associated with this manuscript is available on Zenodo: https://zenodo.org/doi/10.5281/zenodo.12795850. The mIHC analysis protocol and code are available on protocols.io: dx.doi.org/10.17504/protocols.io.n92ldmmznl5b/v2

The authors declare that the minimal data set and source data for clinical data for this study cannot be shared publicly due to ethical and legal restrictions on sharing de-identified data that aligns with the consent of research participants.  Current JHU compliance policies require data with no direct consent for public open access sharing be under restricted access. We provide access through dbGAP, an established repository for clinical data that provides open access without a fee restricted to approved researchers under a Data Use Agreement. JHU compliance policy for dbGAP requires additional anonymization of certain demographics, including the use of age ranges and limiters to outlier values for weight, height, and certain rare diseases, while retaining sufficient value for reference and validation of results. Researchers can request more detailed data from the corresponding author shared through an approved collaboration arrangement.

# Research involving human participants, their data, or biological material

Policy information about studies with human participants or human data. See also policy information about sex, gender (identity/presentation), and sexual orientation and race, ethnicity and racism.

| | |
|---|---|
| Reporting on sex and gender | Thirteen (43%) of the patients were female. Both male and female participants could be included in the trial, but only biologically female patients participated in the trial. The findings from this trial don't apply to only one gender. |
| Reporting on race, ethnicity, or other socially relevant groupings | Twenty-eight (93%) of patients were White and 2 (7%) were Black or African American. 1 (3%) were Hispanic or Latin, 27 (90%) were not Hispanic or Latin, and 2 (7%) did not have available ethnicity data. Race and ethnicity data were based on self-report data.  Baseline demographics are provided in the Table 1. |
| Population characteristics | All patient demographics are shown in detail in Table 1. in summary, we enrolled patients at least 18 years old with histologically or cytologically confirmed diagnosis of pancreatic adenocarcinoma that had progression after prior lines of therapy; Median age was 63.5 (range 32-79). Forty-three 5 of the patients identified themselves as female. Thirty-seven % of patients had received one prior line of chemotherapy, and 64% had received 2 or more prior lines of chemotherapy. |
| Recruitment | Eligible patients were 18 years of age or older, had an Easter Cooperative Oncology Group performance status score of q o1 (on a 5-point scale in which higher numbers reflect greater disability), and had adequate hematological, renal, and hepatic function. Eligible patients had histologically or cytologically confirmed pancreatic adenocarcinoma, locally advanced or metastatic, and had received one or two prior lines of chemotherapy; participants were also required to have measurable disease and accessible non-bone tumor 416 lesions for serial core biopsies. Patients with a history of active autoimmune disease or uncontrolled intercurrent illness were excluded.  The attached Clinical protocol provides full details on inclusion and exclusion criteria. |
| Ethics oversight | All procedures were conducted in accordance with the Declaration of Helsinki and the International Conference on Harmonization Guidelines for Good Clinical Practice. The protocol and all amendments were reviewed by the scientific review committee and institutional review board at the Johns Hopkins Sidney Kimmel Cancer Center. All patients provided written informed consent before enrollment. |

Note that full information on the approval of the study protocol must also be provided in the manuscript.

# Field-specific reporting

Please select the one below that is the best fit for your research. If you are not sure, read the appropriate sections before making your selection.

☒ Life sciences ☐ Behavioural & social sciences ☐ Ecological, evolutionary & environmental sciences

For a reference copy of the document with all sections, see nature.com/documents/nr-reporting-summary-flat.pdf

# Life sciences study design

All studies must disclose on these points even when the disclosure is negative.

| | |
|---|---|
| Sample size | This was an open-label, single-arm, phase 2 clinical trial to assess the safety and efficacy of entinostat plus nivolumab in patients with advanced stage pancraetci cancer. A Simon's two-stage design was applied to test the null hypothesis that the true ORR is 5% or less (not considered clinically compelling for this combination). This design yields 80% power at a one-sided type I error rate of 5% when the trueresponse rate is 20%. |
| Data exclusions | No data were excluded from the analysis |
| Replication | N/A - The clinical trial was a non-randomized, single arm study. Correlative analyses weer based on all samples available. |
| Randomization | N/A - The clinical trial was a non-randomized, single arm study. |
| Blinding | The clinical trial was a single arm study and response outcome were not blinded to the investigators during data collections or analyses |

# Reporting for specific materials, systems and methods

We require information from authors about some types of materials, experimental systems and methods used in many studies. Here, indicate whether each material, system or method listed is relevant to your study. If you are not sure if a list item applies to your research, read the appropriate section before selecting a response.

## Materials & experimental systems

| n/a | Involved in the study | |
|---|---|---|
| ☐ | ☒ | Antibodies |
| ☒ | ☐ | Eukaryotic cell lines |
| ☒ | ☐ | Palaeontology and archaeology |
| ☒ | ☐ | Animals and other organisms |
| ☐ | ☒ | Clinical data |
| ☒ | ☐ | Dual use research of concern |
| ☒ | ☐ | Plants |

## Methods

| n/a | Involved in the study | |
|---|---|---|
| ☒ | ☐ | ChIP-seq |
| ☒ | ☐ | Flow cytometry |
| ☒ | ☐ | MRI-based neuroimaging |

## Antibodies

| | |
|---|---|
| Antibodies used | All antibodies used in the study are described in detail in the supplementary files |
| Validation | The following manuscripts detail antibody validation and protocols for IHC: Tsujikawa, Takahiro, et al. "Quantitative multiplex immunohistochemistry reveals myeloid-inflamed tumor-immune complexity associated with poor prognosis."Cell reports. 19.1 (2017): 203-217.<br><br>Banik, Grace, et al. "High-dimensional multiplexed immunohistochemical characterization of immune contexture in human cancers."Methods in enzymology635 (2020): 1-20.<br><br>Liudahl SM, Betts CB, Sivagnanam S, et al. "Leukocyte Heterogeneity in Pancreatic Ductal Adenocarcinoma: Phenotypic and Spatial Features Associated with Clinical Outcome." Cancer Discov. 2021;11(8):2014-2031. doi:10.1158/2159-8290.CD-20-0841 |

## Clinical data

Policy information about clinical studies

All manuscripts should comply with the ICMJEguidelines for publication of clinical research and a completedCONSORT checklist must be included with all submissions.

| | |
|---|---|
| Clinical trial registration | This trial is registered under the name 'A Clinical Trial of Entinostat in Combination With Nivolumab for 404 Patients With Previously Treated Unresectable or Metastatic Cholangiocarcinoma and Pancreatic Adenocarcinoma' (registration no. NCT03250273). |
| Study protocol | Protocol is available as Supplementary information |
| Data collection | This was an open-label, single-arm, phase 2 clinical trial conducted at the Sidney Kimmel Comprehensive Cancer Center at Johns Hopkins, Baltimore, Maryland From November 2017 to November 2020, a total of 30 patients with unresectable or metastatic, previously treated pancreatic cancer were enrolled at the Johns Hopkins Sidney Kimmel Comprehensive Cancer Center in Baltimore, Maryland. |
| Outcomes | The primary trial endpoint focused on the objective response rate (ORR) assessed by RECIST 1.1.<br>A Simon's two-stage, minimax design was used to test the null hypothesis that the true ORR is 5% or less (not considered clinically compelling for this combination). In the first stage, 13 subjects were accrued. If there were no responders among the first 13 subjects, the study was terminated for futility. Otherwise, 14 additional subjects were accrued to target 27 treated and 492 response evaluable subjects. The null hypothesis was rejected if four or more responses were observed in 27 subjects. The probability of stopping the trial early for futility was 51% if the true ORR was 5% or less. This design yielded 80% power at a one-sided type I error rate of 5% when the true response rate was 20%. An exact binomial test was used to evaluate the primary question of whether the response rate for combination therapy exceeds the historical rate (5%) for the single agent. Response rates were reported with exact confidence intervals.<br>The primary analysis was based on the intention to treat (ITT) population, while the per-protocol analysis was defined in patients who received at least one restaging scan.<br><br>Secondary endpoints include safety, progression-free survival (PFS), and overall survival (OS). Exploratory objectives included biomarker and immunological analysis of serial tumor biopsies and peripheral blood samples. All the patients were monitored for adverse events, according to the National Cancer Institute Common Terminology Criteria for Adverse Events, version 4.0. |

## Plants

| | |
|---|---|
| Seed stocks | *Report on the source of all seed stocks or other plant material used. If applicable, state the seed stock centre and catalogue number. If plant specimens were collected from the field, describe the collection location, date and sampling procedures.* |
| Novel plant genotypes | *Describe the methods by which all novel plant genotypes were produced. This includes those generated by transgenic approaches, gene editing, chemical/radiation-based mutagenesis and hybridization. For transgenic lines, describe the transformation method, the number of independent lines analyzed and the generation upon which experiments were performed. For gene-edited lines, describe the editor used, the endogenous sequence targeted for editing, the targeting guide RNA sequence (if applicable) and how the editor was applied.* |
| Authentication | *Describe any authentication procedures for each seed stock used or novel genotype generated. Describe any experiments used to assess the effect of a mutation and, where applicable, how potential secondary effects (e.g. second site T-DNA insertions, mosiacism, off-target gene editing) were examined.* |
